# Cell type and gene expression deconvolution with BayesPrism enables Bayesian integrative analysis across bulk and single-cell RNA sequencing in oncology

**DOI:** 10.1038/s43018-022-00356-3

**Published:** 2022-04-25

**Authors:** Tinyi Chu, Zhong Wang, Dana Pe’er, Charles G. Danko

**Affiliations:** 1 1Baker Institute for Animal Health, College of Veterinary Medicine, Cornell University, Ithaca, NY USA; 2 Graduate field of Computational Biology, Cornell University, Ithaca, NY USA; 3 Computational and Systems Biology Program, Sloan Kettering Institute, Memorial Sloan Kettering Cancer Center, New York, NY USA; 4 School of Software Technology, Dalian University of Technology, Dalian, China; 5 Department of Biomedical Sciences, College of Veterinary Medicine, Cornell University, Ithaca, NY USA

**Keywords:** Statistical methods, Cancer genomics, Cancer

## Abstract

Inferring single-cell compositions and their contributions to global gene expression changes from bulk RNA sequencing (RNA-seq) datasets is a major challenge in oncology. Here we develop Bayesian cell proportion reconstruction inferred using statistical marginalization (BayesPrism), a Bayesian method to predict cellular composition and gene expression in individual cell types from bulk RNA-seq, using patient-derived, scRNA-seq as prior information. We conduct integrative analyses in primary glioblastoma, head and neck squamous cell carcinoma and skin cutaneous melanoma to correlate cell type composition with clinical outcomes across tumor types, and explore spatial heterogeneity in malignant and nonmalignant cell states. We refine current cancer subtypes using gene expression annotation after exclusion of confounding nonmalignant cells. Finally, we identify genes whose expression in malignant cells correlates with macrophage infiltration, T cells, fibroblasts and endothelial cells across multiple tumor types. Our work introduces a new lens to accurately infer cellular composition and expression in large cohorts of bulk RNA-seq data.

## Abstract (web summary)

Danko and colleagues develop BayesPrism, a bulk RNA sequencing deconvolution tool to infer cell type composition and cell-specific expression levels across clinical cancer datasets.

## Main

Cell–cell interactions are highly complex and can strongly impact cell behavior in biological contexts, often with medical ramifications. A quintessential example is that between malignant cells and diverse nonmalignant cell types within the tumor microenvironment (TME)^1,2^. Numerous studies over the past two decades have revealed interactions between cells in the TME that promote diverse functions, including angiogenesis^3^, metastasis^4^ and immunosuppression^5^. Nonmalignant cells can differ markedly among patients and tumor types^6^, and certain nonmalignant cell populations are used as clinical biomarkers^7^ and therapeutic targets^8^. These studies motivate the direct measurement of cell types within tissues.

Two layers of information are critical for understanding tumor composition: (1) the proportion of each cell type and (2) the levels of gene expression in each cell type. The rise of single-cell RNA sequencing (scRNA-seq) technologies has recently enabled direct, genome-wide measurement of the transcriptome in individual cells within the TME and characterization of their heterogeneity. However, the cost of scRNA-seq and requirements for high-quality tissue limit the number of patient samples that can be assayed^9^. Moreover, scRNA-seq is susceptible to technical biases in cell capture^9^, which confound the recovery of cell type composition.

As an alternative, cell type abundance can be inferred from bulk RNA-seq data using regression on a reference expression matrix constructed from a set of arbitrarily defined marker genes^10–14^. Pioneering methods for cell type deconvolution have demonstrated that it is possible to infer the abundance of multiple cell types in the TME. However, existing deconvolution methods make restrictive assumptions about the difference in distribution between the reference and bulk sample. These assumptions are often violated by both technical (for example, different platforms used for reference and bulk sequencing) and biological (for example, heterogeneity in gene expression within constituent cell types) differences between bulk and reference data. Critically, cell type deconvolution methods have not fully supported prediction of gene expression in a heterogeneous population of tumor cells. Thus, existing methods fail to address these key questions: how do malignant cells affect the composition of nonmalignant cells in the TME? Which genes are correlated with these interactions? To answer these questions, we need a model that can accurately represent cell type fraction and cell-type-specific expression profiles in each bulk sample, and can accommodate differences between the single-cell reference and bulk.

Here, we present BayesPrism, a Bayesian model that jointly infers the posterior distribution of cell type fractions and gene expression from bulk RNA-seq data using an scRNA-seq reference as prior information. By explicitly modeling and marginalizing out the differences in gene expression between single-cell reference and bulk data, BayesPrism substantially outperforms leading methods in the inference of cell type fractions in both tumor and nontumor settings. We demonstrate the utility of our approach on a large dataset of 1,412 bulk RNA-seq and 85 scRNA-seq samples in glioblastoma (GBM), head and neck squamous cell carcinoma (HNSCC) and skin cutaneous melanoma (SKCM). Our work introduces a powerful new tool for integrative analysis of bulk and scRNA-seq data.

## Results

### Bayesian inference of cell type fraction and gene expression

**Fig. 1:**
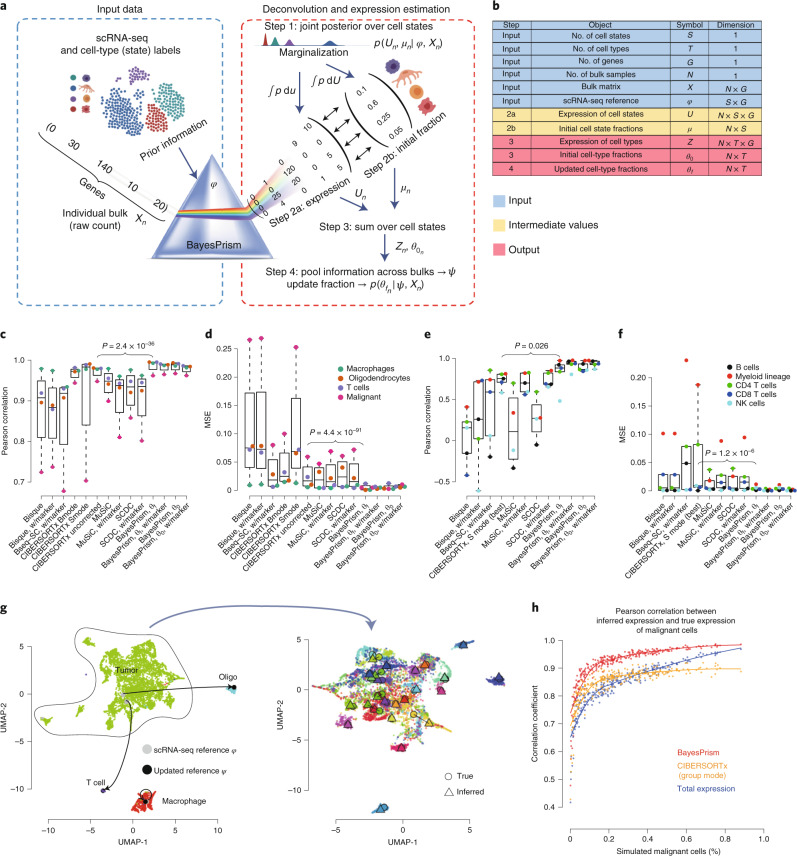
BayesPrism algorithm flow and performance validation. **a**, Algorithmist flow of the deconvolution module of BayesPrism. **b**, Variables and their dimensions shown in **a**. **c**–**f**, Boxplots showing the cell-type-level Pearson’s correlation coefficient (**c**,**e**) and MSE (**d**,**f**) for deconvolution of GBM28 pseudo-bulks using refGBM8 (**c**,**d**), and bulk RNA-seq human whole blood samples with ground truth measured by flow cytometry (**e**,**f**). Boxes mark the 25th percentile (bottom), median (central bar) and 75th percentile (top). Whiskers represent extreme values within 1.5-fold of interquartile range. One-sided *P* values are shown for cell type fractions inferred by BayesPrism (updated *θ* using marker-free mode) and those by the second-best methods ranked by median value. *T*-test was used for MSE, and *z*-test was performed on Fisher’s *z*-transformed cell-type-level correlation coefficients (Methods). **c**,**d**, Statistics were computed for 1,350 pseudo-bulk RNA-seq samples simulated using scRNA-seq from 27 patients with GBM having more than ten malignant cells for all methods, except CIBERSORTx. For CIBERSORTx and its comparison with BayesPrism, statistics were computed using 270 downsampled pseudo-bulks across 27 patients. **e**,**f**, Statistics were computed using 12 bulk RNA-seq samples from independent healthy adults. **g**, Uniform manifold approximation and projection (UMAP) visualization showing expression of individual cells in GBM28. The expression profiles of nonmalignant cells before (gray) and after information pooling (black) were projected onto the UMAP manifold of scRNA-seq (left). Malignant cells in patients with more than ten malignant cells (*n* = 27) are visualized on the zoomed-in UMAP (right) and are colored by patient. The inferred expression profile, shown as △, and the averaged expression profile from scRNA-seq for each patient, shown as ○, are projected onto the UMAP manifold. **h**, Scatter plot showing Pearson’s correlation between average expression of malignant cells in pseudo-bulk and that estimated by BayesPrism (red) and CIBERSORTx group mode (orange) or the undeconvolved simulated bulk (blue), as a function of the fraction of malignant cells in a subsampled set (*n* = 270). Source data

**Extended Data Fig. 1:**
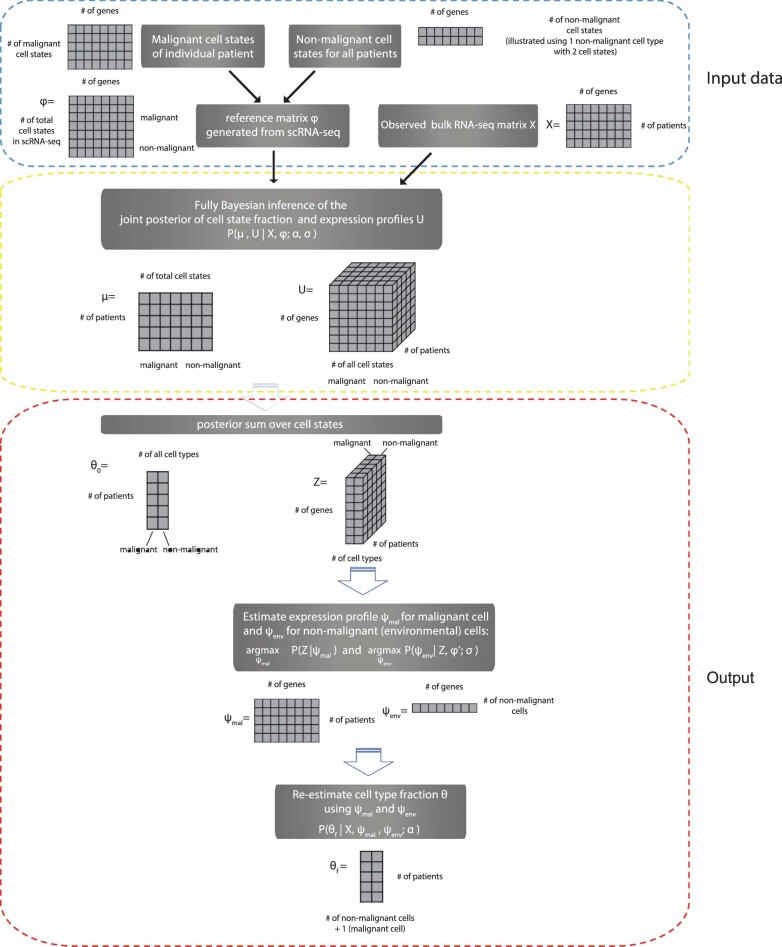
A detailed algorithmic flow of the deconvolution module of BayesPrism. Gray grids show the dimension of the variables used or inferred in each step.

BayesPrism uses an scRNA-seq reference to infer two statistics from each bulk RNA-seq sample: (1) the proportion of reads derived from each cell type, which we assume is proportional to the fraction of that cell type; and (2) the expression level of genes in each cell type (Fig. 1a,b, Extended Data Fig. 1 and Supplementary Note 1). The most challenging aspect of cellular deconvolution is accounting for various sources of uncertainty, including technical and biological batch variation, in gene expression between bulk and scRNA-seq reference data. To account for these uncertainties, BayesPrism adopts a Bayesian strategy that models prior distribution using scRNA-seq, and infers a joint posterior distribution of cell type proportion and gene expression in each cell type and bulk sample conditional on each observed bulk. As a result, the uncertainty in each estimate can be marginalized out from the joint posterior.

BayesPrism accommodates multiple gene expression subtypes of the same cell type (hereafter referred to as ‘cell states’). Internally, BayesPrism treats cell types and states in the same manner, and returns the posterior sum over different cell states defined by the scRNA-seq dataset to represent the fraction and expression of each cell type. This strategy is useful in modeling heterogeneous cell types, including both malignant and nonmalignant cell types in the TME.

BayesPrism is implemented in an efficient algorithm (Methods and Supplementary Note 2) consisting of four major steps:

1. BayesPrism first infers a joint posterior distribution of the cell state proportion and gene expression, *μ*_*n*_ and *U*_*n*_, respectively, conditional on the observed single-cell reference *φ* (obtained by summing over the count matrix of each cell state followed by normalization by total count), and read counts of bulk expression *X*_*n*_ of the *n*th bulk sample—that is, *p*(*μ*_*n*_, *U*_*n*_ *| φ, X*_*n*_)—using Gibbs sampling.
2. For each bulk sample *n*, BayesPrism estimates (step 2a) the gene expression matrix of each cell state, *U*_*n*_, and (step 2b) the proportion of each cell state, *μ*_*n*_, by marginalization of the joint posterior and reporting the posterior mean. Explicit modeling of the cell-type-specific expression value in each bulk makes BayesPrism robust to technical batch effects and biological variation between the scRNA-seq reference and bulk (Supplementary Note 3).
3. For each bulk sample *n*, BayesPrism estimates the gene expression matrix of each cell type, *Z*_*n*_, and the proportion of each cell type, $\theta _{0_n}$, by summing the posteriors over cell states (estimated in step 2) within each cell type.
4. Optionally, BayesPrism updates the reference matrix *φ* by pooling information across bulk samples from *Z* to improve estimates of cell type fractions. The updated reference matrix, *ψ*, represents the multinomial distribution parameters describing the distribution of *Z*. Two strategies are used to infer *ψ* depending on whether the cell type is malignant—that is, of high heterogeneity—or not (Methods). BayesPrism then uses the updated prior distribution parameterized by *ψ* to re-estimate the marginal posterior of cell type fraction for each bulk sample—that is, *p*($\theta _{f_n}$*| ψ*, *X*_*n*_). Sharing information across bulk samples often provides higher accuracy regarding problems with batch effects.

### BayesPrism improves the accuracy of cell type deconvolution

**Extended Data Fig. 2:**
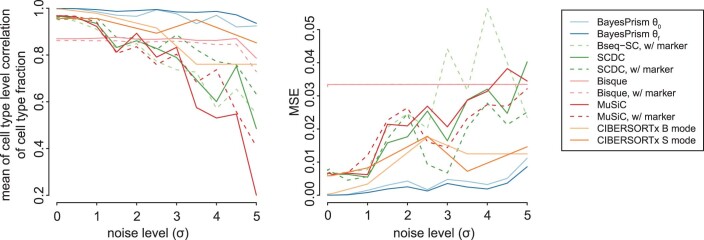
Comparison between BayesPrism and other deconvolution methods using simulated noise. Benchmark using simulated noise. Line plots show the cell type-level Pearson’s correlation coefficient (**left**) and MSE (**right**) as a function of the noise level.

We benchmarked the robustness of cell type deconvolution against a simulated batch effect between the scRNA-seq reference and bulk datasets. We constructed pseudo-bulk RNA-seq data that differed from the reference scRNA-seq dataset by a log-normally distributed multiplicative noise term (Methods and Supplementary Table 1). We compared Pearson correlation and mean squared error (MSE) between the ground truth and cell type proportions estimated using five different deconvolution methods^10–14^. BayesPrism was nearly invariant to simulated noise and outperformed existing methods by up to an order of magnitude as noise increased (Extended Data Fig. 2; see Supplementary Note 3 for mathematical arguments outlining why BayesPrism is invariant to linear noise).

**Extended Data Fig. 3:**
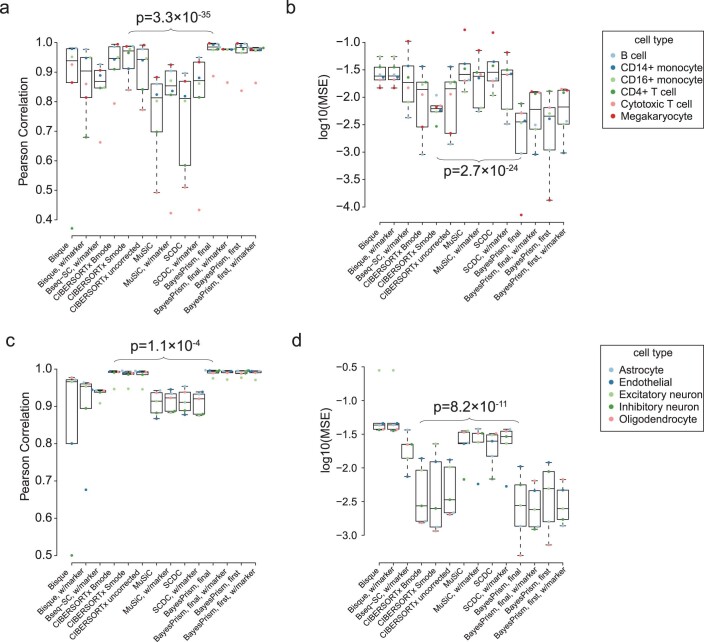
Comparison between BayesPrism and other deconvolution methods on pseudo-bulks across different sequencing platforms and biological samples. Boxplots show the cell type-level Pearson’s correlation coefficient and MSE for the deconvolutions of pseudo-bulk human PBMC scRNA-seq (**a** and **b**, n=200 pseudo-bulks simulated using 253 single cells collected from one healthy adult) and mouse cortex single nucleus-seq (**c** and **d**, n=200 pseudo-bulks simulated using 295 single cells collected from one mouse). Boxes mark the 25th percentile (bottom of box), median (central bar), and 75th percentile (top of box). Whiskers represent extreme values within 1.5 fold of the inter quartile range. One-sided p values were shown for cell type fractions inferred by BayesPrism (updated θ using the marker free mode) and those by the second-best methods ranked by the median value. T test was used for MSE and z test was performed on Fisher’s Z-transformed cell type-level correlation coefficients (see Methods). Source data

**Extended Data Fig. 4:**
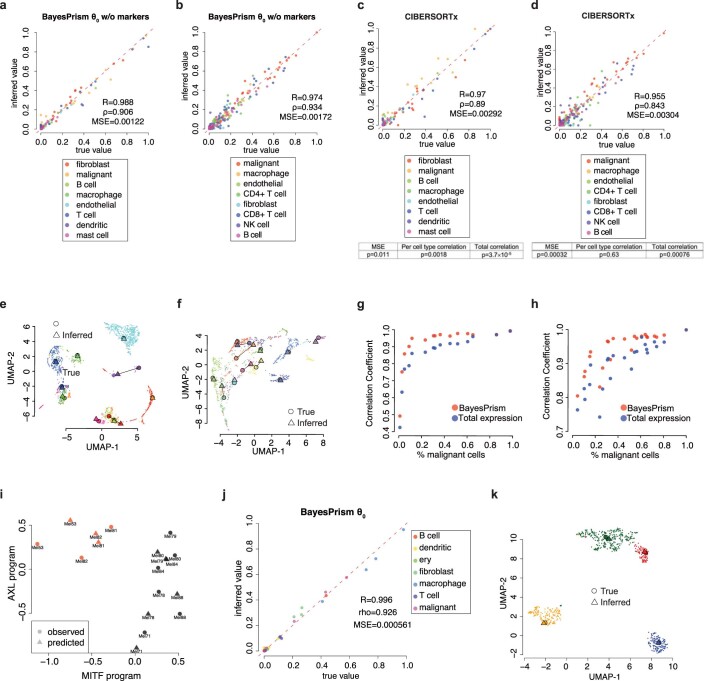
Performance of BayesPrism in inferring cell type composition and gene expression in malignant cells on the leave-one-out pseudo-bulk data of HNSCC, SKCM and OV. (**a**-**b**) Scatter plots show θ_0_ in (**a**) HNSCC and (**b**) SKCM versus the ground truth in pseudo-bulk. (**c**-**d**) Scatter plots show the CIBERSORTx inferred cell type fraction in (**c**) HNSCC and (**d**) SKCM versus the ground truth in pseudo-bulk. Tables below show the one-sided p values for cell type fractions inferred by BayesPrism and CIBERSORTx. T test was used for MSE and z test was performed on Fisher’s Z-transformed cell type-level correlation coefficients (see Methods). (**e**-**f**) UMAP shows the expression profile of individual malignant cells in scRNA-seq of (**e**) HNSCC and (**f**) SKCM colored by patient ID. Patients with >50 malignant cells and cells with reads detected for >3000 genes are shown. The inferred expression profile, shown as △, and the averaged expression profile from scRNA-seq for each patient, shown as ○, are projected onto the UMAP manifold. (**g**-**h**) Scatter plot shows Pearson’s correlation coefficient between inferred expression and that of the averaged expression from malignant cells in scRNA-seq of (**g**) HNSCC and (**h**) SKCM as a function of the fraction of malignant cells in each simulated data. The correlation coefficient was computed on DESeq2 variance-stabilized transformed values. Red marks the correlation inferred by BayesPrism, while blue marks that of total expression of the simulated data. (**i**) Scatter plot shows the AXL and MITF program scores of malignant cells from SKCM in the leave-one-out test, calculated by the mean of Z scores over the corresponding marker genes. ○ marks the average expression of malignant cells in each patient from the scRNA-seq data, while △ marks the inferred expression profile of malignant cells in each patient. (**j**) Scatter plots show θ_0_ in OV versus the ground truth in pseudo-bulk. (**k**) UMAP shows the expression profile of individual malignant cells in scRNA-seq of OV colored by patient ID. The inferred expression profile, shown as △, and the averaged expression profile from scRNA-seq for each patient, shown as ○, are projected onto the UMAP manifold.

To assess whether BayesPrism improved deconvolution performance in a more realistic setting, we next generated pseudo-bulk data by combining reads from single cells in three different settings: (1) peripheral blood mononuclear cells (PBMCs) and mouse brain cortex samples from different healthy subjects and sequenced by different scRNA-seq platforms, representing technical batch effects with small amounts of biological variation (Extended Data Fig. 3); (2) leave-one-out tests in datasets of three human cancer types generated by the same sequencing platforms representing biological variation with small amounts of technical noise (Extended Data Fig. 4a–d); and (3) GBM datasets generated from different cohorts using different sequencing platforms representing a mixture of both effects: full-length SMART-seq2 data consisting of 28 patients as a surrogate for bulk RNA-seq (GBM28) and 3′ end-enriched tag clusters obtained using a microwell-based platform from eight patients as the reference (refGBM8) (Fig. 1c,d).

BayesPrism significantly outperformed all existing methods in all three settings in 63 of 64 tests (*P* < 0.05; Supplementary Notes 4 and 5). In the GBM dataset (the third setting), BayesPrism was particularly more efficient than CIBERSORTx in estimating the proportion of malignant cells, in which gene expression was a poor match for the reference data and consistent with our expectation that the Bayesian method will provide the highest performance advantage in the presence of substantial gene expression variation between the bulk and reference data (Fig. 1c and Supplementary Fig. 1). Separate analyses also found that BayesPrism was robust to cell types missing from the scRNA-seq reference, as well as to the number of cells and scRNA-seq reference samples (Supplementary Note 6).

As a final performance benchmark, we deconvolved real bulk RNA-seq data using a ground truth obtained by orthogonal strategies. We obtained bulk RNA-seq data from 12 whole-blood samples analyzed in parallel using flow cytometry^12^. Using PBMC scRNA-seq data as a reference, BayesPrism obtained more accurate estimates of five cell types in the bulk sample than other deconvolution methods (*P* < 5.46 × 10^–4^ on MSE, *P* < 0.03 on correlation coefficients) (Fig. 1e,f). Taken together, these benchmarks demonstrate that BayesPrism improved deconvolution performance in realistic settings.

### BayesPrism estimates gene expression in unobserved patients

We asked whether BayesPrism would accurately recover gene expression in heterogeneous cell types. We first focused on the recovery of gene expression in malignant cells where cross-patient heterogeneity makes prediction of gene expression a challenging problem. We estimated cell types and gene expression in SMART-seq2 pseudo-bulk data from 28 GBMs. We used a microwell-based scRNA-seq reference from eight GBMs, which tested the accuracy of BayesPrism in the presence of both biological and technical variation between the bulk and scRNA-seq reference data. Gene expression estimates for malignant cells in the pseudo-bulk samples (*ψ*_mal_) were highly similar to the known ground truth (Fig. 1g, right).

**Extended Data Fig. 5:**
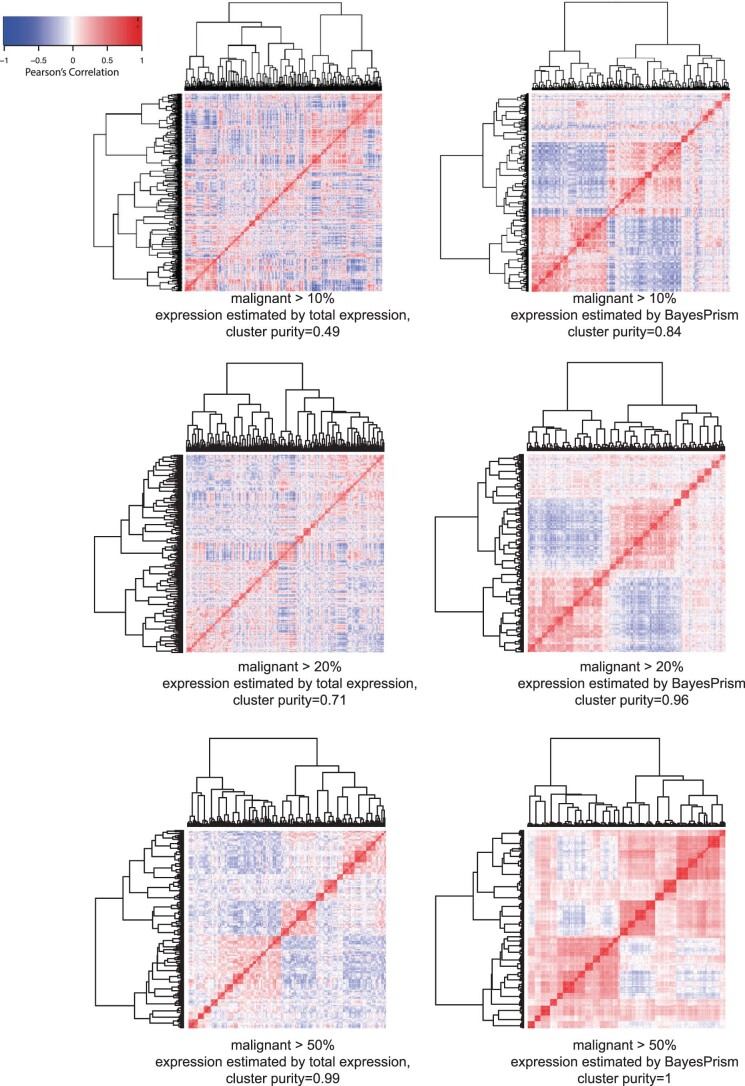
BayesPrism expression estimates group malignant cells from the same patient. Heatmaps show the pairwise Pearson correlation matrix between gene expression computed for each pair of simulated pseudo-bulk samples with the fraction of malignant cells greater than 10% (top row), 20% (mid) and 50% (bottom). Simulated samples were constructed by drawing a random proportion of each non-malignant cell type and sampling the malignant cells from one of 27 GBM patients. Vectors used for computing the Pearson correlation are of length equal to the total number of genes used to perform deconvolution, denoting zero centered variance-stabilizing transformed reads. Simulated pseudo-bulk samples are grouped by hierarchical clustering, as shown by the dendrogram. Left column: Correlations using total expression from bulk samples without any correction; Right column: Correlations over the same set of samples and genes as in the left column but using deconvolved expression profiles for malignant cells in each sample.

**Extended Data Fig. 6:**
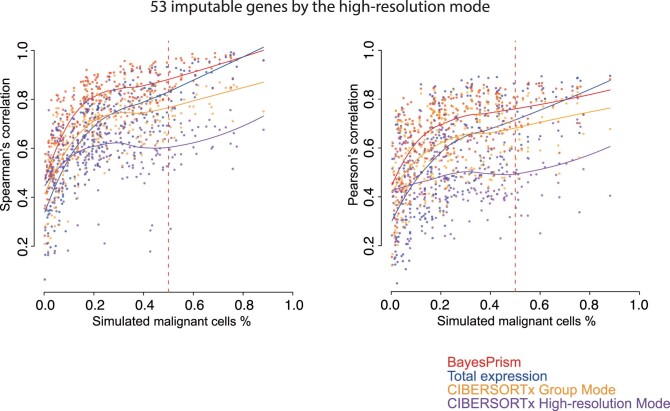
Comparison between BayesPrism and the two different modes of expression inference by CIBERSORTx. Scatter plot shows Spearman’s correlation (left), and Pearson’s correlation (right), between gene expression estimated by BayesPrism (red), total bulk (blue), CIBERSORTx group mode (orange) or CIBERSORTx high resolution mode (purple) and the average expression from malignant cells in scRNA-seq as a function of the fraction of malignant cells in the dataset containing the 270 simulated samples. The correlation coefficient was calculated on 53 imputable genes by the high-resolution mode out of the top 1000 most variable genes in malignant cells. Spearman’s correlation coefficients were calculated on untransformed gene expression values, while Pearson’s correlation coefficients were calculated on variance-stabilizing transformed gene expression values.

Next, we asked how the accuracy of gene expression estimates would be affected by the proportion of malignant cells. We sampled random proportions of each cell type, drawing malignant cells from a single patient. The correlation between BayesPrism gene expression estimates and known ground truth was >0.95 for tumors, with >50% purity (Fig. 1h). Moreover, different samples simulated from the same patient produced estimates highly concordant with each other (Extended Data Fig. 5), suggesting that gene expression deconvolved by BayesPrism can accurately recover the underlying structure of gene expression in malignant cells from bulk samples. Gene expression estimates were substantially more accurate using BayesPrism than either CIBERSORTx or bulk tumor with no deconvolution (Fig. 1h and Extended Data Fig. 6).

Next, we expanded our analysis to a second cancer type, SKCM, and examined whether BayesPrism would recover expression differences characteristic of the AXL and MITF malignant cell states. We compared BayesPrism expression estimates with AXL and MITF marker genes reported previously in the literature^15^ using the leave-one-out benchmark design described above. BayesPrism reproduced both the AXL and MITF subtypes with accuracy similar to the original scRNA-seq data (Extended Data Fig. 4i). Thus, BayesPrism accurately recovered sample-specific features of gene expression in heterogeneous cell types.

**Extended Data Fig. 7:**
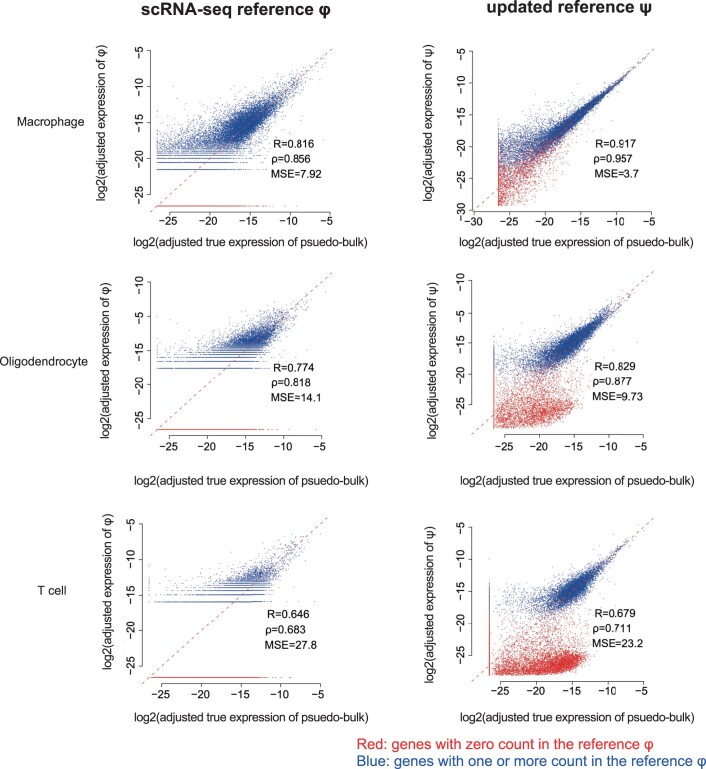
BayesPrism improves correlations between gene expression in reference and ground truth pseudo-bulk data in non-malignant cells. Scatter plots show log2 gene expression in non-malignant cells from the original scRNA-seq reference *φ* (the left column) and the updated reference *ψ* after the information pooling step (the right column) versus the mean expression in scRNA-seq used to generate pseudo-bulk. Genes with zero expression counts in the scRNA-seq reference are colored in red, and those with non-zero expression counts are colored in blue.

**Extended Data Fig. 8:**
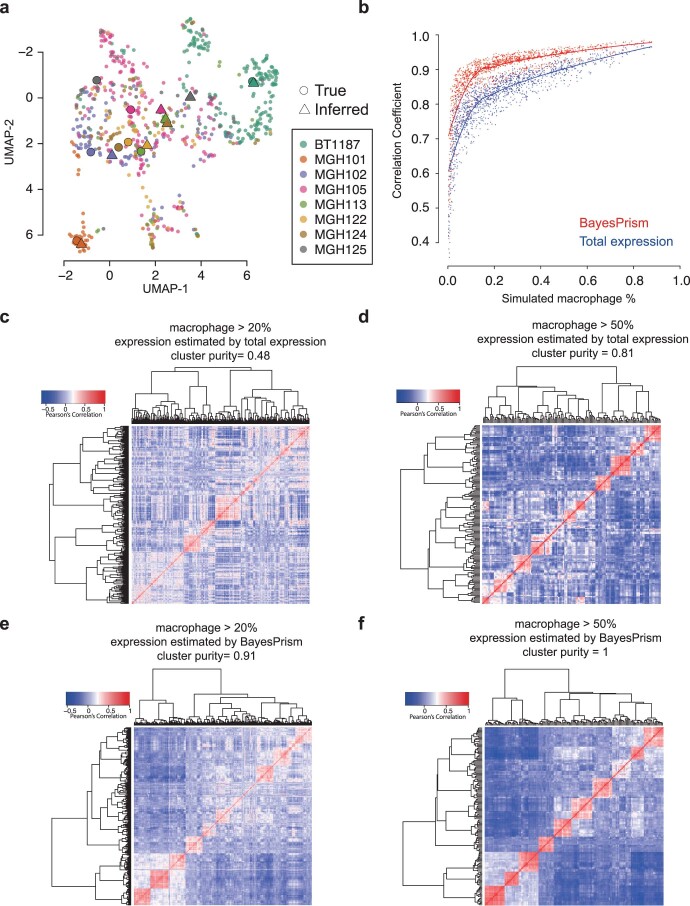
BayesPrism accurately recovers the heterogeneity in the expression of macrophages. (**a**) UMAP visualization shows the expression of individual macrophages in the pseudo-bulk GBM28 dataset. The expression profile inferred by BayesPrism, shown as △, and the averaged expression profile from scRNA-seq for each patient, shown as ○, are projected onto the UMAP manifold. (**b**) Scatter plot shows Pearson’s correlation of reads summed across macrophages in the pseudo-bulk (ground truth) and gene expression deconvolved by BayesPrism (red) or undeconvovled pseudo-bulk (blue) as a function of the fraction of macrophages in the simulated pseudo-bulk (N=1,350). The correlation was computed using variance-stabilizing transformed reads. (**c**-**f**) Heatmap shows the pairwise Pearson correlation matrix between gene expression computed for each pair of simulated pseudo-bulk samples with macrophage fractions greater than 20% (**c**, **e**) and 50% (**d**, **f**). Simulated samples were obtained by drawing a random proportion of each cell type from the GBM-28 dataset, while sampling macrophages from an individual macrophage sub-cluster. Simulated pseudo-bulk samples are grouped by hierarchical clustering, as shown by the dendrogram. (**c-d)** Correlations estimated using the total gene expression without any correction. (**e-f**) Correlations over the same set of samples and over the same set of genes as in **c** and **d**, but using BayesPrism deconvolved expression profiles for macrophages in each sample.

We asked whether BayesPrism expression estimates would recover gene expression in nonmalignant cell types. Gene expression estimates for macrophages, T cells and oligodendrocytes (*ψ*_env_) better matched the known ground truth than the scRNA-seq prior in the 27 GBM pseudo-bulk samples (Fig. 1g, left and Extended Data Fig. 7). To assess whether we could recover heterogeneity between patients for nonmalignant cells, we subclustered macrophages in scRNA-seq data and sampled a random proportion of macrophages from one cluster to generate each pseudo-bulk sample that was otherwise similar to those obtained for malignant cells above (Supplementary Note 4). BayesPrism accurately recovered subtle variation in gene expression, which allowed the recovery of macrophage cell states between simulated pseudo-bulk samples (Extended Data Fig. 8). We conclude that BayesPrism can recover the average gene expression patterns of each cell type from bulk RNA-seq data.

### Survival impact of infiltrating immune cell types and states

**Fig. 2:**
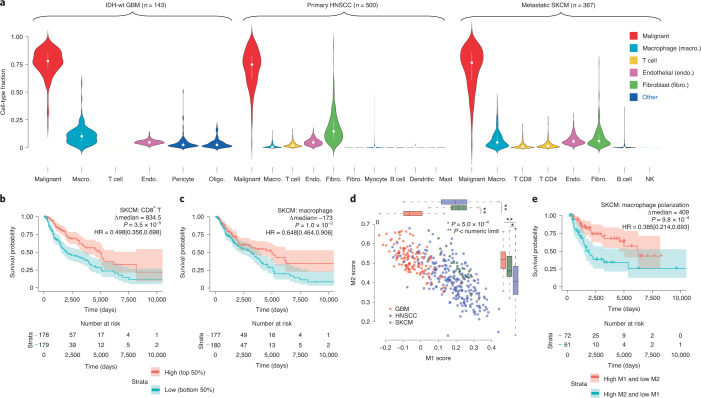
Association between prognosis and either cell type fraction or cell state of nonmalignant cells in three TCGA tumor types. **a**, Violin plots showing distribution of cell type fraction in each tumor type. Median fractions are denoted by white dots and upper/lower quartiles by bars. Oligo., oligodendrocytes. **b**,**c**, Kaplan–Meier plots showing survival associations with infiltration of T cells (**b**) and macrophages (**c**) in SKCM. Δmedian, median survival time in the high group/median survival time in the low group. **d**, Scatter plot showing correlation between BayesPrism expression estimates in macrophages and M1 and M2 macrophage subtype scores across three tumor types. Boxes mark the 25th percentile (bottom), median (central bar) and 75th percentile (top); whiskers represent extreme values within 1.5-fold of interquartile range. Statistical significance was determined by one-way analysis of variance (*P* < numeric limit and degrees of freedom = 2 for both M1 and M2 scores; M1, *F* = 203.44; M2, *F* = 63.226), followed by reporting of Tukey’s honestly significant difference-adjusted *P* values. *n*(GBM) = 127, *n*(HNSCC) = 26 and *n*(SKCM) = 225 independent TCGA bulk tumor samples. **e**, Kaplan–Meier plots showing survival associations with the M1/M2 polarization state of macrophages in SKCM. **b**,**c**,**e**, *P* values were derived from log-rank test, HR was defined by high/low and 95th percentile confidence intervals are shown in square brackets. Transparent colors denote 95% confidence bands. Source data

**Extended Data Fig. 9:**
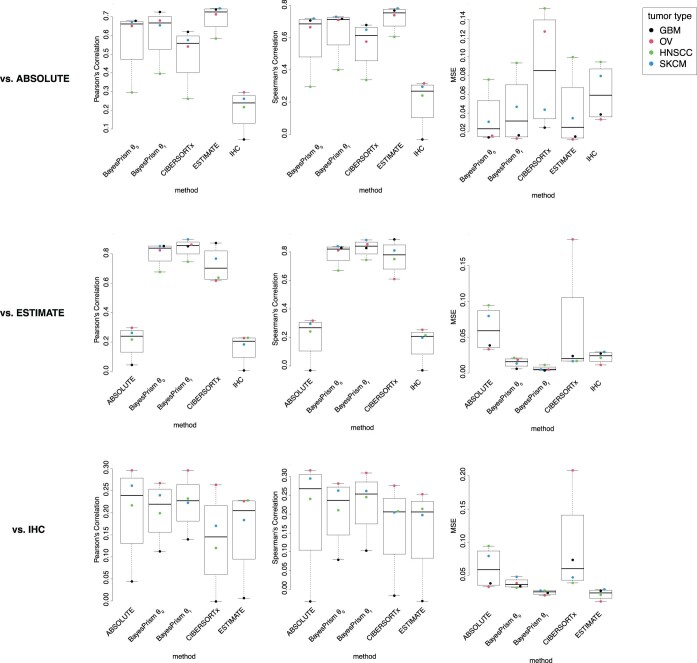
Comparison between tumor purity inferred by BayesPrism, CIBERSORTx, ABSOLUTE, ESTIMATE and IHC. Boxplots show the correlation between malignant fractions inferred by each method for TCGA-GBM, TCGA-OV, TCGA-HNSCC, and TCGA-SKCM. Three statistics were computed: Pearson’s correlation coefficient (left column), Spearman’s correlation coefficient (center column), and mean squared error (right column). Statistics were computed between each method indicated by the x axis and the mean of ABSOLUTE scores from two sources (top row), ESTIMATE (center row), and IHC (bottom row). Boxes mark the 25th percentile (bottom of box), median (central bar), and 75th percentile (top of box). Whiskers represent extreme values within 1.5 fold of the inter quartile range.

**Extended Data Fig. 10:**
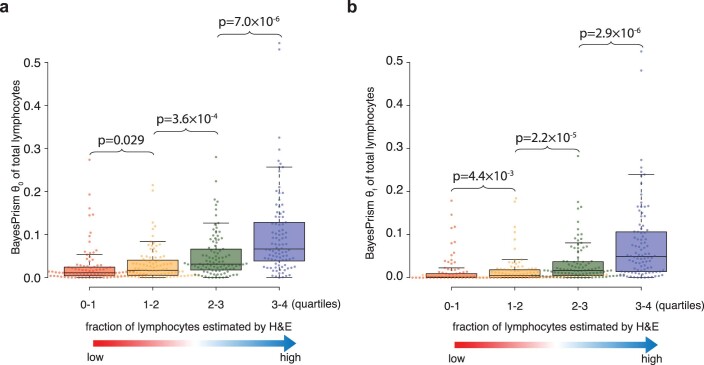
Comparison between the total lymphocyte fraction estimated by BayesPrism and H&E. Box plots show the distribution of θ_0_ (**a**) and θ_f_ (**b**) of total lymphocytes computed by summing across CD4+ and CD8+ T cells B cells and NK cells. θ_0_ (**a**) and θ_f_ are binned by the quantiles of the fraction of H&E patches classified positive for tumor infiltrating lymphocytes (TIL). Boxes mark the 25th percentile (bottom of box), median (central bar), and 75th percentile (top of box). Whiskers represent extreme values within 1.5-fold of the inter quartile range. *P* values were calculated using the one-sided Wilcox test. 379 independent TCGA-SKCM patients with a unique single bulk RNA-seq sample and H&E data available were analyzed. Each bin contains n=95, 95, 95 and 94 samples respectively. Source data

We analyzed the proportion of cell types in 1,142 samples from The Cancer Genome Atlas (TCGA) from three tumor types: GBM, HNSCC and SKCM^16–18^. To maintain the highest possible accuracy, we used a scRNA-seq reference from the same tumor type in each deconvolution task^19–21^. Using these reference datasets provided estimates of six cell types for GBM, ten for HNSCC and eight for SKCM (Fig. 2a). Estimates of tumor purity closely resembled those obtained using copy number variations by ABSOLUTE^22^ and marker gene expression by ESTIMATE^23^ (Extended Data Fig. 9 and Supplementary Fig. 2). Likewise, estimates for the fraction of lymphocytes were correlated with those obtained by counting lymphocyte patches in hematoxylin and eosin sections in the SKCM dataset^24^ (Extended Data Fig. 10). Finally, across large cohorts of tumors, nonmalignant cell types had a rich correlation structure with one another that mirrored several previously described observations in the literature (Supplementary Note 7). These benchmarks support the accuracy of BayesPrism in estimation of cell type fractions in bulk tumor samples.

We asked whether nonmalignant cell types are correlated with patient survival. Samples contributed to TCGA have substantial differences in treatments, genetic drivers and other confounders (Supplementary Table 2). We excluded samples from each tumor type having genetic or clinical covariates (for example, IDH mutant GBMs, metastatic HNSCC and nonmetastatic SKCM) with a large, well-documented effect on prognosis. We examined the association between cell type abundance and survival using two Cox proportional-hazards models by (1) stratifying samples into high and low cell type abundance using the median value, and (2) treating cell type abundance as a continuously valued variable (Methods).

Our analyses revealed several significant associations between immune cell types and clinical outcomes. In SKCM, where CD4^+^ and CD8^+^ cells were annotated separately in the reference scRNA-seq dataset, we found that CD8^+^ T cells had a stronger correlation with survival (HR = 0.498[0.356,0.698]; Fig. 2b and Supplementary Fig. 3), consistent with previous reports^25^. The proportion of T cells was also associated with better clinical outcomes in HNSCC, but the effect was significant using only the model that treated cell type abundance as a continuous variable (*P* = 0.001, Wald test). BayesPrism also revealed significant positive survival associations with B cells and mast cells in HNSCC (HR = 0.694[0.509,0.948] and HR = 0.668[0.49,0.912], respectively).

The prognostic value of macrophages is more controversial than that of other immune cell types. Macrophage estimates by BayesPrism were positively associated with survival in SKCM (HR = 0.648[0.464,0.906]; *P* = 0.01, log-rank test; Fig. 2c). In contrast some recent studies, mostly examining other patient cohorts, have noted either no significant association or the opposite trend^26^. Intriguingly, albeit not statistically significant, we noted that high macrophage infiltration carried a poor prognosis in GBM (HR = 1.18[0.795,1.76]) and no survival association in HNSCC (HR = 1.02[0.749,1.38]). We hypothesized that differences in macrophage cell state may contribute to the differences we observed in survival associations. To test this, we used BayesPrism to estimate macrophage-specific gene expression in samples with >5% macrophage content. We compared macrophage expression with marker genes characteristic of two macrophage subpopulations^27^, M1 and M2, that are believed to have different roles in the TME (Methods). Macrophages from GBM had the highest M2 score and the lowest M1 score, whereas those from SKCM had the lowest M2 score and an M1 score comparably high to that from HNSCC (Fig. 2d). Furthermore, macrophage polarization had an extremely strong association with survival in SKCM (Fig. 2e). In GBM the trend was in a consistent direction (HR = 0.648[0.464,0.906]), although the log-rank test did not show statistical significance (*P* = 0.3; Supplementary Fig. 3). Taken together, these findings highlight the importance of both macrophage content and macrophage cell state in shaping clinical outcomes across different malignancies.

### Gene expression patterns correlated with TME cell types

Prioritizing genes that lie either upstream or downstream of interactions between malignant cells and the TME would be useful for a variety of applications. We developed an approach that uses the correlation between gene expression in malignant cells and the fraction of nonmalignant cell types (hereafter referred to as the ‘query cell type’) across large numbers of bulk samples to identify candidate interacting genes. We found that BayesPrism reduced spurious correlations caused by gene expression in the query cell type (Supplementary Note 8). However, missing cell states from the scRNA-seq reference, a scenario often encountered in a highly heterogeneous TME, could result in transcripts that are highly expressed in a missing nonmalignant cell state to be partially assigned to malignant cells if the scRNA-seq of the malignant cells also shows moderate expression of those genes. This issue may cause potential false-positive correlations between the estimated expression level of that gene and the fraction of the query cell type containing the missing cell states. To further reduce potential false-positive associations, we implemented two additional filters (Supplementary Note 9). First, we devised a likelihood ratio test to test the null hypothesis that gene expression in the query cell type alone explains the variation in query cell fraction. Second, we enriched for genes intrinsic to malignant cells by selecting those expressed at significantly higher levels in at least one malignant cell state compared to all nonmalignant cell types based on the scRNA-seq reference. These filters yielded a conservative set of candidate genes in which malignant cell expression correlated with nonmalignant cell fraction.

**Fig. 3:**
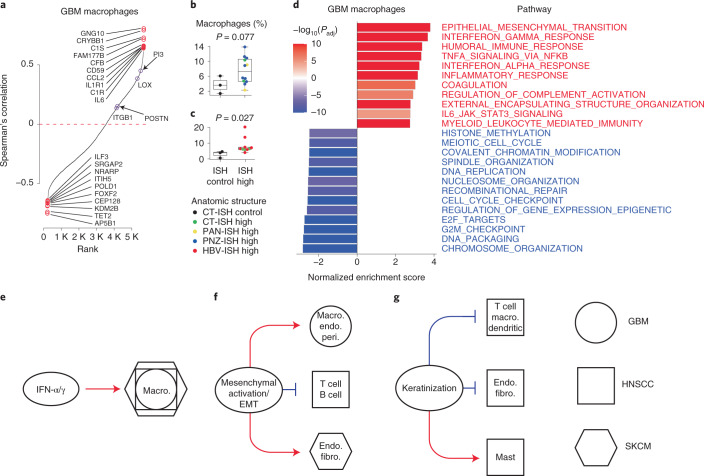
Correlation between malignant cell gene expression and nonmalignant cell fraction. **a**, Rank-ordered plot showing Spearman rank correlation between gene expression in malignant cells inferred by BayesPrism and macrophage fraction in the TCGA-GBM dataset. The top ten positive and negative outlier genes are marked in red; purple circles highlight experimentally validated regulators of macrophage infiltration in GBM, or genes whose expression correlates with macrophage infiltration in IVY GAP. **b**,**c**, Boxplots showing BayesPrism-inferred percentage of macrophage infiltration for regions with low (ISH control) or high (ISH high) expression of two target genes, *PI3* (**b**) and *POSTN* (**c**). Colors indicate anatomic structures associated with ISH experiments. Boxes mark the 25th percentile (bottom), median (central bar) and 75th percentile (top); whiskers represent extreme values within 1.5-fold of interquartile range. Statistical significance was determined by two-sided *t*-test. Sample size of PI3 was *n* = 3 for ISH control and *n* = 12 for ISH high; sample size of POSTN was *n* = 3 for ISH control and *n* = 10 for ISH high, with *n* representing the number of independent patients. Uncorrected *P* values are reported. **d**, Barplot showing normalized gene set enrichment score of genes ranked by correlation with macrophage cell fraction in GBM, as computed in **a**. Only the top 20 semantically nonredundant most enriched biological processes were selected for visualization. Padj, multiple testing-corrected *P* values were determined using the Benjamini–Hochberg method. **e**–**g**, Cartoons summarizing three patterns of relationship between biological processes and infiltration of nonmalignant cell types: IFN-α/γ (**e**), mesenchymal activation/EMT (**f**) and keratinization (**g**). Red arrows and blue flat-headed arrows denote positive and negative correlations, respectively. Shapes represent the tumor types of nonmalignant cells. Macro., macrophage; endo., endothelial cell; peri., pericyte. Source data

We first asked whether we could recover known positive regulators of macrophage infiltration in IDH-wild-type GBM^28,29^. Genes previously reported to have interactions all had statistically significant positive correlations with macrophage infiltration, including *POSTN*, *ITGB1* and *LOX* (Fig. 3a). We also identified numerous other correlations with a stronger magnitude. Putative candidate interacting genes include *GNG10*, *CRYBB1*, *FAM177B*, *CP*, *GLRX* and *PI3*, genes involved in the complement pathway (*C1S*, *CFB*, *CD59* and *C1R*) and cytokine receptors and ligands (*CCL2*, *IL1R1* and *IL6*). To validate new correlations discovered using BayesPrism, we attempted to reproduce them using an independent bulk RNA-seq dataset composed of 148 laser-capture, microdissected regions from 34 GBMs from IVY GAP^30^. We asked whether tumor regions in which malignant cells expressed high levels of candidate genes had higher macrophage infiltration. We used BayesPrism to quantify the fraction of macrophages by deconvolving all 148 IVY GAP samples. Each bulk RNA-seq sample was collected adjacent to sections analyzed by in situ hybridization (ISH) for cancer stem cell markers^30^. Despite limited sample size per marker in the IVY GAP dataset, we observed higher macrophage content in ISH-positive sections of *PI3* and *POSTN*, the only two genes passing our filters that were analyzed by at least ten ISH experiments (Fig. 3b,c, Supplementary Table 3a and Methods). Thus, BayesPrism identified correlations using TCGA that could be reproduced by intratumoral heterogeneity.

We next extended our analysis to identify candidate interactions in GBM, SKCM and HNSCC (Supplementary Fig. 4). To summarize the biological processes associated with cell–cell interactions, we performed gene set enrichment analysis^31^ using correlation coefficients between candidate interacting genes and fractions of nonmalignant cell types (Fig. 3d). Our analyses revealed several interaction patterns. First, many of the biological processes correlated with the fraction of nonmalignant cell types were discovered independently in all three tumor types. For example, interferon-gamma/alpha response was positively correlated with macrophages in all three tumor types (Fig. 3e). Because macrophages secrete interferon-gamma and -alpha upon activation, our finding probably reflects the gene expression response in malignant cells to macrophage infiltration. Second, biological processes were associated with different cell types in different cancer types. Mesenchymal activation or epithelial–mesenchymal transition (EMT) can play a wide variety of roles in the tumor depending on other factors in the TME^32^. Mesenchymal activation was positively associated with both macrophages in GBM and with endothelial cells and fibroblasts in SKCM, and negatively with lymphocytes in HNSCC (Fig. 3d,f). Third, some biological processes were exclusively associated with a single tumor type but with multiple cell types in that tumor. For example, keratinization was negatively associated with multiple nonmalignant cells in HNSCC, except for mast cells which had a positive association (Fig. 3g). One interpretation is that keratinization by malignant cells affects tumor stiffness to exclude certain cell types from the TME. These results highlight how BayesPrism can be used to study interactions between biological processes in malignant and nonmalignant cell infiltration.

### BayesPrism identifies malignant cell-intrinsic gene programs

Evolutionary pressure pushes malignant cells to optimize for different tasks that are essential for their survival, which is done by regulating sets of coexpressed genes known as gene programs^33^. These gene programs provide expression signatures that are characteristic of the heterogeneity between different patients. Existing clustering methods often identify gene programs that reflect differential infiltration of nonmalignant cell types rather than gene expression in malignant cells.

**Fig. 4:**
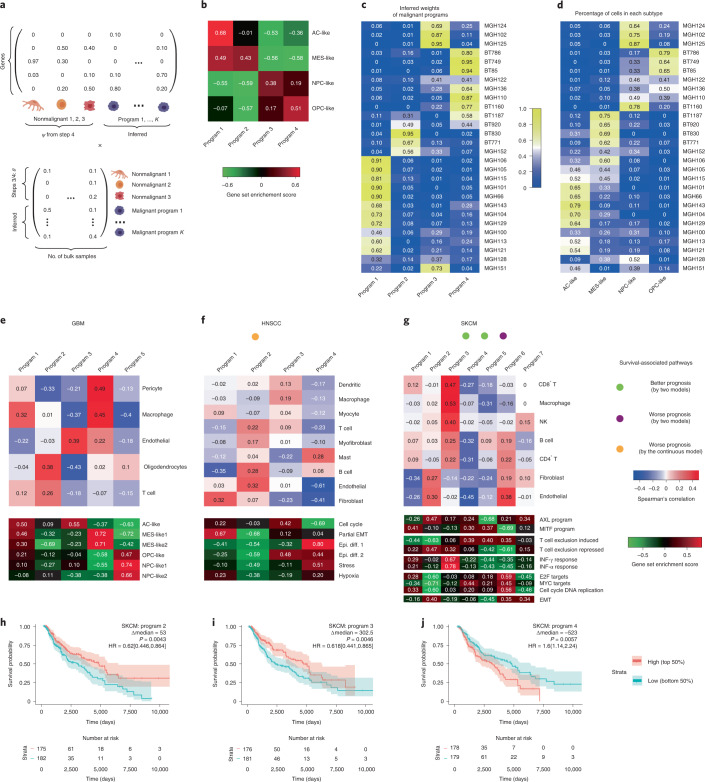
BayesPrism redefines GBM molecular subtypes after excluding expression in nonmalignant cells. **a**, Cartoon illustrating mathematical setup of the embedding learning formulated as a matrix factorization problem. **b**, Heatmap showing gene set enrichment score for each gene program of malignant cells in GBM28 pseudo-bulk inferred by BayesPrism. Marker genes in each cluster reported by Neftel et al.^34^ were used as the gene sets. **c**, Heatmap showing inferred weights of each gene program of malignant cells in GBM28. **d**, Heatmap showing fraction of malignant cells assigned to each cluster in GBM28. **e**–**g**, Top, Spearman’s rank correlation between normalized weights of gene programs and the fraction of nonmalignant cells in three TCGA tumor types: GBM (**e**), HNSCC (**f**) and SKCM (**g**); bottom, gene set enrichment score for selected gene sets. Colored dots indicate whether the normalized weight of a particular gene program was significantly associated with survival (*P* < 0.05), by (1) log-rank test in which the weights were stratified at median (green and purple dots) and (2) Cox proportional-hazards models in which weights were modeled either as a continuous variable (green and purple dots) or by the continuous variable model only (orange dot). **h**–**j**, Kaplan–Meier plots of gene programs significantly associated with patient survival by two models: SKCM programs 2 (**h**), 3 (**i**) and 4 (**j**). *P* values were computed using the log-rank test; hazard ratio was defined by high/low, and the 95th percentile confidence interval is shown in square brackets. Transparent colors denote 95% confidence bands. Epi. diff., epithelial differentiation.

We developed a module in BayesPrism to infer a linear combination of gene programs that best explain expression heterogeneity in bulk RNA-seq data after factoring out gene expression from nonmalignant cell types (Fig. 4a, Methods and Supplementary Note 1). We validated our approach on pseudo-bulk data generated by aggregating scRNA-seq reads across 28 GBMs. BayesPrism recovered gene programs similar to those recently obtained by factorization of 6,863 single malignant cells from the same dataset^34^ (Fig. 4b). The weights of each gene program learned by BayesPrism were correlated with the fraction of cells in each tumor assigned to each of the four major subtypes (Fig. 4c,d). Thus, in this case, BayesPrism learned gene programs from pseudo-bulk data similar to those obtained from single cells.

We applied embedding learning to GBM, HNSCC and SKCM. BayesPrism revealed several programs in GBM that were similar to those in previous studies^34,35^, including program 3 (classical and AC-like), program 4 (mesenchymal) and program 5 (proneural, OPC and NPC-like) (Fig. 4e). In HNSCC, program 1 was enriched for the partial EMT program identified by the single-cell study^19^ (Fig. 4f) and had a negative association with survival (*P* = 0.017, Wald test). In SKCM, we identified multiple survival-associated gene programs that were enriched or depleted for AXL and MITF gene programs (reported previously using TCGA bulk data), as well as a T cell exclusion program (identified in a recent scRNA-seq study; Fig. 4g–j). Gene set enrichment analysis, using either the inferred expression profile of each program or differentially expressed genes between bulk samples associated with each program (Supplementary Tables 4 and 5), provided mechanistic insights into how each program affects clinical outcomes (Supplementary Note 10).

The mesenchymal program in HNSCC and the neural subtype in GBM were both previously proposed to be artifacts caused by the presence of fibroblasts or normal brain tissue, respectively, in bulk RNA-seq data^19,35^. In agreement with this proposal, BayesPrism did not identify any gene programs that were similar to either the mesenchymal subtype in HNSCC or the neural subtype in GBM. Thus, we conclude that the embedding learning module reduced the influence of nonmalignant cell types, resulting in gene programs intrinsic to malignant cells.

### Spatial heterogeneity of gene programs and cell types in GBM

**Fig. 5:**
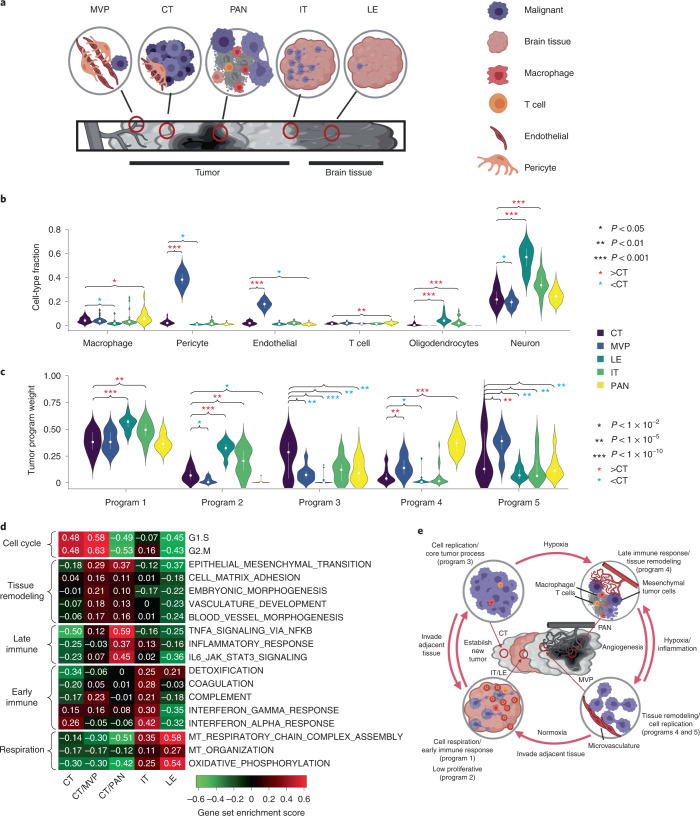
BayesPrism reveals spatial heterogeneity in GBMs. **a**, Cartoon depicting the spatial relationship between anatomic structures present in IVY GAP samples. **b**,**c**, Violin plots showing the distribution of cell type fractions (**b**) and weights of each gene program learned from TCGA-GBM (**c**) in the inferred expression of malignant cells of each anatomic structure over 122 IVY GAP samples. Median fractions are denoted by white dots and upper/lower quartiles by bars. Asterisks denote significant differences between CT and other anatomic structures, based on a linear mixed model. *P* values were computed by two-sided *t*-test using a linear mixed model without correction for multiple testing. Values of test statistics are given in Source data. **d**, Heatmap showing gene set enrichment score of each anatomic structure for biological processes selected from the correlation analysis shown in Fig. 3d and Supplementary Fig. 4a. **e**, Model depicting interaction between gene programs in malignant and nonmalignant cells in the GBM microenvironment. Source data

We hypothesized that the relationship between the activation of gene programs in malignant cells and the proportion of nonmalignant cell types in the microenvironment displays substantial intratumoral spatial heterogeneity. We deconvolved 122 bulk RNA-seq samples microdissected into five structures by IVY GAP^30^: leading edge (LE), infiltrating tumor (IT), cellular tumor (CT), microvascular proliferation (MVP) and pseudo-palisading cells around necrosis (PAN) (Fig. 5a and Supplementary Table 3b). Notably, the TME of these distinct structures is known to differ in several respects, including blood supply, oxygen level and immune stress, all of which probably affect both cell type composition and malignant cell state. Deconvolution was conducted using a reference consisting of the microwell GBM scRNA-seq dataset^21^. Because two structures (LE and IT) contain large amounts of normal brain tissue, we added primary neurons from a separate source^36^. The addition of neurons into the reference was useful in evaluation of the relative quantity of normal brain tissue in each sample. We caution that batch effects between cell types in the reference dataset will tend to systematically overestimate neurons, although the relative rank across samples will be preserved allowing a qualitative comparison among the five structures (Supplementary Note 11).

We examined which cell types and gene programs (identified using TCGA, above) were enriched in the anatomical structures investigated by IVY GAP (Fig. 5b,c). As expected based on the nature of corresponding anatomical structures, MVP regions were highly enriched for endothelial cells and pericytes while LE and IT were enriched for oligodendrocytes and neurons. Notably, PAN regions were enriched for macrophages and T cells. Extending our analysis to gene programs, we found that LE and IT were enriched for programs 1 and 2, CT for program 3, PAN regions for program 4 and MVP for programs 4 and 5.

To help interpret enrichments in the programs obtained by BayesPrism, we next examined gene set enrichment scores in malignant cells (inferred using BayesPrism) within each IVY GAP structure for a subset of biological processes that showed evidence of substantial variation in TCGA-GBM (above; Fig. 5d). We found that CT and MVP were highly proliferative, consistent with their enrichment for programs 3 and 5, which were enriched for cell proliferation terms. MVP and PAN were both enriched for tissue remodeling and immune interactions (program 4), while MVP was more angiogenic and PAN more inflammatory. Both IT and LE were highly respiratory, LE being the most respiratory and the least proliferative, explaining their enrichment for program 1. IT was also enriched for a subset of proinflammatory immune processes, most notably interferon response. Taken together, our analysis shows how BayesPrism was able to link pathways and gene programs with spatial anatomical structures using the IVY GAP dataset.

## Discussion

A large body of literature now provides examples of how nonmalignant cells influence malignant cell function, confirming more than a century of speculation about the critical role of the TME^1^. However, our knowledge remains largely anecdotal and is based mostly on work in animal models rather than human subjects. scRNA-seq has recently made it possible to measure not only cell types present in the tumor but also their gene expression states, in a systematic manner^37^. Although scRNA-seq provides the correct data modality, current studies do not have a sufficiently large sample size to address these questions. In parallel, thousands of bulk RNA-seq datasets are now available that provide weak information about the entire cellular milieu in a variety of malignancies. Here we leveraged both genomic resources by developing a rigorous statistical model to integrate single-cell and bulk RNA-seq data, providing a new lens into this major challenge in oncology.

Our integrative analysis also provides insights into disease progression. Taking GBM as an example, our joint analysis of TCGA cohorts and spatially dissected data prompted us to propose a model that links malignant cell states and nonmalignant cell infiltration to tumor progression (Fig. 5e). As malignant cells grow rapidly they deplete nutrients and may also encounter immune stress, leading to necrosis (Fig. 5e, top right). Consistent with this phase, we observed an enrichment of immune cells and mesenchymal program 4, which showed stronger mesenchymal activation and lower respiratory activity in PAN regions (Fig. 5b,c). Malignant cells may activate these tissue-remodeling pathways to promote M2 macrophage polarization and angiogenesis. As microvascular structure develops, malignant cells proliferate rapidly (Fig. 5e, bottom right), supported by the high cell cycle score in malignant cells near MVP (Fig. 5d). Proliferating cells invade adjacent normal brain tissues, where oxygen supply is ample. As they do so, their major task changes from rapid proliferation to respiration to generate the stores of ATP necessary to synthesize essential molecular machinery (Fig. 5e, bottom left). This finding is based on enrichment of respiration pathways in the LE and IT structures (Fig. 5d). Finally, having accumulated sufficient cellular machinery and as the local oxygen level decreases, malignant cells then resume rapid proliferation. Our model also suggests that the classical-like program 3 may reflect an earlier stage of cancer growth where blood supply is ample. This proposal explains why classical tumors recur as mesenchymal tumors in longitudinal studies more frequently than in the other direction^35^. Taken together, this model illustrates how GBM cells optimize over multiple tasks to reshape and respond to changes in the local microenvironment.

BayesPrism fills several critical needs in the genomics toolbox. BayesPrism more accurately deconvolves bulk RNA-seq into the proportion of cell types than previous approaches, thanks in part to the Bayesian statistical model that models differences between bulk and scRNA-seq data. Most importantly, BayesPrism jointly models cell types and their sample-specific average expression, which is crucial for the analyses reported here. It is important to note that the gene expression and cell type fractions estimated by BayesPrism represent a mathematically optimal solution given the information from the scRNA-seq reference. In practice the accuracy of BayesPrism can be affected by missing cell states in the reference matrix, which is a general issue for all deconvolution algorithms. The expression of missing cell states in a heterogeneous TME can sometimes deviate from the prior distribution modeled by BayesPrism, resulting in partial assignment of transcripts from missing cell states to cell states belonging to other cell types. Caution needs to be taken when performing correlation analysis between the posterior estimates of gene expression and cell type fraction, potentially using similar filters to those introduced here. Nevertheless, we speculate that deconvolution of tumor samples will become more accurate as we collect single-cell data from more patients, each presumably covering nuances in the transcriptional state. Thus, we envision that BayesPrism will provide a new type of lens for integration of the ever-growing amount of scRNA-seq data with existing large cohorts of bulk RNA-seq data, allowing insights into tumor–microenvironment interactions.

## Methods

### Overview of BayesPrism

A complete mathematical description and justification of BayesPrism is included in Supplementary Note 1. Here we provide a summary of BayesPrism and its use in this manuscript. The R package of BayesPrism can be downloaded at https://github.com/Danko-Lab/BayesPrism.git; the BayesPrism web portal can be accessed at https://dreg.dnasequence.org.

BayesPrism is comprised of two functional modules: (1) a module that infers the cell type fraction and gene expression of each cell type in each bulk RNA-seq sample (Fig. 1a), and (2) a module designed to identify commonly occurring malignant gene programs after removal of gene expression in nonmalignant cells infiltrating the tumor (Fig. 4a). The second module depends on the output of the deconvolution module.

#### Definition of cell types and cell states

BayesPrism models gene expression of each cell type using a multinomial distribution. However, depending on the granularity of the cell type labels provided by the user, gene expression can be heterogeneous within each cell type and hence show overdispersion from the multinomial distribution. This can be particularly the case for malignant and nonmalignant cells, such as M1/M2 macrophages, in the TME, or for differences in cell cycle or copy number variation in malignant cells. To better accommodate heterogeneity in cell states we use the concept of cell states (or cell subtypes), which can be obtained by further subclustering within each heterogeneous cell type. BayesPrism computes the posterior sum over the cell states to obtain the statistics for each cell type.

The following notation will be used upon describing the raw input:

Shared between bulk and scRNA-seq:

- *G*, number of genes

Bulk:

- *N*, number of bulk samples
- *X*_*ng*_, raw count of the *g*th gene in the *n*th bulk sample
- *R*_*n*_, total reads of the *n*th bulk sample over *G* genes

scRNA-seq:

- *S*, number of cell states
- *T*, number of cell types
- *C*, number of cells
- *S*_*c*_, the cell state of the *c*th cell
- *T*_*c*_, the cell type of the *c*th cell
- *W*_*cg*_, raw count (UMI) of the *g*th gene in the *c*th cell
- *R*_*c*_, total reads (UMIs) of the *c*th cell across *G* genes

#### Constructing the scRNA-seq reference *φ*

We assume that the raw count of each cell conditional on the cell state follows the multinomial distribution, where $\varphi \in {\Bbb R}^{S \times G}$ encodes the event probabilities of the *g*th gene in the *s*th cell state.

$$W_{c \cdot }|S_c\sim {\mathrm{multinomial}}(\varphi _{s_c} \cdot R_c),$$

where

1 $$\mathop {\sum}\limits_{g = 1}^G {\varphi _{s_cg}} = 1,\,{\mathrm{for}}\,\forall S_c \in \{ 1,...,S\}$$

Hence, the maximum likelihood estimate of $\varphi _{sg}$ is obtained by summing read count for cells from the *s*th cell state and then renormalizing such that the sum across genes is 1:

2 $$\widehat {\varphi _{sg}} = \frac{{\mathop {\sum}\nolimits_{c \in \{ c:S_c = s\} } {W_{cg}} }}{{\mathop {\sum}\nolimits_{g \in \{ 1,...,G\} } {\mathop {\sum}\nolimits_{c \in \{ c:S_c = s\} } {W_{cg}} } }}$$

To avoid zero entries in $\widehat {\varphi _{sg}}$, we add a pseudo-count to all genes such that after renormalization to 1, the zero entries are equal to the user-defined pseudo.min (set to 10^–8^ by default). This is done by the norm.to.one function.

#### Inferring cell state fraction and gene expression

The following notation will be used to describe this step:

- *μ*_*ns*_, estimates of fraction of reads from the *s*th cell state and the *n*th bulk sample
- *R*_*ns*_, total reads assigned to the *s*th cell state in the *n*th bulk sample
- *U*_*nsg*_, number of reads assigned to the *g*th gene of *s*th cell state in the *n*th bulk sample
- *α*, the hyperparameter of Dirichlet distribution, set to a small value (10^–8^) to represent a symmetric noninformative and weak prior by default.

We assume that reads from the *n*th bulk sample are generated from the following process:

3 $$\mu _{n \cdot }\sim {\mathrm{Dirichlet}}(\alpha )$$

4 $$R_{n \cdot }\sim {\mathrm{multinomial}}(\mu _{n.},R_n)$$

5 $$U_{ns \cdot }\sim {\mathrm{multinomial}}(\varphi _{s \cdot },R_{ns})$$

6 $$X_{ng} = \mathop {\sum}\limits_{s = 1}^S {U_{nsg}}$$

Using Gibbs sampling, we sample from the joint posterior distribution (Supplementary Note 1 shows the full derivation).

7 $$P(U_n,\mu _n|X_n,\varphi ;\alpha ),$$

and simultaneously their marginals:

8 $$P(U_n|X_n,\varphi ;\alpha )$$

and

9 $$P(\mu _n|X_n,\varphi ;\alpha ).$$

We then compute the posterior means for *U*_*n*_ and *μ*_*n*_, which we denote as $\overline {U_n}$ and $\overline {\mu _n}$, respectively.

#### Inferring cell type fraction and gene expression

Because cell state is often used to approximate gene expression on a continuous manifold, it may not necessarily correspond to any actual state in the bulk. Therefore, we compute the posterior sum of the fraction and expression of each cell type over multiple cell states within a given cell type. The posterior sum is also of lower variance and more biologically interpretable than that of individual cell states. The following notation will be used to describe this step:

- $\theta _{0_{nt}}$, estimates of fraction of reads from the *t*th cell type and the *n*th bulk sample
- *Z*_*ntg*_, number of reads assigned to the *g*th gene of *t*th cell type in the *n*th bulk sample.

More formally,

10 $$\theta _{0_{nt}} = \mathop {\sum }\limits_{s \in \{ s:h(s) = t\} } \overline {\mu _{ns}}$$

11 $$Z_{ntg} = \mathop {\sum }\limits_{s \in \{ s:h(s) = t\} } \overline {U_{nsg}} ,$$

where *h* is a surjective function that maps the cell state index to the cell type index—that is, $h:\{ 1,...,S\} \to \{ 1,...,T\}$.

#### Updating cell type fraction estimates

Under situations where (1) nonmalignant cell types are of low heterogeneity across bulk samples and (2) they are presented at substantial fractions—for example, >1%—in at least one bulk sample, it is beneficial to leverage the information shared across bulks to improve the estimates of cell type fractions. This is done by first constructing an updated reference matrix *ψ* to replace *φ*, and then re-estimating the posteriors of cell type fraction using *ψ*. The rationales for modeling *ψ* are described below.

For malignant cells, BayesPrism infers a maximum likelihood estimate to infer a sample-specific *ψ*, without pooling information across bulk samples. This feature allows users to estimate gene expression in the cell type that exhibits substantial heterogeneity. The following notation will be used to describe this step:

- $\psi _{{\mathrm{mal}}_{ng}}$, the updated reference of the *g*th gene of the malignant cell from the *n*th bulk
- $\psi _{{\mathrm{env}}_{tg}}$, the updated reference of the *g*th gene of the *t*th nonmalignant cell
- *σ*, a hyperparameter describing a weak prior on *ψ*_env_, set to 2 by default to represent a weak symmetric prior
- $\theta _{f_{nt}}$, the updated cell type fractions of the *t*th cell type in the *n*th bulk sample
- $R_{nt} = \mathop {\sum}\nolimits_{g = 1}^G {Z_{ntg}}$, total reads assigned to the *t*th cell type in the *n*th bulk sample
- $\varphi' _{tg} = \frac{{\mathop {\sum}\nolimits_{c \in \{ c:T_c = t\} } {W_{cg}} }}{{\mathop {\sum}\nolimits_{g \in \{ 1,...,G\} } {\mathop {\sum}\nolimits_{c \in \{ c:T_c = t\} } {W_{cg}} } }}$, the reference matrix defined for each cell type *t*, similar to equation (2)

To construct *ψ*_mal_, we assume that

12 $$Z_{nt \cdot }\sim {{{\mathrm{multinomial}}}}(\psi _{mal_{n \cdot }},R_{nt}),{{{\mathrm{where}}}}\,t = {\mathrm{malignant}},$$

and hence the maximum likelihood estimate of *ψ*_mal_ is:

13 $$\widehat {\psi _{{\mathrm{mal}}_{ng}}} = \frac{{Z_{ntg}}}{{\mathop {\sum }\nolimits_{g = 1}^G Z_{ntg}}},{\mathrm{where}}\,t = {\mathrm{malignant}}.$$

Finally, we adjust $\widehat {\psi _{{\mathrm{mal}}}}$ by adding a pseudo-count similar to equation (2).

To estimate *ψ* for nonmalignant cells in the TME, denoted by *ψ*_env_, BayesPrism pools information across all bulk samples to estimate cell-type-specific expression. We assume that, for nonmalignant cell types, gene expression is reasonably similar across bulk samples^15,20,21^ and in these cases it is appropriate to share information between samples by estimating *ψ* using all bulk data. Considering that some nonmalignant cells may be present in extremely low amounts in all bulk samples, we put a prior on *ψ*_env_ and derive a maximum a posterior (MAP) estimator, such that the estimates of *ψ*_env_ will be close to *φ*_env_ when there is little information from the bulk. More specifically, we model a generative process as follows:

14 $$\log (\gamma _{tg})\sim {\mathrm{normal}}(0,\sigma )$$

15 $$\psi _{{\mathrm{env}}_{tg}} = \frac{{\varphi' _{tg} \cdot \gamma _{tg}}}{{\mathop {\sum }\nolimits_{g = 1}^G \varphi' _{tg} \cdot \gamma _{tg}}}$$

16 $$Z_{nt \cdot }\sim {{{\mathrm{multinomial}}}}(\psi _{{\mathrm{env}}_{t \cdot }},R_{nt})\,,{{{\mathrm{for}}}}\,t\, \in \{ {\mathrm{malignant}}\} ^c$$

Because there is no closed form solution to *ψ*_env_, we use numerical optimization to obtain the MAP of $\widehat {\psi _{{\mathrm{env}}_{tg}}}$. For the *n*th bulk sample, we construct a sample-specific reference *φ*_*ntg*_ by concatenating $\widehat {\psi _{{\mathrm{env}}_{tg}}}$ and the *n*th row of $\widehat {\psi _{{\mathrm{mal}}_{ng}}}$. More formally,

17 $$\psi _{ntg} = \widehat {\psi _{{\mathrm{mal}}_{ng}}},{\mathrm{for}}\,t = {\mathrm{malignant}},$$

and

18 $$\psi _{ntg} = \widehat {\psi _{{\mathrm{env}}_{tg}}},{\mathrm{for}}\,t \in \{ {\mathrm{malignant}}\} ^c.$$

We then use the same generative process as described in equations (3)–(6) by replacing *φ*′ with the sample-specific reference *ψ*, and derive the posterior similar to equation (9):

19 $$P(\theta _{f_{n.}}|X_n,\psi _{n..};\alpha ).$$

#### The embedding learning module

The second module of BayesPrism was designed to identify gene expression patterns that arise commonly among bulk RNA-seq samples after removal of nonmalignant cells infiltrating the tumor. BayesPrism learns the latent embeddings, called malignant bases (denoted by *η*), chosen such that their linear combination best approximates gene expression levels in malignant cells. Essentially this module tries to solve the non-negative matrix factorization (NMF) problem:

20 $$\left[ {\begin{array}{*{20}{c}} {X_{1,1}} & \cdots & {X_{1,G}} \\ \vdots & \ddots & \vdots \\ {X_{N,1}} & \cdots & {X_{N,G}} \end{array}} \right]\approx \left[ {\begin{array}{*{20}{c}} {\omega _{1,1}} & {} & {\omega _{1,K}} & {\theta _{1,1}} & {} & {\theta _{N,T - 1}} \\ {} & \ddots & {} & {} & \ddots & {} \\ {\omega _{N,1}} & {} & {\omega _{N,K}} & {\theta _{N,1}} & {} & {\theta _{N,T - 1}} \end{array}} \right] \cdot \left[ {\begin{array}{*{20}{c}} {\eta _{1,1}} & {} & {\eta _{1,G}} \\ {} & \ddots & {} \\ {\eta _{K,1}} & {} & {\eta _{K,G}} \\ {\psi _{{\mathrm{env}}_{1,1}}} & {} & {\psi _{{\mathrm{env}}_{1,G}}} \\ {} & \ddots & {} \\ {\psi _{{\mathrm{env}}_{T - 1,1}}} & {} & {\psi _{{\mathrm{env}}_{T - 1,G}}} \end{array}} \right],$$

written in short as *X*≈ $\upsilon \cdot \zeta$, with $\upsilon \in {\Bbb R}^{N \times M}$ and $\zeta \in {\Bbb R}^{M \times G}$, where *M* = *K* + *T* − 1, with *K* being the number of malignant bases and *T* the number of cell types. In equation (20), *θ* and *ψ*_env_ blocks are the nonmalignant components from *θ* and *ψ*, respectively, inferred by the deconvolution module in equations (19) and (18), and are fixed during inference. *ω* and *η* are latent variables inferred. *η* has a weak prior over *η*_0_:

${\mathrm{log}}(\lambda _{kg})$ ~normal (0, *σ*), where *σ* is set to 2 by default, similar to equation (14), and

$$\eta _{kg} = \frac{{\eta _{0_{kg}} \cdot \lambda _{kg}}}{{\mathop {\sum }\nolimits_{g = 1}^G \eta _{0_{kg}} \cdot \lambda _{kg}}},$$

*η*_0_ represents a prior guess of gene programs of malignant cells. It can be supplied by users either based on domain knowledge or by running clustering or NMF on the expression inferred for malignant cells in equation (13). In addition, *ω* has a weak scaled noninformative Dirichlet prior:

$\kappa _{n \cdot }$ ~ Dirichlet (*α*), where *α* is set to a small value (10^–8^), similar to equation (3).

$$\omega _{n \cdot } = \tau _n \cdot \kappa _{n \cdot },{\mathrm{where}}\,\tau _n = 1 - \mathop {\sum}\limits_{t \in \{ {\mathrm{malignant}}\} ^c} {\theta _{nt}}$$

The observed read count of bulk samples, *X*, is then generated as follows:

$$R_n^\prime$$~ multinomial ($$\upsilon _{n \cdot }$$*, R*_*n*_)

$$V_{nm \cdot }$$ ~ multinomial ($$\zeta _{m \cdot }$$, $$R_{nm}^\prime$$)

*X*_*ng*_ = $$\mathop {\sum}\nolimits_{m = 1}^M {V_{nmg}}$$

*η* is inferred using the expectation-maximization (EM) algorithm to optimize the log posterior to obtain the MAP of *η* while marginalizing *V* and *ω*:

$$\hat \eta _{{\mathrm{MAP}}} = \arg \max _\eta P(\eta |X,\eta _0,\theta ,\psi _{{\mathrm{env}}};\alpha ,\sigma ).$$

*ω* is then taken as the posterior mean at the $\hat \eta _{{\mathrm{MAP}}}$:

$${\hat{\omega}} = {\Bbb E}[\omega |X,\theta ,\psi _{{\mathrm{env}}},\hat \eta _{{\mathrm{MAP}}};\alpha ].$$

### Deconvolution of bulk RNA-seq using BayesPrism

#### Generation of reference expression profiles from scRNA-seq data

We used reference expression profiles generated from scRNA-seq data to deconvolve the bulk RNA-seq data of the corresponding tumor type. We summed raw read counts whenever count data were available^20,21^. For instances where only TPM normalized data were available the scRNA-seq reference for HNSCC (scHNSCC), we summed TPM normalized reads. To generate reference profiles of the cell states of nonmalignant cells, we first reclustered cell types showing substantial intertumoral heterogeneity based on the original scRNA-seq studies, including macrophages in GBM, fibroblasts and T cells in HNSCC and macrophages, B cells and CD4 and CD8 T cells in SKCM. To recluster, we normalized the raw reads of each cell using size factors estimated by scran^38^, then transformed the data using log_2_(*Y* + 0.1) for refGBM8 and log_2_(*Y* + 1) for the scRNA-seq reference for SKCM (scSKCM) and scHNSCC, with *Y* being the count normalized by scran. We then removed ribosomal protein-coding genes and genes from chrM, chrX and chrY. Next, we performed dimensionality reduction using the randomized singular-value decomposition function provided by the rsvd package^39^ at *k* = 30, and clustered the data on the reduced dimension using PhenoGraph with default parameters^40^. To account for heterogeneity in malignant cells, we used subclusters of malignant cells generated by PhenoGraph^40^ in each individual patient, whenever malignant cells were clustered by the author (refGBM8, 60 subclusters in total for eight patients). For datasets where malignant cells were not clustered by the original paper (scHNSCC and scSKCM), we defined malignant cell states using patient ID. Last, we aggregated read counts in each cell state to generate the reference profile *φ*. We found that the expression of many noncoding genes in TCGA was close to zero across all patients, and hence we performed the inference on protein-coding genes when deconvolving TCGA data to speed up downstream analysis. Deconvolution over all genes generated almost identical results (data not shown). In addition, genes on the sex chromosomes were also excluded in the reference to avoid sex-specific transcription states, and ribosomal protein-coding and mitochondrial genes were removed to reduce batch effects. Outlier genes in the bulk, defined as those with expression >1% of total reads in >10% of bulk samples, were excluded from deconvolution analysis.

#### Choice of hyperparameters and retrieval of output from BayesPrism

We used the default hyperparameters of BayesPrism to perform deconvolution: *σ* = 2, *α* =10^–8^. We used the default setting for Gibbs sampling as follows: chain.length = 1,000, burn.in = 500 and thinning = 2 (that is, we ran a Markov chain Monte Carlo of 1,000 samples, discarded the first 500 and used every other sample to estimate parameters of interest). The maximum number of iterations of the conjugate gradient method was set to 10^5^. All cell type fractions used were the initial cell type fraction estimate (*θ*_0_). The updated cell type fraction estimates, *θ*_*f*_, highly correlates with *θ*_0_ (*R* > 0.98), and results from all downstream analyses were consistent whether using *θ*_0_ or *θ*_*f*_.

Details of benchmarks are listed in Supplementary Note 3.

### Embedding learning analysis

To initialize the malignant gene programs (bases), we used the NMF R package^41^ to learn a linear combination that best approximates the normalized expression of malignant cells inferred by the deconvolution module of BayesPrism (res$first.gibbs.res$Zkg.tum.norm). We optimized the number of malignant bases from two to 12, and chose the number of gene programs (*K*) that yielded the best cophenetic score before a significant drop began. This strategy selected *K* = 5 for GBM, *K* = 4 for HNSCC and *K* = 7 for SKCM. We then fixed *K*, randomly initialized NMF 200 times and chose bases that yielded minimal residuals. The optimal bases were then used as the prior for the embedding learning module of BayesPrism, thereby incorporating information from the scRNA-seq reference into the embeddings learned by BayesPrism. Although BayesPrism does not necessarily require the use of input bases learned using external algorithms such as NMF, and can initialize bases using clustering methods when no user-defined input is used, we found that initialization of bases using NMF significantly speeded up the convergence of EM and also facilitated the selection of *K* when no prior information was provided.

### Gene set enrichment analysis

To visualize the magnitude of enrichment of selected gene sets in the inferred embeddings in Fig. 4b,e–g, and the mean of inferred expression of malignant cells in each anatomical structure in Fig. 5d, we calculated gene set variation score using the GSVA R package^42^. To compute the statistical significance of gene set enrichment for correlation coefficients in Fig. 3d, and Wald statistics of differential gene expression in Supplementary Table 4, we performed gene set enrichment analysis using the fgsea R package^31^.

Input gene sets are listed as follows. In Fig. 4b,e–g we used the marker genes of each subtype; in Figs. 3d and 5d and Supplementary Table 4 we used Hallmark v.7.4 (ref. ^43^) and the GO biological process v.7.4 (ref. ^44^) gene sets from The Molecular Signatures Database v.7.4 (ref. ^45^).

Details of fgsea analysis used to generate Supplementary Table 4 are as follows. To prepare for the input of differential expression analysis, we first ranked samples by normalized weights of gene programs in each sample (weights were normalized to sum to 1). We selected the top *N*/*K* samples, with *N* being the number of bulk samples and *K* the number of pathways. Core tumor samples representing each gene program were selected by focusing on samples uniquely selected by a single program. We performed differential expression analysis by comparing the inferred expression profile of malignant cells between the core samples of each gene program of interest with remaining core samples, using DESeq2 (ref. ^46^). The results of differentially expressed genes are shown in Supplementary Table 5. Wald test statistics were used as the input for fgsea.

### Analysis of M1/M2 macrophage score across three tumor types

We first selected tumor samples with inferred macrophage fractions (by BayesPrism) >5%: 127 GBM, 26 HNSCC and 225 SKCM samples were selected. We then applied DESeq2 variance-stabilizing transformation^46^ over the inferred macrophage gene expression profiles. We computed Pearson correlation coefficient between the inferred macrophage expression and the log_2_ transformed expression profile of M1 and M2 macrophages from LM22 over a set of M1/M2 marker genes. Marker genes were defined as those with the highest expression in M1/M2 macrophages across all cell types included by the LM22 reference matrix^27^.

For survival analysis, we focused our analysis on SKCM (Fig. 2e) and GBM (Supplementary Fig. 3d) where >50 samples had sufficient macrophage content to estimate expression. Patients were stratified into two groups: the group with high M1 and low M2 scores versus that with low M1 and high M2 scores, where high and low were defined by comparison to the median of M1/M2 scores.

### Analysis of anatomically resolved transcriptomics data from IVY GAP

Anonymized BAM files for each sample were downloaded from glioblastoma.alleninstitute.org, and raw counts for each gene were obtained using featureCounts^47^ using the GENCODE annotation v24lift37.

To test the statistical significance in the mean of cell type fractions (Fig. 5b) and gene program weights (Fig. 5c; obtained by applying BayesPrism to deconvolve the inferred expression of malignant cells using the pathway profile inferred from TCGA as the reference) across multiple anatomic structures, while taking account of the multiple biological replicates of each patient, we fit a linear mixed model using the lme function from the R package nlme^48^ with a random intercept. We modeled anatomic structures as the fixed effects and patient IDs as random effects. The ground level was set to CT. We maximized the log-likelihood function by setting the method as ‘ML’, and used ‘optim’ as the optimizer.

To validate candidate genes from the correlation analysis in Fig. 3a, we selected genes profiled in at least ten samples from the cancer stem cell RNA-seq study of IVY GAP (Supplementary Table 3a), resulting in the retention of five genes. Two genes out of five, *PI3* and *POSTN*, also passed the filters used to define malignant intrinsic correlative genes and are characterized in Fig. 3b,c. The *P* value of the regress-out filter for gene *POSTN* is 0.02. Although it did not pass the alpha value we used (0.01), we still included it for visualization and validation purposes.

### Survival analysis

To avoid known clinical or genetic factors that have a strong influence on patient survival from confounding our survival analysis, we focused on the population with the greatest sample size after conditional on known confounders. These included primary IDH-1 wild-type tumors for GBM, primary HNSCC and metastatic SKCM. We also attempted to control for human papillomavirus (HPV) state in HNSCC. Although only 72 of 500 samples were annotated for HPV, we nevertheless reproduced consistent trends in a small cohort of 56 patients that were HPV negative (Supplementary Fig. 3b). Two methods were used to test the association between survival and the feature of interest: (1) patients were stratified into high and low groups based on the median value of the feature of interest—for example, normalized weights of malignant gene programs or nonmalignant cell fractions—and then HR was computed by fitting a Cox proportional-hazards regression model for a categorical variable denoting patient groups. (2) Features of interest were modeled as a continuous variable by the Cox proportional-hazards model, in which we derived statistical significance using the Wald test and examined proportional-hazards assumption using the chi-squared test for scaled Schoenfeld residuals—that is, whether the Schoenfeld residuals were independent of time.

### Statistics and reproducibility

No statistical method was used to predetermine sample size. No data were excluded from the analyses. The experiments were not randomized. The investigators were not blinded to allocation during experiments and outcome assessment. No new experiments were conducted for this study. BayesPrism yielded near-identical results among different seeds used by the random number generator.

### Reporting Summary

Further information on research design is available in the Nature Research Reporting Summary linked to this article.

## Supplementary information

Supplementary Information Supplementary Notes 1–11 and figures.

Reporting Summary

Supplementary Tables Supplementary Tables 1–5.

## Data Availability

All datasets used in this study are publicly available. Accession codes used here include GSE103224, GSE131928, GSE115978, GSE103322, GSE146026, GSE132044 and GSE67835. Additional data were downloaded from TCGA (https://portal.gdc.cancer.gov), IVY GAP (https://glioblastoma.alleninstitute.org) and the CIBERSORT website (https://cibersortx.stanford.edu/download.php). Source data are provided with this paper. Data sources for each figure are shown in Supplementary Table 1b. All intermediate data supporting the findings of this study are available from the corresponding author on reasonable request.

## Source data

Source Data Fig. 1 Statistical Source data.
Source Data Fig. 2 Statistical Source data.
Source Data Fig. 3 Statistical Source data.
Source Data Fig. 5 Statistical Source data.
Source Data Extended Data Fig. 3 Statistical Source data.
Source Data Extended Data Fig. 10 Statistical Source data.

## Code availability

BayesPrism and all script files used in the analysis in this manuscript can be downloaded from GitHub at https://github.com/Danko-Lab/BayesPrism.git.

## References

[1] Paget S (1889). The distribution of secondary growths in cancer of the breast. The Lancet.

[2] Greene HS, Harvey EK (1964). The relationship between the dissemination of tumor cells and the distribution of metastases. Cancer Res..

[3] Crawford Y (2009). PDGF-C mediates the angiogenic and tumorigenic properties of fibroblasts associated with tumors refractory to anti-VEGF treatment. Cancer Cell.

[4] Murgai M (2017). KLF4-dependent perivascular cell plasticity mediates pre-metastatic niche formation and metastasis. Nat. Med..

[5] Paiva AE (2018). Pericytes in the premetastatic niche. Cancer Res..

[6] Richters A, Kaspersky CL (1975). Surface immunoglobulin positive lymphocytes in human breast cancer tissue and homolateral axillary lymph nodes. Cancer.

[7] Li B (2016). Comprehensive analyses of tumor immunity: implications for cancer immunotherapy. Genome Biol..

[8] Ostermann E (2008). Effective immunoconjugate therapy in cancer models targeting a serine protease of tumor fibroblasts. Clin. Cancer Res..

[9] Denisenko E (2020). Systematic assessment of tissue dissociation and storage biases in single-cell and single-nucleus RNA-seq workflows. Genome Biol..

[10] Jew B (2020). Accurate estimation of cell composition in bulk expression through robust integration of single-cell information. Nat. Commun..

[11] Baron M (2016). A single-cell transcriptomic map of the human and mouse pancreas reveals inter- and intra-cell population structure. Cell Syst..

[12] Newman AM (2019). Determining cell type abundance and expression from bulk tissues with digital cytometry. Nat. Biotechnol..

[13] Wang X, Park J, Susztak K, Zhang NR, Li M (2019). Bulk tissue cell type deconvolution with multi-subject single-cell expression reference. Nat. Commun..

[14] Dong M (2021). SCDC: bulk gene expression deconvolution by multiple single-cell RNA sequencing references. Brief. Bioinform..

[15] Tirosh I (2016). Dissecting the multicellular ecosystem of metastatic melanoma by single-cell RNA-seq. Science.

[16] Brennan CW (2013). The somatic genomic landscape of glioblastoma. Cell.

[17] Cancer Genome Atlas Network. (2015). Genomic classification of cutaneous melanoma. Cell.

[18] Cancer Genome Atlas Network. (2015). Comprehensive genomic characterization of head and neck squamous cell carcinomas. Nature.

[19] Puram SV (2017). Single-cell transcriptomic analysis of primary and metastatic tumor ecosystems in head and neck cancer. Cell.

[20] Jerby-Arnon L (2018). A cancer cell program promotes T cell exclusion and resistance to checkpoint blockade. Cell.

[21] Yuan, J. et al. Single-cell transcriptome analysis of lineage diversity in high-grade glioma. *Genome Med.* **10**, 57 (2018). 10.1186/s13073-018-0567-9 PMC6058390 30041684

[22] Carter SL (2012). Absolute quantification of somatic DNA alterations in human cancer. Nat. Biotechnol..

[23] Yoshihara K (2013). Inferring tumour purity and stromal and immune cell admixture from expression data. Nat. Commun..

[24] Saltz J (2018). Spatial organization and molecular correlation of tumor-infiltrating lymphocytes using deep learning on pathology images. Cell Rep..

[25] Piras F (2005). The predictive value of CD8, CD4, CD68, and human leukocyte antigen-D-related cells in the prognosis of cutaneous malignant melanoma with vertical growth phase. Cancer.

[26] Falleni M (2017). M1 and M2 macrophages’ clinicopathological significance in cutaneous melanoma. Melanoma Res..

[27] Newman AM (2015). Robust enumeration of cell subsets from tissue expression profiles. Nat. Methods.

[28] Zhou W (2015). Periostin secreted by glioblastoma stem cells recruits M2 tumour-associated macrophages and promotes malignant growth. Nat. Cell Biol..

[29] Chen P (2019). Symbiotic macrophage-glioma cell interactions reveal synthetic lethality in PTEN-null glioma. Cancer Cell.

[30] Puchalski RB (2018). An anatomic transcriptional atlas of human glioblastoma. Science.

[31] Korotkevich, G. et al. Fast gene set enrichment analysis. Preprint at *bioRxiv*10.1101/060012 (2021).

[32] Dongre A, Weinberg RA (2018). New insights into the mechanisms of epithelial–mesenchymal transition and implications for cancer. Nat. Rev. Mol. Cell Biol..

[33] Hart, Y. et al. Inferring biological tasks using Pareto analysis of high-dimensional data. *Nat. Methods* **12**, 233–235 (2015). 10.1038/nmeth.3254 25622107

[34] Neftel C (2019). An integrative model of cellular states, plasticity, and genetics for glioblastoma. Cell.

[35] Wang Q (2017). Tumor evolution of glioma-intrinsic gene expression subtypes associates with immunological changes in the microenvironment. Cancer Cell.

[36] Darmanis S (2015). A survey of human brain transcriptome diversity at the single cell level. Proc. Natl Acad. Sci. USA.

[37] Suvà ML, Tirosh I (2019). Single-cell RNA sequencing in cancer: lessons learned and emerging challenges. Mol. Cell.

[38] Lun ATL, McCarthy DJ, Marioni JC (2016). A step-by-step workflow for low-level analysis of single-cell RNA-seq data with Bioconductor. F1000Res..

[39] Erichson NB, Voronin S, Brunton SL, Kutz JN (2019). Randomized matrix decompositions using R. J. Stat. Softw..

[40] Levine JH (2015). Data-driven phenotypic dissection of AML reveals progenitor-like cells that correlate with prognosis. Cell.

[41] Gaujoux R, Seoighe C (2010). A flexible R package for nonnegative matrix factorization. BMC Bioinform..

[42] Hänzelmann S, Castelo R, Guinney J (2013). GSVA: gene set variation analysis for microarray and RNA-seq data. BMC Bioinform..

[43] Liberzon A (2015). The Molecular Signatures Database (MSigDB) hallmark gene set collection. Cell Syst..

[44] The Gene Ontology Consortium. The Gene Ontology Resource: 20 years and still GOing strong. *Nucleic Acids Res*. **47**, D330–D338 (2019). 10.1093/nar/gky1055 PMC6323945 30395331

[45] Subramanian A (2005). Gene set enrichment analysis: a knowledge-based approach for interpreting genome-wide expression profiles. Proc. Natl Acad. Sci. USA.

[46] Love MI, Huber W, Anders S (2014). Moderated estimation of fold change and dispersion for RNA-seq data with DESeq2. Genome Biol..

[47] Liao Y, Smyth GK, Shi W (2014). featureCounts: an efficient general purpose program for assigning sequence reads to genomic features. Bioinformatics.

[48] Pinheiro, J., Bates, D., DebRoy, S., Sarkar, D. & R Core Team. nlme: Linear and nonlinear mixed effects models. v3.1-141. https://cran.r-project.org/web/packages/nlme/nlme.pdf (2022).

