# Supplementary material for: Cell type and gene expression deconvolution with BayesPrism enables Bayesian integrative analysis across bulk and single-cell RNA sequencing in oncology
Source: Nat Cancer. 2022 Apr 25;3(4):505–17. doi: 10.1038/s43018-022-00356-3 (PMC9046084; doi:10.1038/s43018-022-00356-3)
Supplement: Supplementary file 1 — Supplementary Notes 1–11 and figures. [file 43018_2022_356_MOESM1_ESM.pdf]

nature portfolio

https://doi.org/10.1038/s43018-022-00356-3

Supplementary information

Cell type and gene expression deconvolution with BayesPrism enables Bayesian integrative analysis across bulk and single-cell RNA sequencing in oncology

In the format provided by the authors and unedited

Supplementary Note 1

Overview: BayesPrism (Bayesian) vs. frequentist regression-based approaches

Frequentist approaches, including most regressions and their regularized versions, assume the accuracy of reference expression matrix (under some error distribution) to derive a point estimate of cell type compositions. This assumption is often violated in the deconvolution of transcription profiles due to technical or biological variation between reference and bulk data of interest. This problem is particularly pronounced in cancer, where the transcription profile of malignant cells is highly heterogeneous across patients, a phenomenon largely attributed to the unique landscape of somatic mutations in individuals. Therefore, a reference expression matrix does not generalize to malignant cells in unobserved patients. In addition, batch effects between single cell reference and bulk tumor samples makes the reference matrix inaccurate.

To overcome these difficulties, BayesPrism uses φ, the reference expression matrix observed from scRNA-seq, as prior information which together with the prior on μ induces a prior distribution on the cell state-specific expression matrix U. The joint posterior distribution on μ and U is updated when X, the bulk RNA-seq information is observed. Note that the inference on U is not possible in frequentist approaches, where U either equals φ or not. By explicitly modeling the expression profile U in the bulk sample, BayesPrism accounts for the inaccuracy of the reference matrix φ.

1. Gibbs sampling on cell state composition and gene expression

The inference of $P(\mu, U \mid \varphi, X; \alpha)$ in the first sampling of BayesPrism resembles sub-problems in the inference of latent Dirichlet allocation[1]. For readers who are familiar with work of topic models such latent Dirichlet allocation Blei et. al., each read in bulk RNA-seq is equivalent to a word; each bulk RNA-seq sample is equivalent to a document; each cell state is equivalent to a topic; each gene is equivalent to a vocabulary. The difference between BayesPrism and LDA is that in LDA the topic-word distribution, φ, is unknown and to be inferred from the posterior, while BayesPrism assumes it is known from the expression profile of scRNA-seq. The traditional sampling-based approach for LDA inference relies on the use of Gibbs sampling to approximate the posterior distribution of the topic distribution of each word[2], from which the topic-word distribution and document-topic distribution can be derived. This approach is computationally costly for inferring RNA-seq, where the word (read) number is typically at the scale of 10^8 for one document (bulk RNA-seq sample). Besides, biological applications need not to know the posterior for each word, but are rather interested the expression level of each gene. Zhu and Lei et al. [3] recently improved the computational efficiency by sampling at the gene level through the introduction of augmented latent variables, such that the time complexity is only a function of number of genes, which are usually between 10K-50K, and is independent of sequencing depth. We follow their derivation to get the formula for the first Gibbs sampling of BayesPrism's, and provide a more detailed step-by-step derivation, and adding back missing terms in their likelihood function.

The full model specification is as follows. Each bulk sample $n \in \{1, \ldots, N\}$ is measured across G

annotated genes, and the bulk RNA-seq data is represented by a matrix $X \in \mathbb{R}^{N \times G}$. As the genes that each read aligned to is also observed, each row of X, denoted by X_n, can be expanded to $\tilde{X}_{n,r} \in \mathbb{R}^G$ to denote the gene that the rth read in the nth sample aligned to. We assume that the cell states of each cell of the scRNA-seq dataset are known. The reference expression profiles of a total S cell states estimated from the scRNA-seq data denoted by $\varphi \in \mathbb{R}^{S \times G}$. We model the distribution of read counts of scRNA-seq in each cell state using multinomial distribution, and hence each row of $\varphi_s \in \mathbb{R}^G$ is an maximum likelihood estimator of the multinomial distribution event probability parameters, such that $\sum_{g=1}^{G} \varphi_{s,g} = 1$, for $\forall s \in \{1, \ldots, S\}$. To avoid having zeros in φ, for each φ_s we compute the pseudo count such that the $\min(\varphi_{s,g}) = 10^{-8}$ after renormalizing to one. $\mu \in \mathbb{R}^{N \times S}$ denotes the fraction of reads assigned to the sth cell state in the nth bulk RNA-seq sample. $Y_{n,r}$ denotes the latent variable describing the cell state that the rth read of the nth sample belongs to. α is the hyper-parameter of the Dirichlet prior on μ. We set $\alpha = 10^{-8}$ to represent a weak and non-informative prior, such that the posterior is mainly driven by the likelihood. The augmented cell state expression tensor $U \in \mathbb{R}^{N \times S \times G}$ is defined as $U_{n,s,g} = \sum_{\{r: X_{r,n} = g\}} I_{\{Y_{n,r}=s\}}$, which denotes the number of reads assigned to the gth gene in s^{th} cell state of the nth bulk sample.

The generative process is described as follows.

1. Generate fractions for tumor and environmental cells:

   $\mu_n \sim$ Dirichlet (α), i.i.d. for $n \in \{1, \ldots, N\}$, and $\alpha > 0$.

2. Generate the reads for bulk RNA-seq

   $Y_{n,r} \mid \mu_n \sim$ Categorical (μ_n), independently for $n \in \{1, \ldots, N\}$, and $r \in \{1, \ldots, R_n\}$

   $\tilde{X}_{n,r} \mid Y_{n,r}, \varphi \sim$ Categorical ($\varphi_{Y_{n,r}}$), i.i.d. for $r \in \{1, \ldots, R_n\}$

   $X_{n,g} = \sum_{r=1}^{R_n} I_{\{\tilde{X}_{n,r}=g\}}$

   $U_{n,s,g} = \sum_{\{r: X_{r,n} = g\}} I_{\{Y_{n,r}=s\}}$

   *Note that the distribution is written as multinomial distribution in Methods, which is an equivalent but more compact representation of the categorical distribution. We expand it here using the categorical distribution to facilitate understanding and downstream derivation.

To derive the posterior of P(μ, U | φ, X; α), we first write down the complete likelihood function:

$$p(X, Y, U, \mu \mid \varphi; \alpha) = p(\mu \mid \alpha)\, p(Y \mid \mu)\, p\,(\tilde{X} \mid Y)\, p(X \mid \tilde{X})$$

$$\propto \prod_{n=1}^{N} \left\{ \begin{array}{c} \Gamma(S \cdot \alpha) \prod_{s=1}^{S} \frac{\mu_{n,s}{}^{(\alpha-1)}}{\Gamma(\alpha)} \cdot \prod_{r=1}^{R_n} \prod_{s=1}^{S} \mu_{n,s}{}^{I_{\{Y_{n,r}=s\}}} \cdot \\ \prod_{s=1}^{S} \prod_{g=1}^{G} \varphi_{s,g}{}^{\sum_{r:\tilde{X}_{n,r}=g} I_{\{Y_{n,r}=s\}}} \cdot \prod_{g=1}^{G} I_{\{X_{n,g}=\sum_{r=1}^{R_n} I_{\{\tilde{X}_{n,r}=g\}}\}} \end{array} \right\} \tag{1}$$

Further observe that

$$\prod_{r=1}^{R_n}\prod_{s=1}^{S}\mu_{n,s}{}^{I_{\{Y_{n,r}=s\}}}$$
$$=\prod_{s=1}^{S}\mu_{n,s}{}^{\sum_{r=1}^{R_n} I_{\{Y_{n,r}=s\}}}$$
$$=\prod_{s=1}^{S}\mu_{n,s}{}^{\sum_{g=1}^{G}\sum_{r=1}^{R_n} I_{\{Y_{n,r}=s\ \&\ \tilde{X}_{n,r}=g\}}}$$
$$=\prod_{s=1}^{S}\mu_{n,s}{}^{\sum_{g=1}^{G} U_{n,s,g}}$$
$$=\prod_{g=1}^{G}\prod_{s=1}^{S}\mu_{n,s}{}^{U_{n,s,g}} \tag{2}$$

$$\prod_{s=1}^{S}\prod_{g=1}^{G}\varphi_{s,g}{}^{\sum_{r:\tilde{X}_{n,r}=g} I_{\{Y_{n,r}=s\}}}$$

$$=\prod_{g=1}^{G}\prod_{s=1}^{S}\varphi_{s,g}{}^{U_{n,s,g}} \tag{3}$$

and,

$$I_{\{X_{n,g}=\sum_{r=1}^{R_n} I_{\{\tilde{X}_{n,r}=g\}}\}}$$
$$=I_{\{X_{n,g}=\sum_{r=1}^{R_n}\sum_{s=1}^{S} I_{\{\tilde{X}_{n,r}=g\ \&\ Y_{n,r}=s\}}\}}$$
$$=I_{\{X_{n,g}=\sum_{s=1}^{S} U_{n,s,g}\}} \tag{4}$$

Combining 1-4, get

$$(1)=\prod_{n=1}^{N}\left\{\begin{matrix}\Gamma(S\cdot\alpha)\ \prod_{s=1}^{S}\frac{\mu_{n,s}{}^{(\alpha-1)}}{\Gamma(\alpha)}\cdot\prod_{g=1}^{G}\prod_{s=1}^{S}(\mu_{n,s}\varphi_{s,g})^{U_{n,s,g}}\cdot \\ \prod_{g=1}^{G} I_{\{X_{n,g}=\sum_{s=1}^{S} U_{n,s,g}\}}\end{matrix}\right\}$$

Therefore,

$$p(\mu_{n,\cdot}\mid X,U,\varphi;\alpha)\ \propto\ \prod_{s=1}^{S}\mu_{n,s}{}^{(\sum_{g=1}^{G} U_{n,s,g}+\alpha-1)},$$
$$p(U_{n,\cdot,g}\mid \mu,X,\varphi;\alpha)\ \propto\ \prod_{s=1}^{S}(\mu_{n,s}\varphi_{s,g})^{U_{n,s,g}}\ I_{\{X_{n,g}=\sum_{s=1}^{S} U_{n,s,g}\}} \tag{5}$$

Their corresponding distributions can then be read off from (5):

$$\mu_{n,\cdot}\mid X,U,\varphi;\alpha\sim Dirichlet\ (\alpha+\ \textstyle\sum_{g=1}^{G} U_{n,\cdot,g}\ ),$$
$$U_{n,\cdot,g}\mid \mu,X,\varphi;\alpha\sim Multinomial\ (\frac{\mu_{n,\cdot}\odot\varphi_{\cdot,g}}{\sum_{s=1}^{S}\theta_{n,s}\varphi_{s,g}},X_{n,g}),$$

where $\odot$ is element-wise multiplication. (6)

We initiate μ to a value of $1/S$. As each sample is conditionally independent, they can be sampled in parallel. Empirically, the Gibbs chain converges fairly fast. This is mainly due to the high read depth of bulk RNA-seq samples, which gives a fairly concentrated posterior distribution on μ. The default setting for Gibbs sampling is as follows: length of chain = 1000; burn in = first 500; thinning =2. BayesPrism reports the mean of posterior, E[U] and E[μ]. In practice, the Gibbs chain converges very fast due to the large number of reads in the bulk (Fig. SN1):

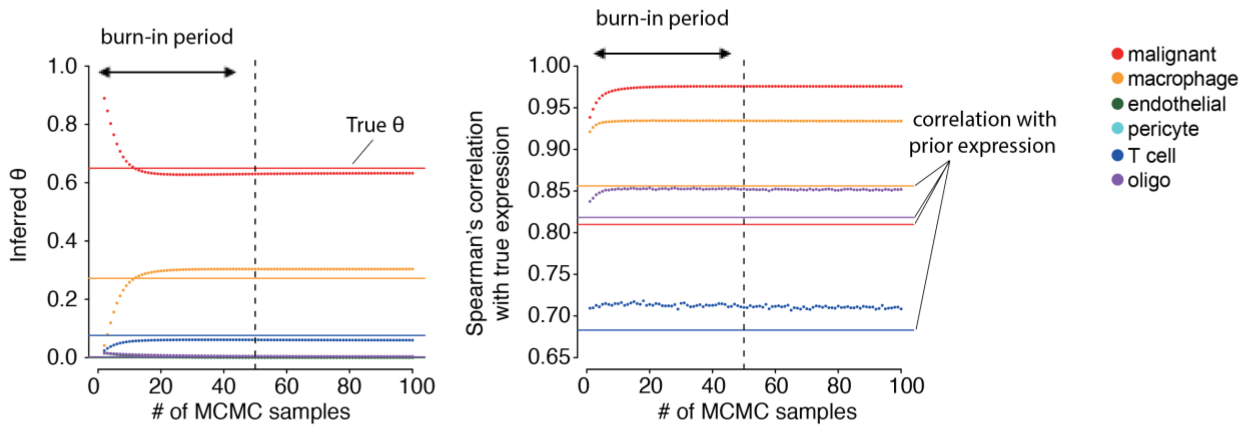

Fig. SN1. MCMC diagnostic plots for cell type fractions (left) and Spearman's correlations between the inferred gene expression and true gene expression. X axis marks the number of MCMC samples, i.e. chain length. The horizontal lines in the **left** panel mark the true cell type fractions, while those on the **right** panel mark the Spearman's correlations between the gene expression of the scRNA-seq prior and the true gene expression. The posterior quickly reaches a concentrated and unimodal stationary distribution after around 20 MCMC cycles.

2. update the reference matrix ψ for malignant and non-malignant cells

To estimate the malignant expression reference in each patient, we model the likelihood using multinomial distribution with the parameter $\psi_{mal_n} \in \mathbb{R}^G$, and get the maximum likelihood estimator:

$$\underset{\psi_{mal_n}}{\operatorname{argmax}}\ p\left(Z_{n,t,\cdot}\ \middle|\ \psi_{mal_n}\right) = \frac{Z_{n,t,g}}{\sum_{g=1}^{G} Z_{n,t,g}}\ , where\ t = malignant \tag{7}$$

We also use the multinomial distribution to model the likelihood function for the expression profiles of non-malignant cells shared across all patients. In this step we pooled the observations of Z across all bulk RNA-seq samples for each non-malignant cell to borrow the statistical strength across samples. To prevent pathological cases where subsets of non-malignant cells have a close to zero fraction in all bulk RNA-seq, in which case the Z will be close to zero and hence dominated by the sampling noise, we put a zero-mean log normal-distributed prior on the fold change with respect to their corresponding reference φ' (the scRNA-seq reference matrix defined over cell types, similar to φ), and use the maximum a posteriori (MAP) estimator for ψ_{env}

$$D_t = log(\,p(\,\psi_{env_t}|\,Z_{\cdot,t,\cdot}\,, \varphi'_t; \sigma)) \tag{8}$$

$$= \sum_{n=1}^{N} log(p(\,Z_{n,t,\cdot}|\,\psi_{env_t})) + log(\,p(\psi_{env_t}\,|\,\varphi'_t; \sigma\,)) + C$$

$$= \sum_{n=1}^{N}\sum_{g=1}^{G} Z_{n,t,g} \log\left(\psi_{env_{t,g}}\right) - \frac{1}{2\sigma^2}\sum_{g=1}^{G} \log\left(\frac{\psi_{env_{t,g}}}{\varphi'_{t,g}}\right)^2 + C', where\ t \in \{malignant\}^c$$

The MAP estimator of D_t has no closed form solution and needs to be optimized numerically. Directly optimizing over ψ is more difficult, due to the constraint that $\sum_{g=1}^{G} \psi_{env_{t,g}} = 1$. We therefore used the change of variables method, by letting $\gamma_{t,g} = \log(\frac{\psi_{str_{t,g}}}{\varphi'_{t,g}})$, and $\psi_{env_{t,g}} = \frac{\varphi'_{t,g} \cdot \gamma_{t,g}}{\sum_{g=1}^{G} \varphi'_{t,g} \cdot \gamma_{t,g}}$, to make the optimization unconstrained and numerically more stable (see below).

$$D_t = \sum_{g=1}^{G} (\sum_{n=1}^{N} Z_{n,t,g}) \log (\frac{\varphi_{env_{m,g}} \cdot \gamma_{m,g}}{\sum_{g=1}^{G} \varphi_{env_{m,g}} \cdot \gamma_{m,g}}) - \frac{1}{2\sigma^2} \sum_{g=1}^{G} {\gamma_{t,g}}^2 + C' \quad (9)$$

$\sum_{n=1}^{N} Z_{n,t,g}$ can be computed before optimization, and let $Z_{t,g} = \sum_{n=1}^{N} Z_{n,t,g}$. With some algebra, the partial derivative of the posterior can be derived as

$$\frac{\partial D_t}{\partial \gamma_{t,g}} = Z_{t,g} - \psi_{env_{t,g}} (\sum_{g=1}^{G} Z_{t,g}) - \frac{1}{\sigma^2} \gamma_{t,g} \quad (10)$$

As $\frac{\partial D_t}{\partial \gamma_{t,g}}$ is a function of only γ_t, they can be optimized in parallel. We use the conjugate gradient method written by the Rcgmin package[4] with minor modifications by adding a stopping criterion to prevent it making more than 500 consecutive non-productive iterations. All γ are initiated at 0. By default, we set $\sigma = 2$, for all genes, which is around the typical range of log fold change between two batches of RNA-seq dataset and is a very weak prior compared to the likelihood. Users may also supply their own σ based on their prior knowledge, for example the standard deviation of log fold change from differential expression analysis between a pair of matched samples, or the closest scRNA-seq reference and bulk RNA-seq samples.

3. Learning latent embeddings for malignant gene programs.

3.1 Model specification

The goal of learning the latent embeddings is to approximate the expression of malignant cells across a cohort of N bulk RNA-seq samples as a linear combination of a small number of K bases, with K<<N. Complete factorization approaches, such as NMF and LDA, aim at factorizing the bulk RNA-seq down to a linear combination of K bases (gene programs), with K<<N. However, gene programs inferred by these approaches will be confounded by the expression of non-malignant cells. Additionally, expression in malignant cells is highly heterogenous (i.e., in practice, K=N for this problem), and hence reducing K to a number significantly less than N is a lossy compression. To overcome these limitations, the strategy used by BayesPrism differs in that it conditions on fraction and expression profile of non-malignant cells, i.e. θ_{env} and ψ_{env} , inferred by the deconvolution module, and learns the embeddings of malignant cells.

We denote the initial K tumor basis as $\eta_0 \in \mathbb{R}^{K\times G}$, and a set of perturbations from η_0 to be inferred as $\lambda \in \mathbb{R}^{K\times G}$, their associated weights as $\omega \in \mathbb{R}^{N\times K}$. The total number of components in the embedding learning is then $M = K + T - 1$.

The probabilistic graphical model is shown by **Fig. SN2** below:

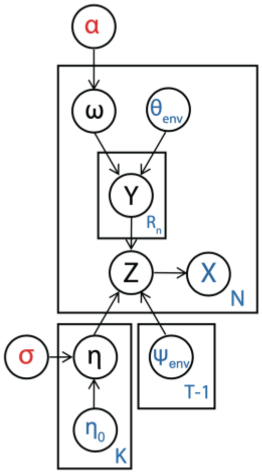

Fig. SN2. Graphical model illustrates the statistical dependencies and the generative process for the observed bulk RNA-seq data, X. Red text marks hyper-parameters; blue marks observed variables; black marks latent variables.

The generative process is as follows.

1. Generate weights for tumor basis:

$\kappa_n \sim$ Dirichlet (α), i.i.d. for $n \in \{1, \ldots, N\}$, where $\alpha \in \mathbb{R}^K$ and $\alpha > 0$.

$\omega_n = \tau_n \kappa_n$, where $\tau_n = 1 - \sum_{t \in \{malignant\}^c} \theta_{n,t}$

Concatenating the columns of ω and θ_{env} , we get $\nu \in \mathbb{R}^{N \times M}$, where $\sum_{m=1}^{M} \nu_{n,m} = 1$.

*Note that α need not be the same as the one in equation (1). By default, however, BayesPrism uses the same α for a non-informative sparse prior.

2. Generate the full expression profiles:

$\log(\lambda_{k,g}) \sim$ Normal $(0, \sigma)$, for $g \in \{1, \ldots, G\}$ and $k \in \{1, \ldots, K\}$

*Note that σ need not be the same as the one in equation (8). By default, however, BayesPrism uses the same σ.

$$\eta_{k,g} = \frac{\eta_{0_{k,g}} \cdot \lambda_{k,g}}{\sum_{g=1}^{G} \eta_{0_{k,g}} \cdot \lambda_{k,g}} \text{ for } k \in \{1, \ldots, K\}$$

Concatenating the rows of η and ψ_{env}, we get the total expression profile $\zeta \in \mathbb{R}^{M \times G}$

3. Generate the reads for bulk RNA-seq:

$Y_{n,r} \mid \nu_n \sim$ Categorical (ν_n), independently for $n \in \{1, \ldots, N\}$, and $r \in \{1, \ldots, R_n\}$

$\tilde{X}_{n,r} \mid Y_{n,r}, \zeta \sim$ Categorical $(\zeta_{Y_{n,r}})$, i.i.d. for $r \in \{1, \ldots, R_n\}$

$X_{n,g} = \sum_{r=1}^{R_n} I_{\{\tilde{X}_{n,r}=g\}}$

$V_{n,m,g} = \sum_{\{r: X_{r,n}= g\}} I_{\{Y_{n,r}=m\}}$

2.2 Model inference using Expectation-maximization (EM)

As ω, η, and Z are all latent variables and are marginally dependent on each other, direct inference can be difficult. Considering the advantage of marginalizing nuance variables[5], we use the Expectation-maximization (EM) algorithm to optimize η while marginalizing ω and V.

The EM algorithm is formulated as follows. In the M step the posterior we would like to maximize $E_Q[\log(p(\lambda,\ V \mid X, \eta_0,\ \psi_{env}, \theta_{env}\ ;\ \sigma))]$, with the expectation taken over $Q = p(\ V \mid \psi_{env}, \theta_{env}, \eta^{old};\ \sigma, \alpha)$, i.e. the posterior sampled by Gibbs sampling in the E step, which constitutes the Gibbs-EM algorithm[6].

2.2.1 The E step

The complete likelihood function for the E step is

$$p(\omega, Y, \tilde{X}, X \mid \eta^{old}, \psi_{env}, \theta_{env}\ ;\ \sigma, \alpha) = p(\omega \mid \tau\ ; \alpha)\ p(Y \mid \nu, \zeta)\ p\ (\tilde{X} \mid Y)\ p(X \mid \tilde{X})$$
$$= \prod_{n=1}^{N}\{p(\omega_n \mid \tau_n\ ; \alpha)\ \prod_{r=1}^{R_n} p(Y_r \mid \nu,\ \zeta)\ p\ (\tilde{X}_r \mid Y_r)\ p(X_r \mid \tilde{X}_r\ )\}$$

Observe that the posterior of $p\big(V_{n,\cdot,g} \mid \nu, X, \zeta; \alpha\big)$ follows the same form as the $p\big(U_{n,\cdot,g} \mid \mu, X, \varphi; \alpha\big)$ in (5). Hence we have:

$$p\big(V_{n,\cdot,g} \mid \nu, X, \zeta; \alpha\big) \sim Multinomial\ \Big(\frac{\nu_{n,\cdot} \odot \zeta_{\cdot,g}}{\sum_{m=1}^{M} \nu_{n,m}\zeta_{m,g}}, X_{n,g}\Big),$$

where ⊙ is element-wise multiplication.

Sampling from $p\big(\omega_{n,\cdot} \mid X, V, \zeta; \alpha\big)$ is slightly different from (5), due to the scaling factor τ_n. Since ω is deterministic of κ, sampling ω is trivial when one can sample from the posterior of κ, i.e. $p\big(\kappa_{n,\cdot} \mid X, V, \zeta; \alpha\big)$, which we derive as follows:

$$p\big(\kappa_{n,\cdot};\ \alpha\big) \propto \prod_{k=1}^{K} {\kappa_{n,k}}^{\alpha-1}$$

$$p\big(L_{n,\cdot} \mid \kappa_{n,\cdot}, \theta_{n,\cdot}\big) \propto \prod_{k=1}^{K} (\tau_n \kappa_{n,k})^{L_{n,k}} \prod_{t\in\{malignant\}^c} {\theta_{n,t}}^{O_{n,t}} = \prod_{k=1}^{K} {\kappa_{n,k}}^{L_{n,k}} \cdot C$$

, where $L_{n,k}=\sum_{g=1}^{G} V_{n,k,g}$, for k∈{1,…,K}, and $O_{n,t}=\sum_{g=1}^{G} V_{n,t,g}$, for t∈{K+1,…,T-1}

Therefore, $p(\kappa_{n,\cdot} \mid X, V, \zeta; \alpha) \propto p\big(\kappa_{n,\cdot};\ \alpha\big) \cdot p\big(L_{n,\cdot} \mid \kappa_{n,\cdot}, \theta_{n,\cdot}\big) \propto \prod_{k=1}^{K} {\kappa_{n,k}}^{L_{n,k}+\alpha-1}$.

The conditional distribution follows $\kappa_{n,\cdot} \mid X, V, \zeta; \alpha \sim Dirichlet\ (\alpha + L_{n,\cdot})$.

2.2.1 The M step

In the M step, we aim to find $\underset{\eta}{\operatorname{argmax}}\ E_Q[\log(\ p(\eta, V\ |X, \eta_0, \psi_{env}\ , \theta_{env};\ \sigma))]$, with Q being the Gibbs samples describing the posterior of V sampled at the E step. $\underset{\eta}{\operatorname{argmax}}\ E_Q[\log(\ p(\eta, V, |X, \eta_0,$ $\psi_{env}\ , \theta_{env};\ \sigma, \alpha))]$ is a constrained optimization, with $\sum_{g=1}^{G} \eta_{k,g} = 1$ for all ∀ k∈ {1, … , K }. We again turn this into an un-constrained optimization by optimizing its equivalent form $\underset{\lambda}{\operatorname{argmax}}\ E_Q[\log(\ p(\lambda, V, |X, \eta_0,\ \psi_{env}\ , \theta_{env};\ \sigma))]$.

The complete log posterior written in the augmented form is

$$
\begin{aligned}
&E_Q[log(p(\ \lambda, V\ |X, \eta_0, \psi_{env}, \theta_{env};\ \sigma))] \\
&= E_Q[log(p(\ V, X \mid \lambda, \eta_0, \psi_{env}, \theta_{env}))]\ + log(p(\lambda;\ \sigma))\ + C \\
&= E_Q[log(p(\ V \mid \lambda, \eta_0, \psi_{env}, \theta_{env}))] + log(p(\lambda;\ \sigma))\ + C \\
&= \ \sum_{g=1}^{G} \sum_{k=1}^{K} E_Q[log(p(\ V_{n,k,g} | \lambda_{m,g}, \eta_{0_{m,g}}))]\ \} + \ \sum_{g=1}^{G} \sum_{k=1}^{K} log(p(\lambda_{k,g};\ \sigma))\ + C' \\
&= \sum_{n=1}^{N} \sum_{g=1}^{G} \sum_{k=1}^{K} E_Q\left[V_{n,k,g} \log\left(\zeta_{k,g}\right)\right] + \ \sum_{g=1}^{G} \sum_{k=1}^{K} \log\left(p\left(\lambda_{k,g};\ \sigma\right)\right)\ + C''
\end{aligned}
$$

The constants C, C' and C" are with respect to the parameter λ.

Similar to the derivation of (9) and (10), the posterior and its derivative are

$$D_k = \sum_{g=1}^{G} \left(\sum_{n=1}^{N} E_Q[V_{n,k,g}]\right) \log\left(\frac{\eta_{0_{k,g}} \cdot \lambda_{k,g}}{\sum_{g=1}^{G} \eta_{0_{k,g}} \cdot \lambda_{k,g}}\right) - \frac{1}{2\sigma^2} \sum_{g=1}^{G} {\gamma_{k,g}}^2 \qquad (12)$$

Since $\sum_{n=1}^{N} E_Q[V_{n,k,g}]$ can be computed before optimization, and we can let $V_{k,g} = \sum_{n=1}^{N} E_Q[V_{n,k,g}]$, with some algebra, the partial derivative of the posterior can be derived as

$$\frac{\partial D_k}{\partial \gamma_{k,g}} = V_{k,g} - \eta_{k,g}\left(\sum_{g=1}^{G} V_{k,g}\right) - \frac{1}{\sigma^2} \gamma_{k,g} \qquad (13)$$

As $\frac{\partial D_k}{\partial \gamma_{k,g}}$ is a function of only γ_k, they can be optimized in parallel for each k. The optimization was implemented by the conjugate gradient method in the same way as for ψ_{env}. σ=2 is a very weak prior compared to the likelihood, and mainly serves as a way to prevent zero probability in η during EM. There are several ways to set η_0 and K. η_0 can be set identically for all K by taking an average of the tumor expression profiles, ψ_{mal}, estimated in equation (7). Despite being initialized equally, γ_k will eventually diverge and pick up a gradient after 10-20 EM cycles, due to the randomness of Gibbs-sampling. In practice this approach takes more EM cycles to converge. Alternatively, user may use the NMF to factorize ψ_{mal} , and pick the optimum K based on criteria used for consensus clustering, e.g. cophenetic correlation, dispersion, silhouette score, etc [7]. This approach converges much faster than a uniform initialization, and typically converges within 50 cycles (**Fig. SN3**). Biological prior knowledge can also be used to initiate η_0 using the expression of known subtypes or origin cells of malignant cells.

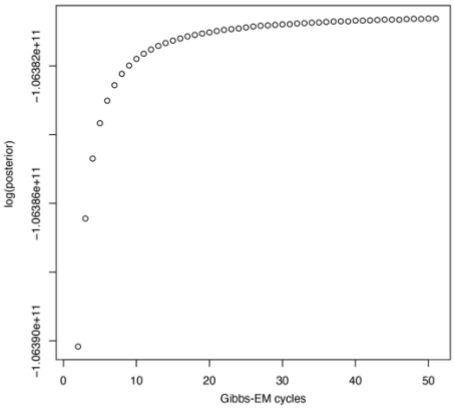

Fig.SN3. log(posterior) of the model as a function of EM cycles.

Reference

1. Blei, D. M., Ng, A. Y. & Jordan, M. I. Latent Dirichlet allocation. *J. Mach. Learn. Res.* (2003). doi:10.1016/b978-0-12-411519-4.00006-9
2. Griffiths, T. L. & Steyvers, M. Finding scientific topics. *Proc. Natl. Acad. Sci. U. S. A.* (2004). doi:10.1073/pnas.0307752101
3. Zhu, L., Lei, J., Devlin, B. & Roeder, K. A unified statistical framework for single cell and bulk rna sequencing data. *Ann. Appl. Stat.* (2018). doi:10.1214/17-AOAS1110
4. Nash, J. C. Conjugate Gradient Minimization of Nonlinear Functions with Box Constraints. R package version 2013-02.20, URL http://CRAN.R-project.org/ package=Rcgmin. (2013).
5. Berger, J. O., Liseo, B. & Wolpert, R. L. Integrated likelihood methods for eliminating nuisance parameters. *Stat. Sci.* (1999). doi:10.1214/ss/1009211804
6. Dupuy, C. & Bach, F. Online but accurate inference for latent variable models with local Gibbs sampling. *J. Mach. Learn. Res.* (2017).
7. Gaujoux, R. & Seoighe, C. A flexible R package for nonnegative matrix factorization. *BMC Bioinformatics* (2010). doi:10.1186/1471-2105-11-367

Supplementary Note 2: Benchmarking computational efficiency

As CIBERSORTx is based on SVR which scales cubically ($O(N^3)$) as the number of genes, and does not parallelize for each bulk sample, it does not scale to datasets with large numbers of marker genes. In contrast, BayesPrism scales linearly ($O(N)$), and takes advantage of parallelization (see **Fig. SN4**). To make the benchmark reasonably computable, we downsampled the ⅕ of the 1,350 pseudo-bulks for comparison in **Fig.1c-d**.

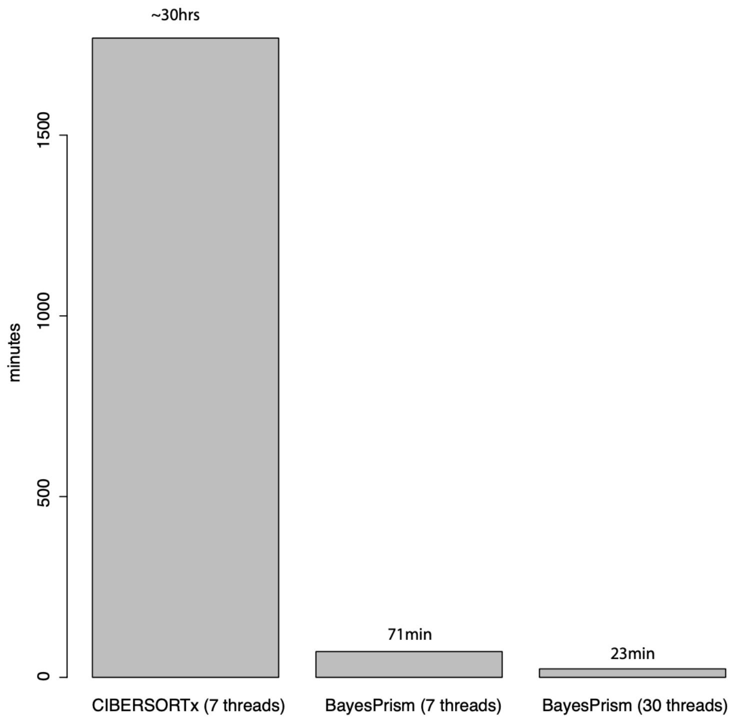

Fig. SN4. | The estimated computing time of the online version of CIBERSORTx and BayesPrism. The computing time of BayesPrism was tested on Intel(R) Xeon(R) CPU E5-4620 v2 @ 2.60GHz.

Supplementary Note 3: Mathematical intuition of the robustness of BayesPrism

In this supplementary note, we discuss the intuition of the robustness of BayesPrism by demonstrating that BayesPrism is mathematically invariant to two types of noise that represent idealized cases of technical batch effects and biological variation. The proof of invariance follows from the formula used to perform Gibbs sampling, as derived in the equation (6) in the **Supplementary Note 1**, and copied below.

$$\mu_{n,\cdot} \mid X, U, \varphi; \alpha \sim Dirichlet\,(\alpha + \textstyle\sum_{g=1}^{G} U_{n,\cdot,g}\,),$$
$$U_{n,\cdot,g} \mid \mu, X, \varphi; \alpha \sim Multinomial\,\left(\frac{\mu_{n,\cdot} \odot \varphi_{\cdot,g}}{\sum_{s=1}^{S} \theta_{n,s}\varphi_{s,g}}, X_{n,g}\right),$$
$$\text{where } \odot \text{ is element-wise multiplication.} \qquad (6)$$

The first type of noise is linear multiplicative noise, where the true reference in the bulk RNA-seq differs from the observed reference in scRNA-seq by a multiplicative constant:

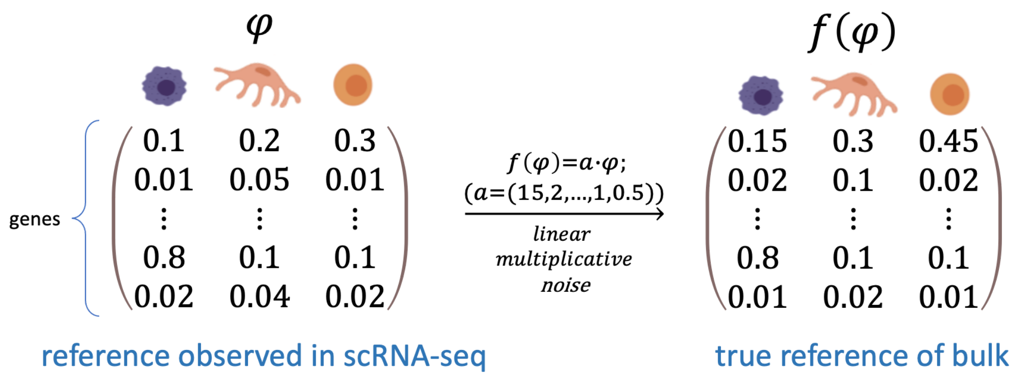

Notice that under such noise, the relative ratios of the expression of each gene between cell types from the observed reference are the same as the true reference, and hence this noise mimics the technical batch effects where genes are captured at different efficiencies by two experimental / sequencing methods. By replacing φ with $a \cdot \varphi$ in (6), it is obvious that the posterior of U remains the same, as the constant cancels out. Therefore, BayesPrism is invariant to such noise. **Extended Data Fig. 2** simulates the performance of BayesPrism and other deconvolution tools under such a noise model.

The second type of noise is any arbitrary transformation on marker genes expressed exclusively in one cell type, provided that the expression in other cells remains zero after the transformation:

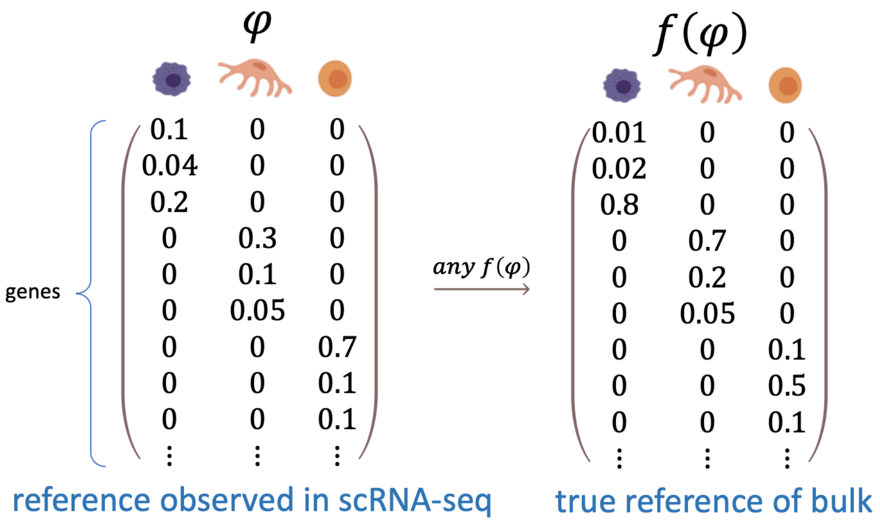

Under such noise, it is also obvious that the posterior of U remains the same, and reads from the marker genes of a particular cell type will be exclusively assigned to that cell type, resulting in a Dirac posterior, and hence a rapid convergence of the Gibbs chain in one sampling step. This noise model mimics highly cell type specific genes that retain their cell type specificity under both technical batch effects and biological variation. It is likely that the combination of the invariance to the two types of noise contributes to the robustness of BayesPrism.

Supplementary Note 4: Performance Benchmarks

Benchmarks against other deconvolution tools. We benchmarked BayesPrism against CIBEROSRTx, Bseq-SC, Bisque, SCDC, and MuSiC. Details of the datasets used for the benchmark were listed in **Supplementary Table 1**. Marker genes are required for CIBERSORT-based methods including Bseq−SC and CIBERSORTx (all modes), while they are optional for all other methods including BayesPrism. For CIBERSORTx we used the online portal of CIBERSORTx (https://cibersortx.stanford.edu) to perform all the benchmarking. All parameters were used at default values, except for the "Min. Expression", which was set to 0 for single cell references to generate a signature matrix, following the author's recommendations for droplet-based platforms. Quantile normalization is disabled by default following the author's recommendations for RNA-seq. For all other methods, we used the corresponding R packages. In all non-tumor sample deconvolutions, we used a single batch of scRNA-seq dataset as the reference for Bseq-SC, SCDC, and split the scRNA-seq dataset equally into two batches for each cell type for Bisque and MuSiC, as multiple batches of single cell references are required by these methods. When benchmarking Bisque we used the "ReferenceBasedDecomposition" and disabled "use.overlap", as we did not have samples with matched scRNA-seq and bulk RNA-seq. For benchmarking deconvolution of tumor samples (GBM28 pseudo-bulk), we leveraged the information of individual patients from scRNA-seq reference (GBM8) to label biological replicates whenever possible. This includes Bseq-SC, Bisque and MuSiC. Malignant cells in each patient were used as an individual cell type. As only one patient in GBM8 contains T cells, MuSic was unable to model its variance, resulting in the missing T cell from its scRNA-seq reference. To circumvent this, we split the T cells equally into two batches.

Gene filtering and feature selection. When deconvolving genes without markers, ribosomal and mitochondrial genes, were removed in all benchmarks (see **Supplementary Note 5** for details). Genes on chromosome X and Y were also removed to prevent sex-specific differences between the scRNA-seq reference and the pseudo-bulk sample. To speed up computation, we removed lowly expressed genes, by selecting genes expressed in at least 5 cells. In addition, outlier genes, defined as genes that show >1% of total reads (or normalized reads if only TPM data is available) in at least one bulk sample were removed, unless otherwise specified below. In all pseudo-bulk analysis, we defined the ground truth as fractions of total reads over all annotated genes in each cell type. We benchmarked using this metric against other deconvolution tools, as the fraction of reads is proportional to the total fraction of cell counts, and hence the cell type level correlation will not be affected.

When benchmarking deconvolution using markers, we use the same set of markers generated by CIBERSORTx when applicable to ensure a fair comparison between all methods (**Fig. 1c-f; Extended Data Fig. 2**, labeled as "method name, w/ marker"). The BayesPrism package also provides the option for the use of marker genes to combat cases where significant batch effects exist, e.g. when using ribosomal-depleted RNA or statistical assumptions are possibly violated, e.g. when using references collected from unmatched samples. The implementation was based on the findMarker function from the scran package. Briefly, scRNA-seq reference was normalized by the median library size followed by $\log_2(X+0.1)$ transformation. There are two types of markers defined by the findMarker function, the "all marker" (genes that are significantly differentially transcribed between one cell type and all other cell types) and "any marker" (those that are significantly differentially transcribed between one cell type and any other cell type). Empirically, we found that all markers provided stronger

robustness to batch effects as they are closer to the exclusively expressed marker genes (the second type of noise in **Supplementary Note 3**). Statistical significance was calculated based on t-tests, and only genes upregulated in each cell type were used to define markers.

*Linear multiplicative noise model (***Extended Data Fig. 2***).* We used the scRNA-seq of PBMCs collected from the first donor using 10x Chromium (v2) A[1], as the reference, and we simulated 200 pseudo-bulk RNA-seq samples from the same dataset used to build the reference. The cell type fractions were drawn from a symmetric dirichlet distribution (α=1), and the cell count of each cell type was sampled from a multinomial distribution with the total cell number equal to the cell number in the original batch (N=3222). Cells were then sampled with replacement according to the simulated cell count of each cell type, and then aggregated by summing up the reads over the sampled single cells to make pseudo-bulks. In the simulation, no outliers from the bulk were removed.

To generate noise to the pseudo-bulks, we simulated a zero-centered log-normally distributed fold change at one particular noise level σ independently and identically distributed for each gene, which generated a vector of length equal to the number of genes. To mimic the real biological batch effects, we penalized extreme fold changes that resulted in unrealistic expression values, which was particularly frequent at high σ levels. This was done by sampling the fold change vector 10000 times and choosing the one that induced the minimal change to the total expression as measured by elemental-wise multiplying the reference expression with the fold change vector. The chosen fold change vector was then elemental-wise multiplied with the pseudo-bulks which were then rounded up to the nearest integers.

*Cross-platform deconvolution using pseudo-bulk RNA-seq from non-tumor samples (***Extended Data Fig. 3***).* For PBMC data, we used the 10x Chromium (v2) dataset collected from the second donor as the reference to deconvolve pseudo-bulks generated by the Smart-seq2 from the first donor in the original paper[1]. For mouse cortex data we used the sci-RNA-seq dataset collected from the second mouse as the reference to deconvolve pseudo-bulks generated by the Smart-seq2 from the first mouse in the original paper[1]. The choice of these datasets is to represent the strongest batch effect based on the correlation shown in the Supplementary Fig. 4 of the paper by Ding et al.[1].

*Single-platform leave-one-out deconvolution using pseudo-bulk RNA-seq from HNSCC, SKCM and OV (***Extended Data Fig. 4***).* We generated a "pseudo-bulk" RNA-seq dataset from one patient, and asked how accurately BayesPrism deconvolved expression using the reference constructed from the remaining patients. All parameters of BayesPrism and CIBERSORTx were at default. Batch effect correction was disabled for leave-one-out tests. Two cell types, "-Fibroblast" and "myocyte", in the HNSCC scRNA-seq dataset were of very low cell number <20 cells, and only showed up in a small subset of patients (N=4), which may lead to unreliable estimates of correlation coefficients. Therefore, we excluded them during the leave-one-out test. Benchmarking the expression inference could not be done, as CIBERSORTx requires the number of mixtures to be greater than the number of reference components.

As observed with GBM, BayesPrism consistently estimated cellular proportions that were more accurate to the true values than CIBERSORTx (**Extended Data Fig. 4a-d**). As the leave-one-out test data were generated from the same sequencing platforms and processed by a uniform pipeline, they represent minimal technical batch effects, and hence the superiority in performance of BayesPrism mainly reflects its ability to account for the deviation in the reference caused by inter-tumoral biological variation.

Cross-platform deconvolution using pseudo-bulk RNA-seq from GBM samples (***Fig. 1c-d; Extended Data Fig. 5-8, Supplementary Note 6****)*. We analyzed two glioblastoma multiforme (GBM) datasets collected from different patients using different scRNA-seq platforms to represent a mixture of technical and biological variation. One scRNA-seq reference analyzed 23,793 cells from 8 patients using a microwell-based platform[2] (GBM8), which sequenced tag clusters near the 3' end of polyadenylated genes, similar to other high-throughput scRNA-seq methods (e.g., Drop-seq, 10x genomics, etc). A second scRNA-seq dataset was available which sequenced 7,930 cells from 28 patients using the SMART-Seq2 platform[3] (GBM28), which sequenced full length mRNA transcripts to a high read depth in each cell, similar to most bulk RNA-seq datasets, and hence also mimicked differences between single cell and bulk.

We generated "pseudo-bulk" RNA-seq datasets from GBM28 by 1) aggreagating scRNA-seq counts for each patients (N=28), and 2) 1,350 pseudo-bulk RNA-seq samples by sampling random proportions of each cell type from a symmetric dirichlet distribution (α=1) and then aggregating reads from susampled cells using GBM28 with replacement. This dataset aims at testing BayesPrism across a wider range of different tumor compositions. The malignant cells in each simulated pseudo-bulk were restricted to an individual patient, while the non-malignant cells were drawn from all patients regardless of patient ID. 50 pseudo-bulk RNA-seq samples were simulated for each patient among 27 out of 28 GBM patients, with one sample BT1187 excluded due to only having 8 malignant cells. As raw data were TPM normalized, we rounded up the counts after summing them up across each cell. For comparison with CIBEROSRTx, we downsampled ⅕ of the 1,350 simulated samples, so that it could complete within a reasonable amount of wall time (~30hrs) (see **Supplementary Note 2** for details). We disabled batch correction when imputing gene expression for CIBERSORTx as both S mode and B mode batch correction resulted in worse performance than when no correction was used.

To construct the scRNA-seq reference, we treat each tumor subcluster as individual cell states (N=60), while summarizing each non-malignant cell type as a single reference (N=5). When benchmarking CBIERSORTx, we used the same cell state labeling by denoting each column of the single cell reference matrix as a cell phenotype, and then summing over weights across cell states within the malignant cells. For gene expression imputation in **Fig. 1h** and **Extended Data Fig. 6**, we summed up the imputed expression values across 60 malignant cell states to get the expression of malignant cells in each sample. Only 53 genes are imputable across all malignant states references by the high resolution mode (by excluding the "1" and "NA" values in the CIBERSORTxHiRes_job1_PJ0XX-tumor-X_Window140.txt). Correlation coefficients in **Extended Data Fig. 6** were computed on these 53 genes for four different approaches.

To show BayesPrism also infers the expression of non-malignant cells in addition to malignant cells, we generated an additional set of references and pseudo-bulks using GBM8 and GBM28, by incorporating heterogeneity in macrophages into our simulation (**Extended Data Fig. 8**). We first clustered macrophages found in GBM28 and GBM8. We processed the scRNA-seq of macrophages in each dataset as follows. As GBM28 data is TPM normalized, we skipped the normalization step, and $\log_2$ transformed the data followed by removal of ribosomal protein coding and mitochondrial genes, and genes on chromosome Y. We then filtered out genes expressed in less than 10 cells. We performed dimensionality reduction using the rsvd[4] package, using parameters k= 20, p= 15, q= 3. Phenograph was then used to cluster the macrophages over the 20 PCs imputed by SVD using the default parameter at K=30. Phenograph yielded 10 clusters for macrophages in GBM28. Similarly for macrophages in GBM8, we performed transformation, gene filtering, dimensionality reduction and clustering

using the same methods and parameters as described above. We used a medium library size normalization step followed by $\log_2$ transformation and clustering. Phenograph yielded 11 clusters for macrophages in GBM8. For each patient (N=27) and each macrophage cluster (N=10) in GBM28, we simulated 5 pseudo-bulk datasets, constituting 1,350 samples in total. The scheme of simulation was the same as for malignant cells, above. When deconvolving pseudo-bulks, we used 60 malignant states and 11 macrophage states. Vectors used to compute Pearson's correlation are of length equal to the total number of genes used to perform deconvolution, and represent zero centered variance-stabilizing transformed read counts for each gene. The cluster purity was calculated using the "purity" function from the NMF package.

*Real bulk RNA-seq of human whole blood with ground truth measured by flow-cytometry (**Fig.1e-f**).* To test the performance of BayesPrism on real bulk RNA-seq, we deconvolved 12 human whole blood samples for which the cell type composition was known using flow-cytometry. We used the same PBMC RNA-seq dataset from the CIBERSORTx paper as the reference, which was obtained from a patient with non-small cell lung cancer (NSCLC) using 10x Genomics Chromium v2 (3′ assay)[5]. The bulk PBMC dataset and scRNA-seq reference were mismatched, as the bulk RNA-seq reference was performed on whole blood and the scRNA-seq reference with PBMCs. Neutrophils present in high abundance in the whole blood sample were not represented in the reference because neutrophils are polynucleated and do not isolate with PBMCs. Missing neutrophils may inflate the fraction of other myeloid[6] cell types that have similar expression. Thus, we inferred the proportion over a total myeloid population and used the combined fraction of monocytes and neutrophils as the ground truth in all analyses. The bulk RNA-seq of human whole blood and the scRNA-seq reference of PBMCs from non-small cell lung cancer patients were downloaded from the CIBERSORTx website at: https://cibersortx.stanford.edu/download.php. As only the S mode of CIBERSORTx produced accurate results, as shown by the authors, we did not benchmark against the uncorrected and B mode.

Statistical test on cell type level Pearson correlation coefficients. To test the difference in the collection of cell type level Pearson correlation coefficients between BayesPrism and other methods, we first applied the Fisher's Z-transformation to the Pearson correlation coefficient *r* (sample correlation coefficient) of each cell type using the following formula:

$$z = \frac{1}{2} log(\frac{1+r}{1-r}) = artanh(r).$$

Fisher has shown that if the joint distribution of random variables *X* and *Y* is bivariate normal with correlation *ρ* (population correlation coefficient), then *z* is approximately normally distributed with mean $\frac{1}{2} log(\frac{1+\rho}{1-\rho})$ and standard error $\frac{1}{\sqrt{N-3}}$, where *N* is the sample size[7,8]. As a result, the difference in r of a particular cell type *c* between method i and j, is normally distributed with mean $\Delta z_{c(i,j)} = z_{c,i} - z_{c,j}$, and variance $2/(N - 3)$, and the mean of difference in *z* over *K* cell types between is normally distributed with mean $\overline{z_{i,j}} = \frac{1}{K}\sum_{c=1}^{K} \Delta z_{c(i,j)}$, and variance $2/(K \times (N - 3))$. We then performed one-sided z test with $H_0: \overline{z_{i,j}} < 0$, with i being BayesPrism, and j being other methods benchmarked.

*Comparing tumor purity with ABSOLUTE, ESTIMATE, IHC and CIBERSORTx (**Extended Data Fig. 9**).* As the tumor purity estimated by ABSOLUTE varies between multiple sources (Aran et. al. (2015) *Nature Communications*; Taylor et. al. (2018) *Cancer Cell*; TCGAN (2015) *Cell*) (leftmost columns in Source Data Extended Data Fig. 8), we took the mean of ABSOLUTE scores between two sources over a subset of samples whose difference between two sources is smaller than 0.1 (marked by the red dots), and used the averaged score for comparison with other methods. ESTIMATE and IHC scores were both obtained from Aran et al. CIERSORTx was run using the same input reference matrix as BayesPrism. One label was supplied for each cell type. We used the batch correction mode recommended by the author (S mode for GBM and OV; B mode for SKCM and HNSCC). In HNSCC we observed that ABSOLUTE score was lower than IHC, while tumor purity estimated using transcription signal (ESTIMATE, BayesPrism and CIBERSORTx) were higher than ABSOLUTE, suggesting an underestimation of tumor purity by the DNA copy-number based method, and/or the presence of non-immune non-stromal cells (such as normal epithelial cells, which was not captured by scRNA-seq reference) in the tumor samples.

Supplementary Note 5:
Effects of ribosomal protein coding genes and mitochondrial genes on deconvolution

When benchmarking deconvolution for several of the datasets under a marker-free mode, we found that the performance of all methods including BayesPrism improved when mitochondrial and ribosomal protein coding genes were excluded. These housekeeping genes were ubiquitously transcribed in all reference scRNA-seq datasets. The expression level of ribosomal protein coding genes ranges from 12% to 38% of the total mapped reads (human PBMC data[1]). They are often not informative in distinguishing cell types and can be a source of large spurious variance[9]. Cell types that were transcriptionally similar, such as CD4+ and CD8+ T cells, were more sensitive to these genes, as fewer genes were informative in distinguishing them. The accuracy of devonovolution is less sensitive to the expression level of mitochondrial genes, as their expression level is lower than that of ribosomal protein coding genes, ranging from 3% to 13% of total mapped reads (human PBMC data[1]). However, considering the level of mitochondrial genes often reflect technical bias of the scRNA-seq experiments, such as the survival of cells, we also recommend excluding them from the scRNA-seq reference.

Additionally, as BayesPrism estimates the fraction of reads from each cell type, and malignant cells may systematically upregulate the ribosomal protein coding genes[10], the inclusion of these genes may result in an overestimation of the fraction of malignant cells by a multiplicative constant.

In addition to ribosomal genes, we also advise users to look out for highly expressed genes, such as MALAT1 and other nuclear lincRNAs, actin and hemoglobin, as recommended by some scRNA-seq processing pipelines. As minor differences in the expression level of these genes may cause global changes in normalization when constructing the reference matrix.

Supplementary Note 6: Robustness against an undersampled reference

In this supplementary note, we discuss the details of how an undersampled reference can affect the accuracy of deconvolution. An undersampled reference falls into one of the following three categories: 1) reference with one or few cell types missing, 2) reference with low numbers of cells, 3) reference with limited number of patients (in the tumor setting). We discuss how each of them may affect the accuracy of deconvolution.

Some cell types can be missing from the single cell reference due to the scarcity or low capture efficiency, hereafter referred to as “missing cell types”. In the absence of tumor heterogeneity, the expression of all cell types or a subset of missing cell types, can be recovered using matrix factorization-based approaches, such as NMF and LDA, given a large amount of bulk RNA-seq samples each containing different fractions of cell types and the presence of signature genes. In the presence of heterogeneous tumors, the *de novo* modeling of the expression of missing cell types becomes an unidentifiable problem. In practice, the expression of missing cells will be redistributed into the reference cells by all deconvolution methods, according to the similarity with respect to each reference cell type. We therefore empirically tested how missing cell types may possibly affect BayesPrism by excluding T cell, a rare cell type in GBM, from the refGBM8 reference, and tested the performance in deconvolving the GBM28 pseudo-bulks (N=1,350) containing T cells. We found that the cell type-level correlations are robust up to 40-50% of missing T cells (**Fig. SN5**, **left**). The MSE, however, goes up quickly as the missing fraction, indicating that the missing cell fraction becomes redistributed into other cell types (**Fig. SN5**, **right**). Macrophages are the most affected, due to its higher similarity in expression with T cells. Nevertheless, this is less problematic for references of missing cell types due to scarcity in the tissue. For instance, T cells are present at < 5% in both GBM scRNA-seq datasets, at which level the deconvolution is not affected. Subsetting on genes that are not (or less) expressed in the missing cell types can also ameliorate this issue.

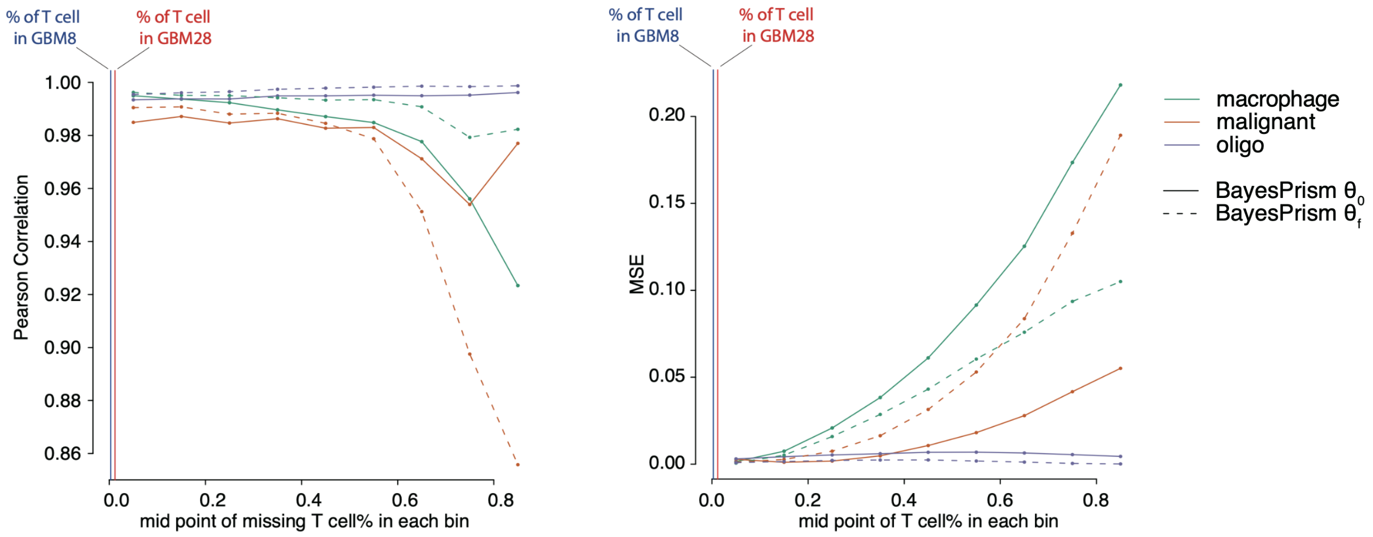

Fig. SN5 | BayesPrism is robust to missing cell types in the reference. Line plots show the cell type-level Pearson's correlation coefficient (left) and MSE (right) for the deconvolution of simulated GBM28 (N=1350) using refGBM8 with T cells removed as the reference. The X axis marks the midpoints of each 10% width bin. Lines are colored by cell type. Solid lines represent θ_0, while dashed lines represent θ_f. Vertical bars mark the average observed T cell fraction in GBM8 and GBM28.

Number of cells in the scRNA-seq reference also affects the deconvolution. The extent to which this affects accuracy depends on the cell types to be inferred and the sequencing depth of the single cell platforms. As a rule of thumb, to resolve highly similar cell types, e.g. subtypes of T cells, larger numbers of cells (and hence more information) are needed. The higher the heterogeneity the tumor is, the more patients are needed. The shallower the sequencing depth, e.g. 10X vs SMART-seq2, the greater the numbers of cells are needed. It is impossible to determine the minimum number of cells for each task a priori. Therefore, we recommend to run a leave-one-out test to estimate the actual performance. To estimate the ballpark, we downsampled the cells in each cell type to a particular size N (without replacement). As the number of cells in the scRNA-seq reference vary between cell types, we are only allowed to downsample cell types with a higher number of cells than N. Therefore, N represents the maximum number of cells of all cell types. BayesPrism approaches its optimal performance when the maximum number of cell types contain between 100 (for SMART-seq2) to 500 cells (for platforms with depth close to 10X) (**Fig. SN6**). Taken together, our analyses suggest that BayesPrism is generally robust to reference with undersampled cell numbers.

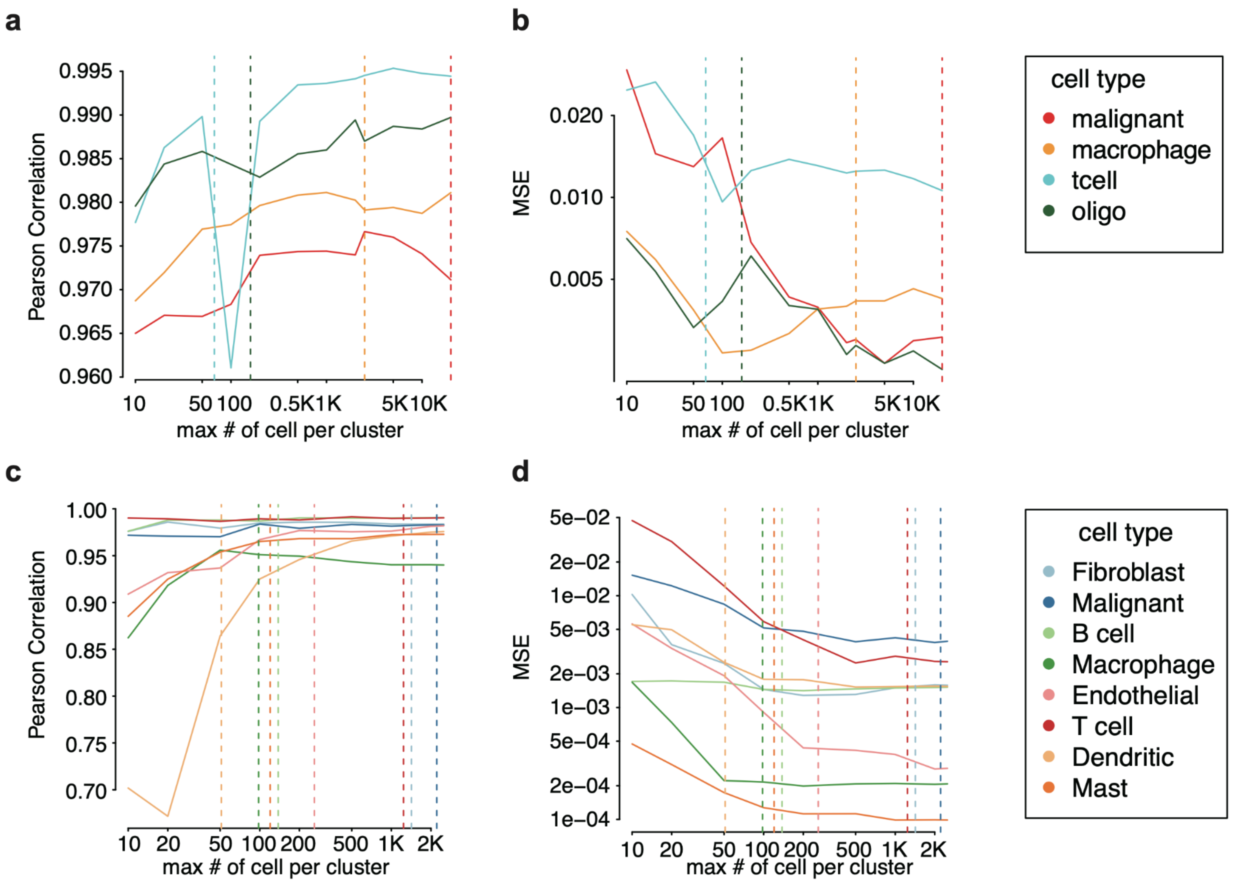

Fig. SN6 | BayesPrism is robust to a downsampled reference scRNA-seq dataset. Line plots show the cell type-level Pearson's correlation coefficient (left) and MSE (right) for the deconvolution of simulated GBM28 (N=1350) by refGBM8 (**a** and **b**) and HNSCC leave-one-out test using reference with downsampled single cells (**c** and **d**). The X axis marks the maximum number of cells in each cell type (or cell states of malignant cells) in the reference. Lines are colored by cell type. Dashed vertical lines mark the observed number of cells in each cell type in the original reference.

In the case of tumor deconvolution, the number of patients / number of tumor sub-clusters in the reference may also affect the accuracy. The extent to which this affects accuracy depends on the heterogeneity of the tumor of interest. We recommend planning the experiments by running a leave-one-out test to estimate the number of tumor patients needed. It is often ideal to have a scRNA-seq reference that represents all major tumor cell states or subtypes. To test empirically, we subsampled the malignant cells from individual patients from the 8 GBM scRNA-seq reference dataset. BayesPrism achieved saturating levels of performance deconvolving GBM28 pseudo-bulk data with as few as 3-4 patients (Fig. SN7). Taken together, the analysis suggests that BayesPrism can account for tumor heterogeneity with relatively small numbers of cancer patients.

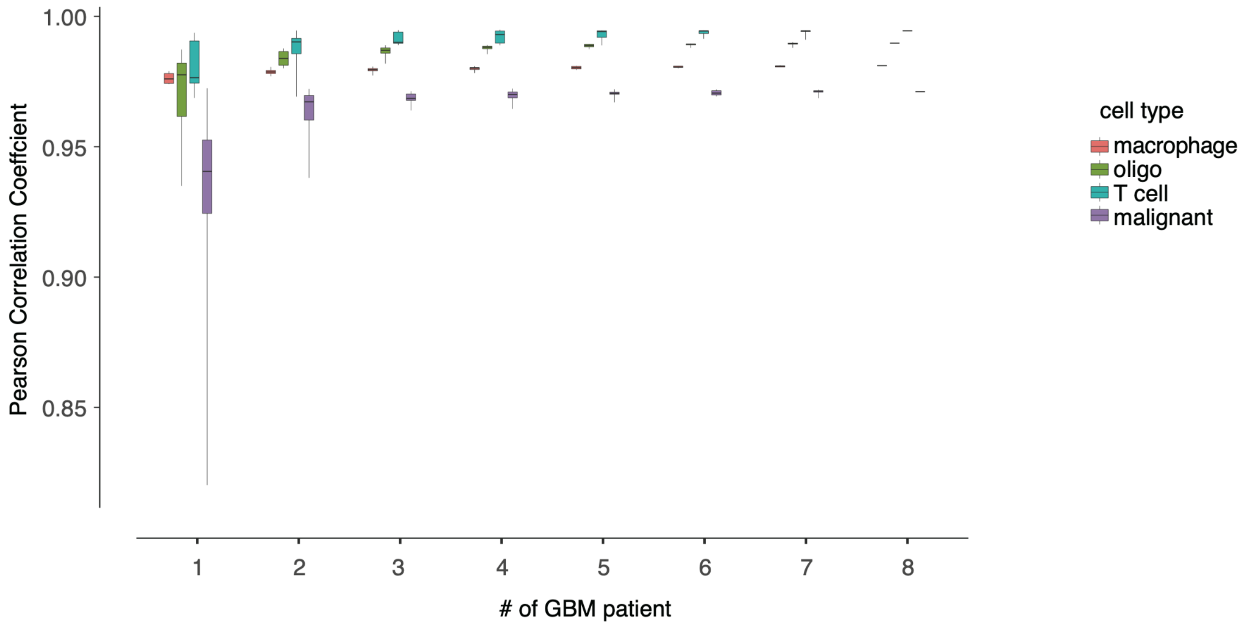

Fig. SN7 | BayesPrism is robust to under-represented heterogeneity in the reference scRNA-seq dataset. Boxplots show the distribution of cell type-level Pearson's correlation coefficient as a function of the number of downsampled patients in refGBM8, in which malignant cells are excluded from patients that are not sampled. Boxes mark the 25th percentile (bottom of box), median (central bar), and 75th percentile (top of box). Whiskers represent extreme values within 1.5 fold of the inter quartile range. n=8 randomly selected combinations of patients if the number of GBM patients is between 1 and 7, and n=1 if the number of GBM patients is 8.

Supplementary Note 7: Rich correlation structure between stromal cells

To determine how non-malignant cell types co-varied with each other, we examined the pairwise correlations between each cell type in the TCGA cohort (**Fig. SN8**). In GBM, pericytes and endothelial cells showed strong correlation (Spearman's rank correlation [ρ] = 0.34), consistent with their combined presence in vascular structures[11]. Macrophages also show strong correlation with pericytes in GBM ([ρ] = 0.43), potentially driven by hypoxia-induced necrosis. However, correlations were weaker overall in GBM than in other cancer types. In HNSCC, the proportion of immune cell types were highly correlated with each other. We also noted a high correlation between most immune cell types and endothelial cells. In melanoma, endothelial cells had a relatively strong positive association with fibroblasts (ρ = 0.62), which may be consistent with reports that fibroblast ECM remodelling promotes angiogenesis in melanoma[12]. We also noted two separate submodules of highly correlated immune cells, one consisting of CD4+ T-cells and B cells, and the other consisting of CD8+ T-cells and macrophages. This finding suggests that melanoma patients may have two distinct types of immune response, mediated by humoral and cellular immunity, to varying degrees between patients.

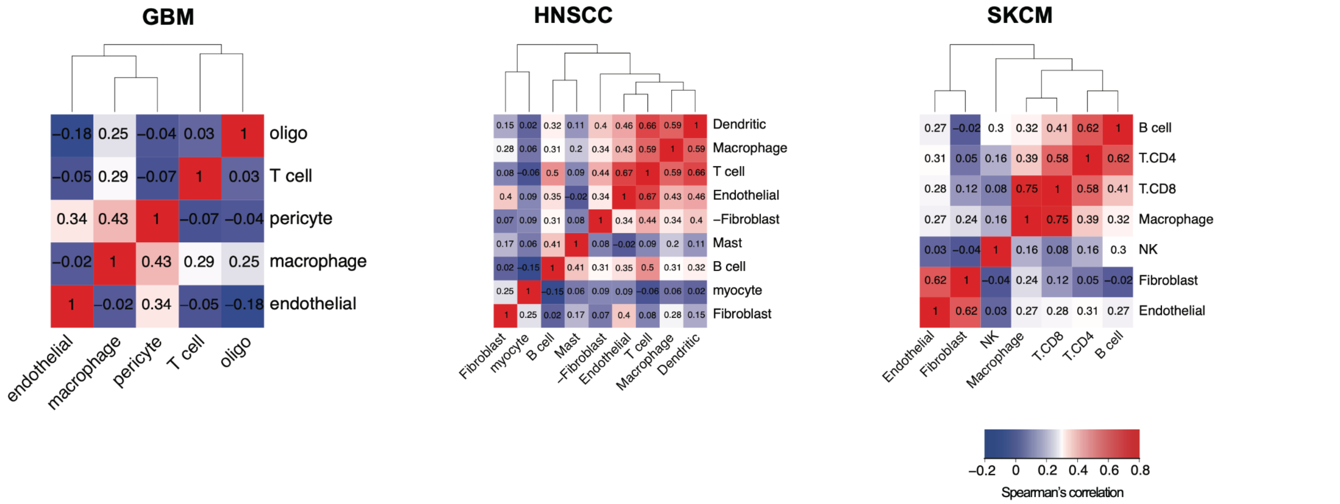

Fig. SN8 | Heatmaps show the Spearman's rank correlation between non-malignant cells in each tumor type.

Supplementary Note 8:
Validating correlations using marker genes from query cell types.

Discovering genes involved in tumor-microenvironment interactions is a major unresolved problem. Discovering candidate interactions in which gene expression in malignant cells is correlated with fractions of non-malignant cells is a potentially powerful strategy that has identified strong candidates which have been validated using single-cell RNA-seq studies. However, single cell RNA-seq studies are underpowered to discover these associations due to small sample size, providing a motivation to use the large quantities of existing bulk-RNA-seq. Directly correlating the fraction of each query cell type and the bulk tumor expression without deconvolution will result in false positive correlations from genes that are highly expressed by the query cell type. On the other extreme, regressing the fraction of query cell type out of bulk expression will cause the expression to be trivially uncorrelated with the fraction of the query cell type.

We therefore examined correlations between gene expression in malignant cells estimated by BayesPrism and the proportion of each query cell type. We reasoned that BayesPrism recovered malignant cell expression accurately enough to reduce this type of false positive. To test this hypothesis, we examined the distribution of marker genes that were specifically expressed in each cell type based on independently derived data. Whereas marker genes had systematically higher correlations in the bulk data, these were reduced to a median near 0 when using the estimates of expression in malignant cells produced by BayesPrism (**Fig. SN9**). This suggests that BayesPrism expression estimates are effective in removing many trivial false positives.

Details of this analysis are as follows. We computed the Pearson's correlation coefficient between the variance-stabilized transformed expression over a set of marker genes with the cell type fractions. Marker genes for CD4+ and CD8+ T cell and monocytes were derived from the LM6 matrix from the CIBERSORT website (https://cibersort.stanford.edu/download.php), which were based on GSE60424[13], by assigning each gene to the cell type with the maximum expression value. Markers for oligodendrocytes, endothelial cells, pericytes, and microglias in normal brains were derived from the gene list generated by Lake et al. using normal brain snDrop-seq[14]. Only marker genes uniquely assigned to each cell type were used for the plot.

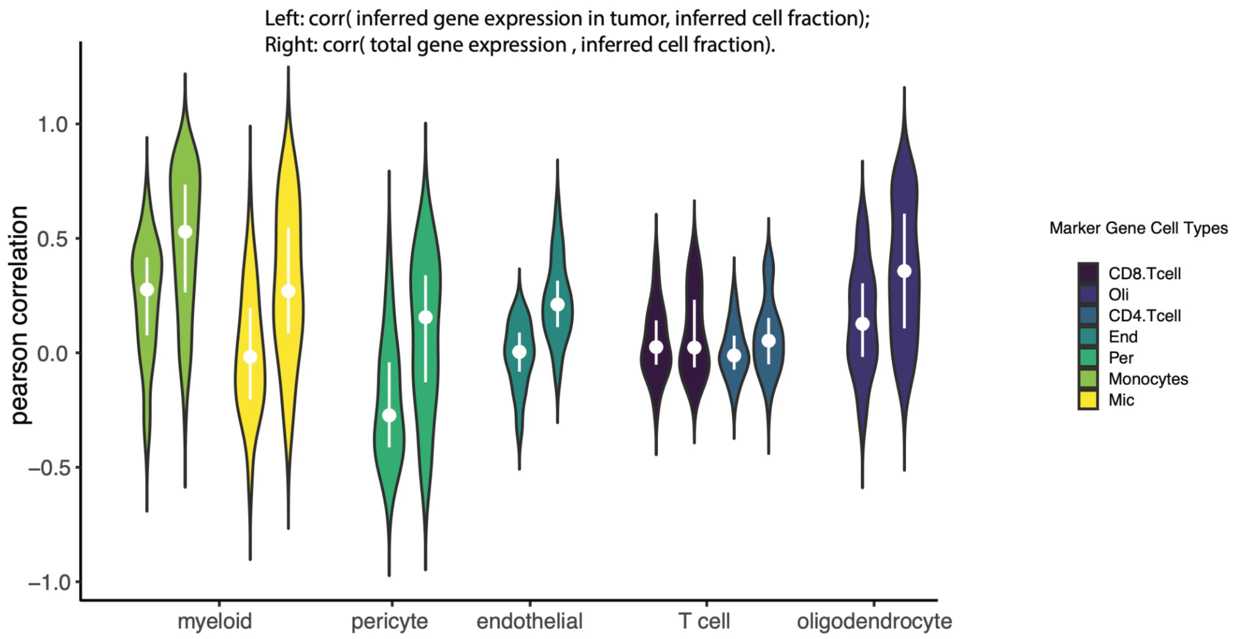

Fig. SN9 | BayesPrism removes false positive correlations from cell type marker genes. Violin plot shows the distribution of Pearson's correlation between gene expression in malignant cells inferred by BayesPrism (left violins) or total gene expression of bulk RNA-seq (right violins) and BayesPrism predicted fractions of each cell type on their corresponding marker genes over TCGA-GBM. Median correlations are shown by white dots and upper/lower quartiles are shown by bars. Braces on the horizontal direction label the cell type fraction on which the correlations were computed. Color indicates the cell type of which the marker genes are curated from independent datasets.

Supplementary Note 9: Defining Malignant Intrinsic Correlative Genes

When computing the correlation between the inferred gene expression in malignant cells and the proportion of non-malignant query cell types in **Fig. 3a**, there is a concern that some genes might falsely show high correlation due to the expression in the query cell type. This can happen when cell states of the query cell types in the bulk are not comprehensively represented by the scRNA-seq, due to the heterogeneity of query cell types. To filter out false correlations and enrich for intrinsic genes expressed by malignant cells for the gene set enrichment analysis, we developed two filters: (1) the *malignant intrinsic filter* that selects genes significantly higher expressed in at least one malignant cell state compared to all non-malignant cell types / states based on the scRNA-seq reference, and (2) the *regress-out filter* that selects genes that show association with the malignant cells unexplained by the expression of the query cell types. Users can customize the selection of filters and their cutoffs based on the purpose of their analysis to trade off false positives against false negatives. Details of these filters are described as follows.

The malignant intrinsic filter: To start with, we first normalized the raw count in each single cell using scran. This is only done for datasets refGBM8 and scSKCM, which are on the raw count scale. We then performed log2(Y+0.1) for refGBM8, log2(TPM+10) for scHNSCC, and log2(Y+1) for scSKCM, with Y being the normalized count. We used a one-sided t test (higher expression in malignant cells), and then for each malignant cell state we take the maximum p value between it and every other non-malignant cell state (refGBM8) / cell type (scSKCM and scHNSCC). We tested differential expression at the cell type level for scSKCM and scHNSCC, because SMART-seq2 has significantly fewer cell types than 10x / microwell-based platforms, which would reduce statistical power if the test was performed on the level of cell states. Lastly, we selected the gene if the maximum p value is less than 0.01 in at least one malignant cell state.

The regress-out filter: In the case of incomplete representation of non-malignant query cell states in the scRNA-seq reference, portions of reads from genes expressed higher in the non-malignant query cells may also get assigned to the malignant cells and over all non-malignant cell types, potentially causing false positive associations between the inferred expression in malignant cells and the proportion of query cell types for those genes. Likewise, it can also cause false negative associations for missing cell states with lower expression in the scRNA-seq reference. To account for these technical biases, we may take the advantage of the Bayesian property of BayesPrism, in which the estimates of gene expression of one cell type will drift from the prior (driven by scRNA-seq) to the posterior (driven by bulk) as the cell type fraction increases. We developed a statistical framework by performing a likelihood ratio test on the null hypothesis of $\beta_{0(t,g)}=0$ in the linear model:

$$\theta_t \sim \beta_{0(t,g)} \cdot \log_2(Y_{(mal,g)}) + \beta_{1(t,g)} \cdot \log_2(Y_{(t,g)}) \qquad (1)$$

where each random variable has N observations (N=number of bulk samples).

θ_t is the proportion of query cell type t. For each n, $Y_{n(mal,g)}$ is $Z_{n(mal,g)}$ normalized by the norm.to.one function, such that, $\sum_g Y_{n(mal,g)}=1$, and $\min(Y_{n(mal,g)})=10^{-8}$ (the default pseudo.min) for all n. Likewise $Y_{(t,g)}$ is the normalized expression value for non-malignant query cells. In theory, when the query cell type is presented with non-zero fractions, we can directly apply the norm.to.one function on $Z_{n(t,g)}$ to obtain $Y_{n(t,g)}$. However, unlike malignant cells, non-malignant cells can be absent or presented in an extremely low fraction. In those cases, $Z_{n(t,g)}$ will be close to zero, and hence directly normalizing $Z_{n(t,g)}$ to get $Y_{n(t,g)}$ will yield unstable estimates. To

circumvent this, we developed an MAP estimator for $Y_{n(t,g)}$ by modeling a prior distribution over $Y_{n(t,g)}$ as follows.

$$Y_{n(t,\cdot)} \sim \text{logit normal}\ (\mu_t, \sigma^2_t),$$

where $\mu_t \in \mathbb{R}^G$ and $\sigma^2_t \in \mathbb{R}^G$, and σ^2_t are the entries of the diagonal covariance matrix;

$$Z_{n(t,\cdot)} \sim \text{multinomial}\ (Y_{n(t,\cdot)}, R_{n,t}), \text{ where } R_{n,t} = \sum_g Z_{n(t,g)}$$

μ_t and σ^2_t are hyper-parameters of the model, fitted using a subset of samples index by the set $N_{0(t)}$, in which $Y_{n(t,\cdot)}$ has higher Spearman correlation with the posterior sum $\sum_n Y_{n(t,\cdot)}$ than the prior φ_t . This strategy aims to fit μ_t and σ^2_t using samples with higher fractions of the query cell type that circumvents the need to manually define a cutoff, since samples with higher cell proportion tend to be more similar to the posterior than to the prior. We then set

$$\mu_{t,g} = \log\left(\sum_{n \in N_{0(t)}} Z_{n(t,g)} \Big/ \sum_{g,\, n \in N_{0(t)}} Z_{n(t,g)} \right),$$

$$\text{and } \sigma^2_{t,g} = \text{Var}(\log(\text{norm.to.one}(Z_{n(t,g)}))), \text{ for } n \in N_{0(t)},$$

where Var denotes the sample variance. As there is no closed form solution, we used the L-BFGS-B optimizer[15] to find the MAP of $Y_{n(t,g)}$.

To visualize the effect of these two filters on correlative genes, we computed the distribution of mean of z scores of expression over a set of correlative genes (with Spearman's correlation coefficient greater than 0.25) from the corresponding scRNA-seq dataset of the corresponding tumor types, and observed that correlative genes showed higher expression in tumor cells (**Fig. SN10**).

a

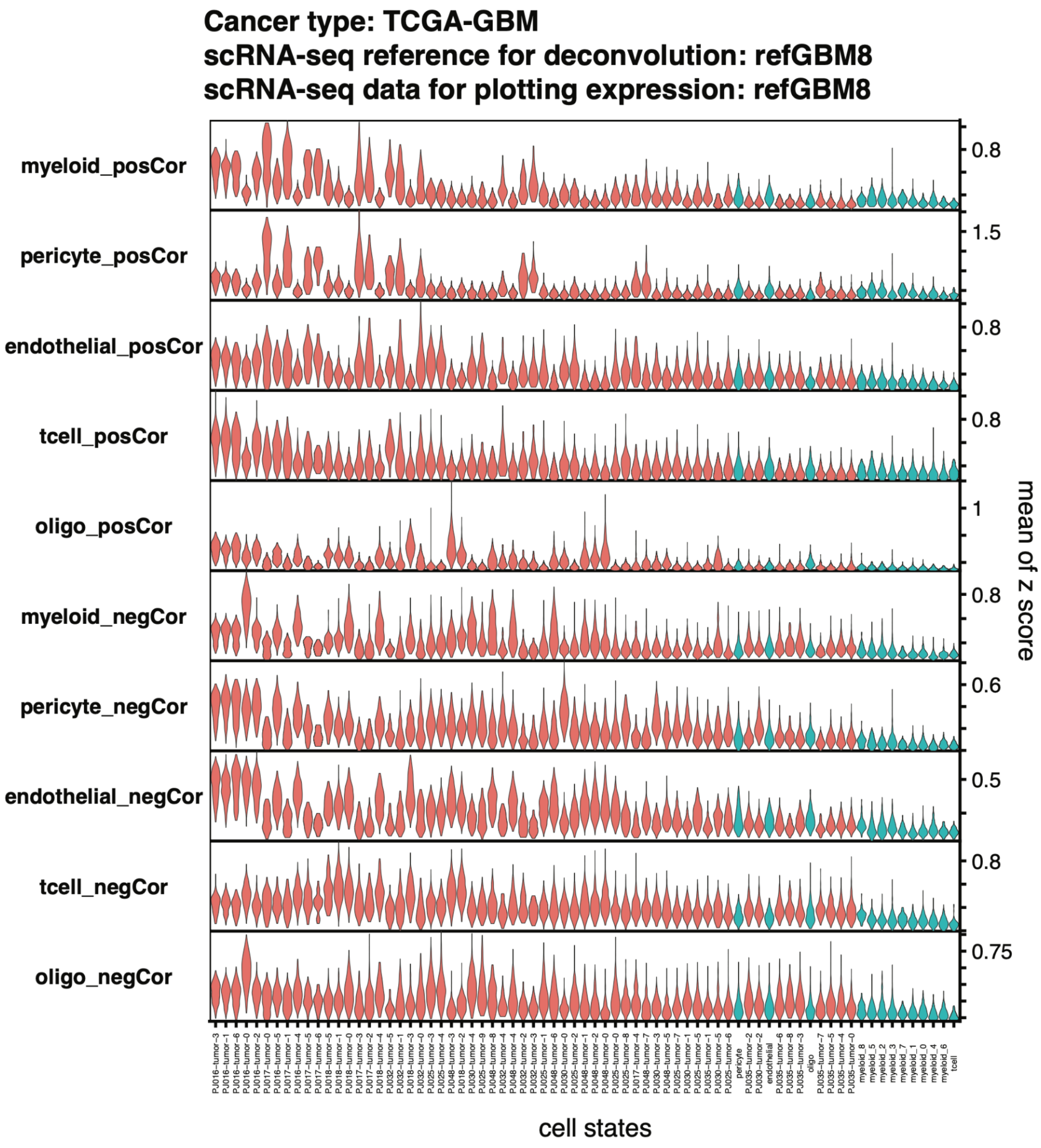

Cancer type: TCGA-GBM
scRNA-seq reference for deconvolution: refGBM8
scRNA-seq data for plotting expression: refGBM8
myeloid_posCor
pericyte_posCor
endothelial_posCor
tcell_posCor
oligo_posCor
myeloid_negCor
pericyte_negCor
endothelial_negCor
tcell_negCor
oligo_negCor
0.8
1.5
0.8
0.8
1
0.8
0.6
0.5
0.8
0.75
mean of z score
cell states

b

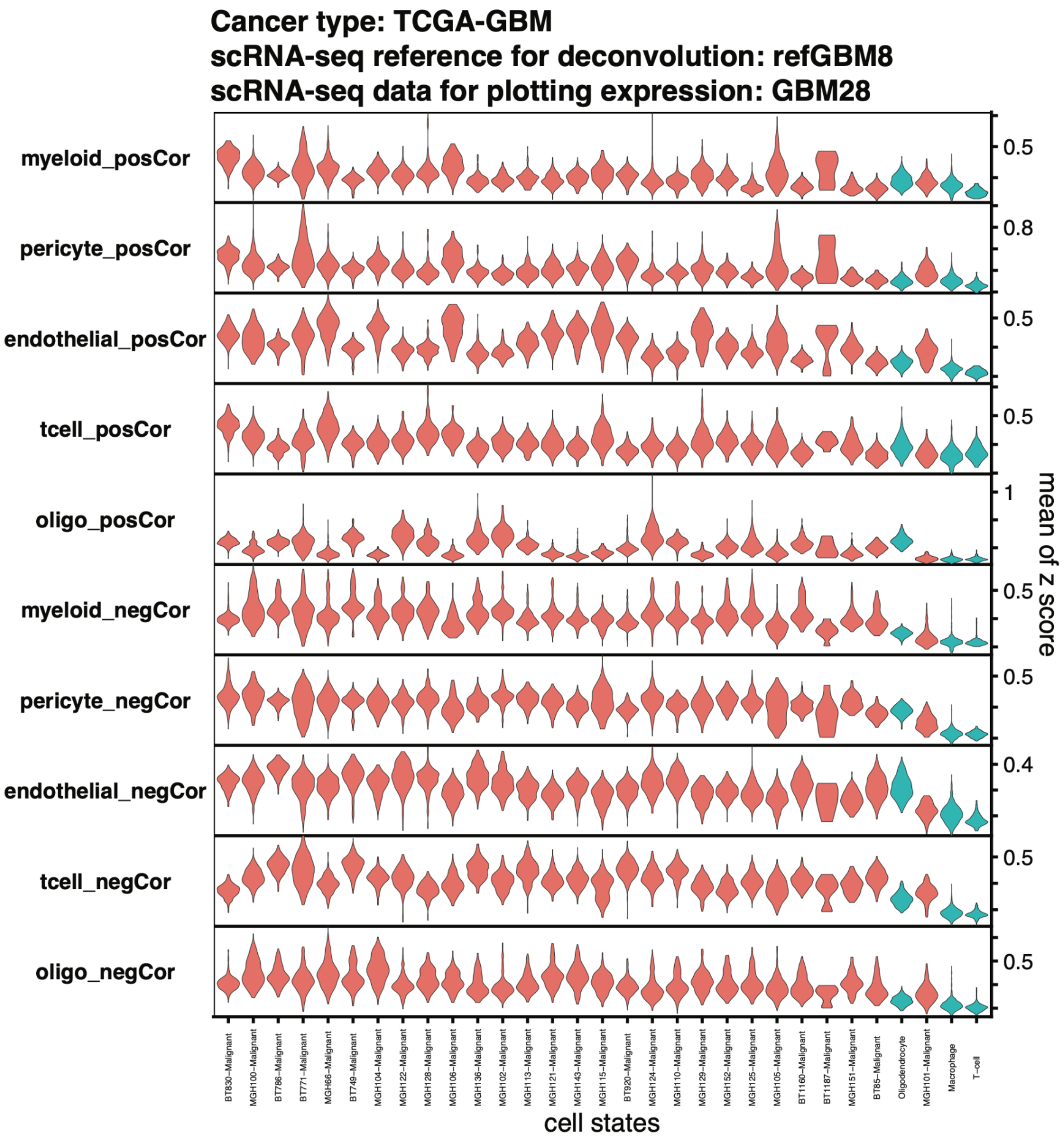

Cancer type: TCGA-GBM
scRNA-seq reference for deconvolution: refGBM8
scRNA-seq data for plotting expression: GBM28
myeloid_posCor
pericyte_posCor
endothelial_posCor
tcell_posCor
oligo_posCor
myeloid_negCor
pericyte_negCor
endothelial_negCor
tcell_negCor
oligo_negCor
0.5
0.8
0.5
0.5
1
0.5
0.5
0.4
0.5
0.5
mean of z score
BT830–Malignant
MGH100–Malignant
BT786–Malignant
BT771–Malignant
MGH66–Malignant
BT749–Malignant
MGH104–Malignant
MGH122–Malignant
MGH128–Malignant
MGH106–Malignant
MGH136–Malignant
MGH102–Malignant
MGH113–Malignant
MGH121–Malignant
MGH143–Malignant
MGH115–Malignant
BT920–Malignant
MGH124–Malignant
MGH110–Malignant
MGH129–Malignant
MGH152–Malignant
MGH125–Malignant
MGH105–Malignant
BT1160–Malignant
BT1187–Malignant
MGH151–Malignant
BT85–Malignant
Oligodendrocyte
MGH101–Malignant
Macrophage
T–cell
cell states

c

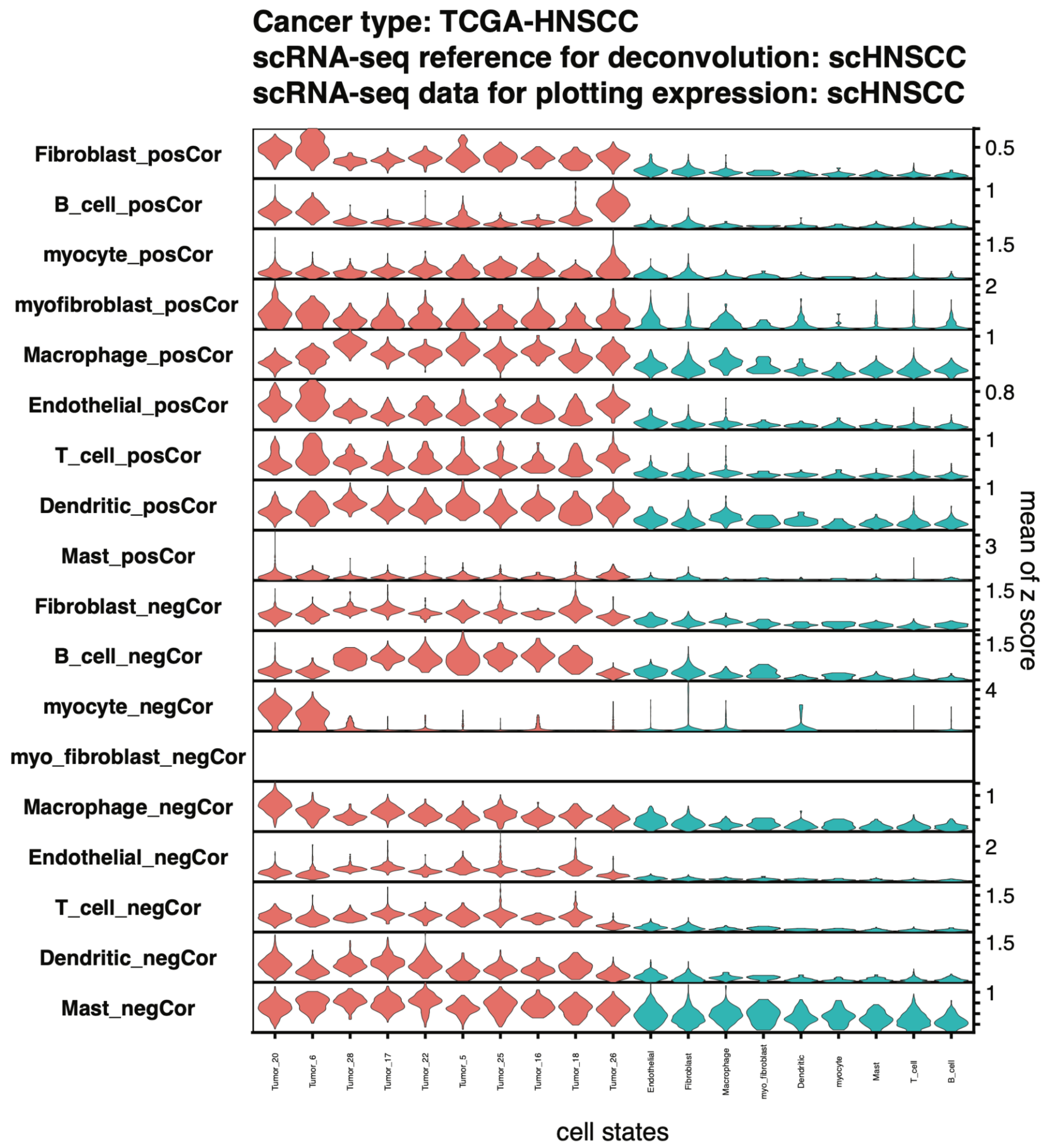
Cancer type: TCGA-HNSCC
scRNA-seq reference for deconvolution: scHNSCC
scRNA-seq data for plotting expression: scHNSCC
Fibroblast_posCor
B_cell_posCor
myocyte_posCor
myofibroblast_posCor
Macrophage_posCor
Endothelial_posCor
T_cell_posCor
Dendritic_posCor
Mast_posCor
Fibroblast_negCor
B_cell_negCor
myocyte_negCor
myo_fibroblast_negCor
Macrophage_negCor
Endothelial_negCor
T_cell_negCor
Dendritic_negCor
Mast_negCor
mean of z score
Tumor_20
Tumor_6
Tumor_28
Tumor_17
Tumor_22
Tumor_5
Tumor_25
Tumor_16
Tumor_18
Tumor_26
Endothelial
Fibroblast
Macrophage
myo_fibroblast
Dendritic
myocyte
Mast
T_cell
B_cell
cell states

d

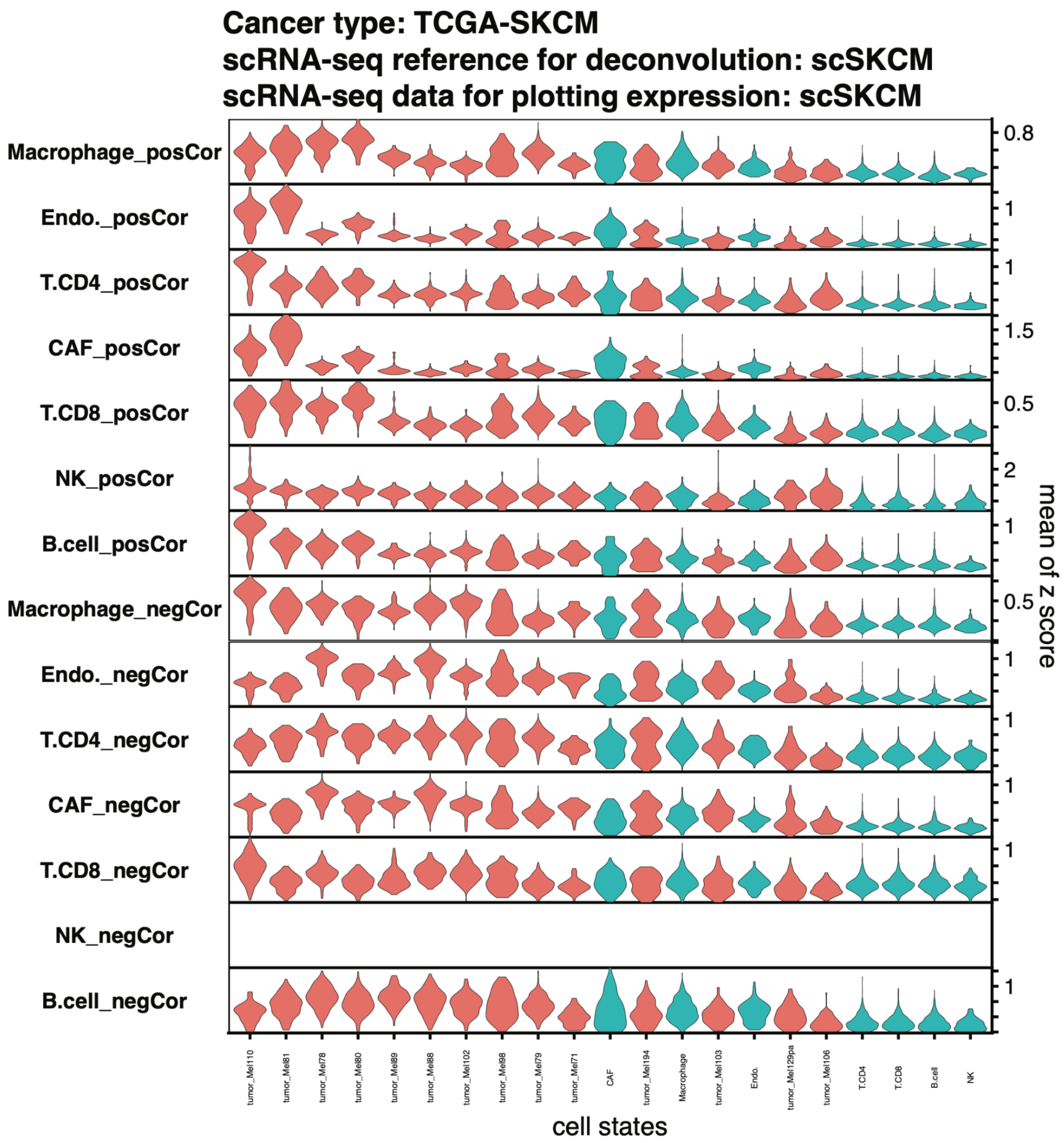

Fig. SN10 | Violin plots show the mean of z scores of expression over a gene set of interest (each row) computed using a particular scRNA-seq dataset. Each row defines a set of genes that passed both the tumor intrinsic filter and the regress-out filter, and also correlated with the cell type fraction of a non-malignant cell type. Each column denotes a cell state or cell type corresponding to the tumor intrinsic filter.

Supplementary Note 10:
Gene set enrichment analysis and differential gene expression analysis provide mechanistic insights into how each program affects clinical outcomes

The GSVA for subtype marker genes and MgSigDB biological process for each gene program inferred from TCGA-GBM using the embedding learning module of BayesPrism was shown in **Fig. SN11a**. Top differentially expressed genes between bulk samples associated with each program were shown in **Fig. SN12a**. In GBM, program 4, a program positively correlated with macrophages, pericytes and endothelial cells (**Fig. 4e**), was associated with EMT, TNF-α signaling via NF-κB, IL-6/JAK/STAT3 signaling, hypoxia, angiogenesis and inflammatory responses. Program 5 was negatively correlated with macrophages (**Fig. 4e**), had the highest cell cycle score, and was strongly enriched for the gene ontology terms related to cell replication. Program 3 was associated with core cellular processes, such as regulation of nucleobase containing compound transport, neuroblast proliferation, mRNA processing, DNA conformational changes, and had a high cell cycle score. We also noted some interferon signaling associations in program 3. Overall, the associations we observed between gene programs 3-5 and biological processes frequently mirrored those reported previously[16]. Programs 1 and 2 had some notable differences from all previously described subtypes. Program 1 was similar to both AC and MES, which agrees with the recently reported stratification of the MES subtype and heterogeneity of the AC subtype in GBM[17]. It was strongly enriched for genes associated with cell respiration, and was negatively associated with endothelial cells. Interestingly, program 2 was positively correlated with oligodendrocytes, was enriched for multiple neuronal processes, and had the lowest cell cycle score. As it constitutes on average 22% of the expression of malignant cells, it is unlikely that this gene program is due to contamination of normal brain tissue. On the contrary, it may reflect the quiescent neuroblast-like malignant populations identified by scRNA-seq[2].

Several of the gene programs in HNSCC and SKCM showed significant associations with patient survival. Notably, in HNSCC, Program 1 had a negative association with survival ($p = 0.017$, Wald test, **Fig. SN13a**). For instance, HNSCC Program 1 was uniquely enriched for the partial EMT program (p-EMT) identified by a recent cell study[18] (**Fig. 4f**). Comparing this program to subtypes derived from bulk TCGA tumor profiles, we found this program resembled the basal, but not the mesenchymal subtype derived from TCGA (**Fig. SN11b**), consistent with the characterization of the p-EMT by the scRNA-seq study[18]. Top differentially upregulated genes that marked this program also overlap signature genes of p-EMT, including *MMP10*, *LAMA3*, *LAMC2*, *COL17A1*, and *SEMA3C* (**Fig. SN12b**, **Supplementary Table 5b**). Additionally, this program was positively correlated with fibroblasts (**Fig. 4f**), which was also concordant with the previous finding by immunohistochemistry[18].

In SKCM, multiple gene programs enriched for AXL and MITF markers were correlated with survival (**Fig. 4h-j**, **Fig. SN13b**). Survival-associated gene programs in SKCM had enrichment or depletion for the AXL and MITF gene programs identified by the TCGA bulk analyses, as well as the T cell exclusion programs identified by the scRNA-seq study (**Fig. 4g**). Program 2 showed a high AXL score, and was enriched for EMT, NF-κB, and hypoxia, and depleted of cell cycle genes (**Fig. 4g**, **Fig. SN11c**). This program represents a less replicative malignant state due to environmental stress, and was positively correlated with survival (**Fig. 4h**). Programs 3 and 4 were positively and negatively correlated with both immune cells and survival (**Fig. 4g,i,j**), coinciding with the relationship between immune infiltration and survival in SKCM (**Fig. 2b-c**). Program 3 was enriched for immune process and interferon response, and genes repressed in the T cell exclusion program, including *C4A* and *IFI27* (**Fig. SN12c**,

Supplementary Table 5c), while program 4 was enriched for Myc targets and genes induced in the T cell exclusion program (**Fig. 4g**, **Fig. SN11c**).

Several of the gene programs in HNSCC and SKCM showed significant associations with patient survival. Notably, in HNSCC, Program 1 had a negative association with survival (p = 0.017, Wald test, (**Fig. SN13a**). Program 1 was uniquely enriched for the partial EMT program (p-EMT) identified by the single cell study[18] (**Fig. 4f**). Comparing this program to subtypes derived from bulk TCGA tumor profiles, we found that it resembled the basal subtype but not the mesenchymal subtype (**Fig. SN11b**), consistent with the characterization of the p-EMT by the scRNA-seq study[18]. Top differentially upregulated genes that marked this program also overlap signature genes of p-EMT, including *MMP10*, *LAMA3*, *LAMC2*, *COL17A1*, and *SEMA3C* (**Fig. SN12b**, **Supplementary Table 5b**). Additionally, this program was positively correlated with fibroblasts (**Fig. 4f**), which was also concordant with the previous finding by immunohistochemistry[18]. Therefore, the negative association between HNSCC program 1 and survival discovered in our work most likely reflects the same biological processes that explains increased invasiveness of cells expressing p-EMT genes, as well as the positive correlation between p-EMT scores, metastasis, and other adverse pathological features (e.g., lymph node metastases and higher nodal stage and tumor grade)[18].

In SKCM, multiple gene programs correlated with survival (**Fig. 4h-j**, **Fig. SN13b**). Survival-associated gene programs in SKCM had enrichment or depletion for the AXL and MITF gene programs identified by the TCGA bulk analyses, as well as the T cell exclusion programs identified by the scRNA-seq study (**Fig. 4g**). Program 1 and 2 showed the highest MITF and AXL enrichment scores, and were negatively and positively correlated with fibroblasts respectively, which was consistent with the previous analysis on the TCGA dataset based on correlations on marker genes[19]. Program 2 showed a high AXL score, and was enriched for EMT, NF-κB, and hypoxia, and depleted of cell cycle genes (**Fig. 4g**, **Fig. SN11c**). This program represents a less replicative malignant state due to environmental stress, and was positively correlated with survival (**Fig. 4h**). Programs 3 and 4 were positively and negatively correlated with both immune cells and survival (**Fig. 4g,i,j**), coinciding with the relationship between immune infiltration and survival in SKCM (**Fig. 2b-c**). Program 3 was enriched for immune process and interferon response, and genes repressed in the T cell exclusion program, including *C4A* and *IFI27*, while program 4 was enriched for Myc targets and genes induced in the T cell exclusion program (**Fig. SN11c**, **Fig. SN12c**, **Supplementary Table 5c**).

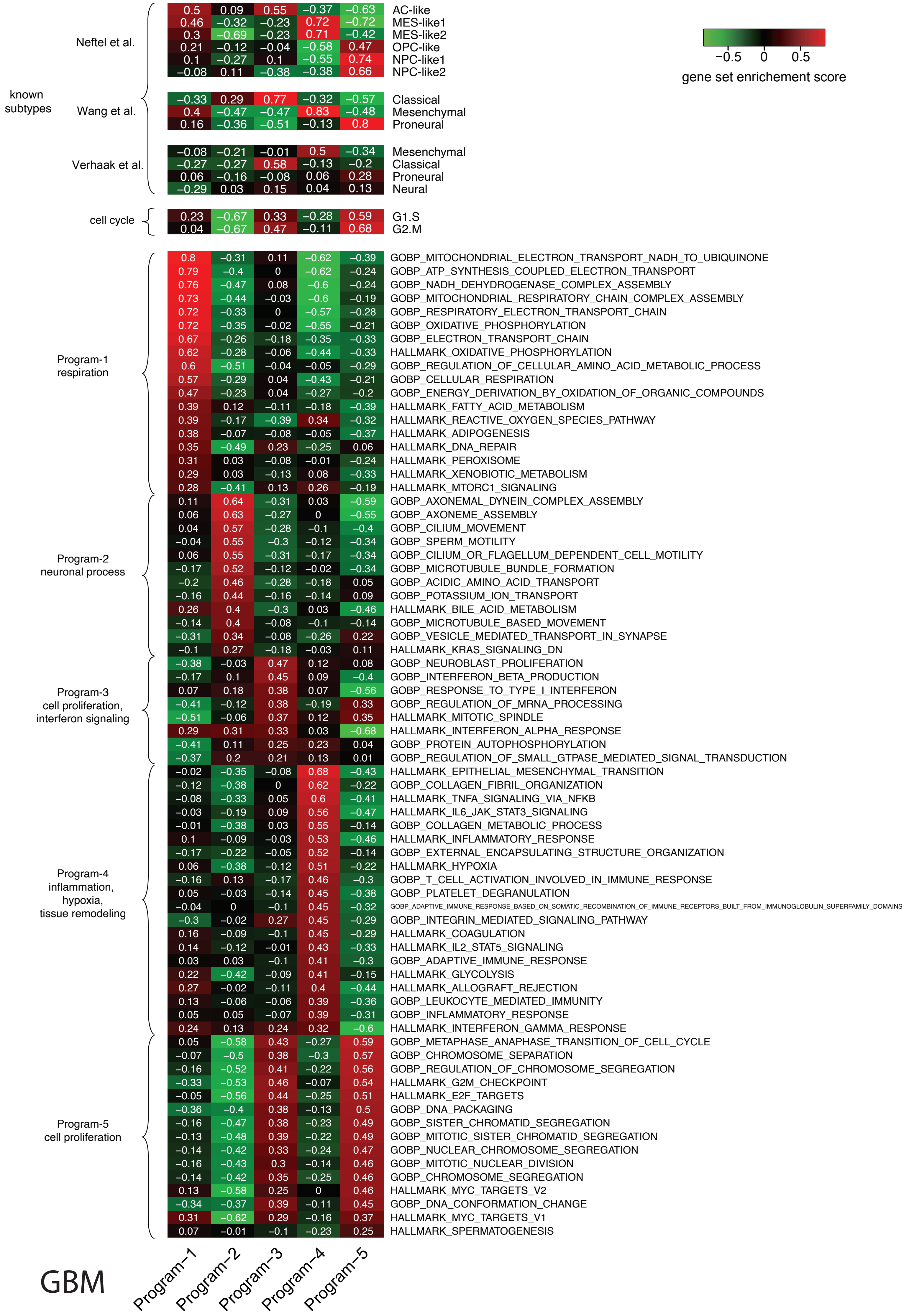

gene set enrichement score
−0.5 0 0.5
known subtypes
Neftel et al.
0.5 0.09 0.55 −0.37 −0.63 AC-like
0.46 −0.32 −0.23 0.72 −0.72 MES-like1
0.3 −0.69 −0.23 0.71 −0.42 MES-like2
0.21 −0.12 −0.04 −0.58 0.47 OPC-like
0.1 −0.27 0.1 −0.55 0.74 NPC-like1
−0.08 0.11 −0.38 −0.38 0.66 NPC-like2
Wang et al.
−0.33 0.29 0.77 −0.32 −0.57 Classical
0.4 −0.47 −0.47 0.83 −0.48 Mesenchymal
0.16 −0.36 −0.51 −0.13 0.8 Proneural
Verhaak et al.
−0.08 −0.21 −0.01 0.5 −0.34 Mesenchymal
−0.27 −0.27 0.58 −0.13 −0.2 Classical
0.06 −0.16 −0.08 0.06 0.28 Proneural
−0.29 0.03 0.15 0.04 0.13 Neural
cell cycle
0.23 −0.67 0.33 −0.28 0.59 G1.S
0.04 −0.67 0.47 −0.11 0.68 G2.M
Program-1 respiration
0.8 −0.31 0.11 −0.62 −0.39 GOBP_MITOCHONDRIAL_ELECTRON_TRANSPORT_NADH_TO_UBIQUINONE
0.79 −0.4 0 −0.62 −0.24 GOBP_ATP_SYNTHESIS_COUPLED_ELECTRON_TRANSPORT
0.76 −0.47 0.08 −0.6 −0.24 GOBP_NADH_DEHYDROGENASE_COMPLEX_ASSEMBLY
0.73 −0.44 −0.03 −0.6 −0.19 GOBP_MITOCHONDRIAL_RESPIRATORY_CHAIN_COMPLEX_ASSEMBLY
0.72 −0.33 0 −0.57 −0.28 GOBP_RESPIRATORY_ELECTRON_TRANSPORT_CHAIN
0.72 −0.35 −0.02 −0.55 −0.21 GOBP_OXIDATIVE_PHOSPHORYLATION
0.67 −0.26 −0.18 −0.35 −0.33 GOBP_ELECTRON_TRANSPORT_CHAIN
0.62 −0.28 −0.06 −0.44 −0.33 HALLMARK_OXIDATIVE_PHOSPHORYLATION
0.6 −0.51 −0.04 −0.05 −0.29 GOBP_REGULATION_OF_CELLULAR_AMINO_ACID_METABOLIC_PROCESS
0.57 −0.29 0.04 −0.43 −0.21 GOBP_CELLULAR_RESPIRATION
0.47 −0.23 0.04 −0.27 −0.2 GOBP_ENERGY_DERIVATION_BY_OXIDATION_OF_ORGANIC_COMPOUNDS
0.39 0.12 −0.11 −0.18 −0.39 HALLMARK_FATTY_ACID_METABOLISM
0.39 −0.17 −0.39 0.34 −0.32 HALLMARK_REACTIVE_OXYGEN_SPECIES_PATHWAY
0.38 −0.07 −0.08 −0.05 −0.37 HALLMARK_ADIPOGENESIS
0.35 −0.49 0.23 −0.25 0.06 HALLMARK_DNA_REPAIR
0.31 0.03 −0.08 −0.01 −0.24 HALLMARK_PEROXISOME
0.29 0.03 −0.13 0.08 −0.33 HALLMARK_XENOBIOTIC_METABOLISM
0.28 −0.41 0.13 0.26 −0.19 HALLMARK_MTORC1_SIGNALING
Program-2 neuronal process
0.11 0.64 −0.31 0.03 −0.59 GOBP_AXONEMAL_DYNEIN_COMPLEX_ASSEMBLY
0.06 0.63 −0.27 0 −0.55 GOBP_AXONEME_ASSEMBLY
0.04 0.57 −0.28 −0.1 −0.4 GOBP_CILIUM_MOVEMENT
−0.04 0.55 −0.3 −0.12 −0.34 GOBP_SPERM_MOTILITY
0.06 0.55 −0.31 −0.17 −0.34 GOBP_CILIUM_OR_FLAGELLUM_DEPENDENT_CELL_MOTILITY
−0.17 0.52 −0.12 −0.02 −0.34 GOBP_MICROTUBULE_BUNDLE_FORMATION
−0.2 0.46 −0.28 −0.18 0.05 GOBP_ACIDIC_AMINO_ACID_TRANSPORT
−0.16 0.44 −0.16 −0.14 0.09 GOBP_POTASSIUM_ION_TRANSPORT
0.26 0.4 −0.3 0.03 −0.46 HALLMARK_BILE_ACID_METABOLISM
−0.14 0.4 −0.08 −0.1 −0.14 GOBP_MICROTUBULE_BASED_MOVEMENT
−0.31 0.34 −0.08 −0.26 0.22 GOBP_VESICLE_MEDIATED_TRANSPORT_IN_SYNAPSE
−0.1 0.27 −0.18 −0.03 0.11 HALLMARK_KRAS_SIGNALING_DN
Program-3 cell proliferation, interferon signaling
−0.38 −0.03 0.47 0.12 0.08 GOBP_NEUROBLAST_PROLIFERATION
−0.17 0.1 0.45 0.09 −0.4 GOBP_INTERFERON_BETA_PRODUCTION
0.07 0.18 0.38 0.07 −0.56 GOBP_RESPONSE_TO_TYPE_I_INTERFERON
−0.41 −0.12 0.38 −0.19 0.33 GOBP_REGULATION_OF_MRNA_PROCESSING
−0.51 −0.06 0.37 0.12 0.35 HALLMARK_MITOTIC_SPINDLE
0.29 0.31 0.33 0.03 −0.68 HALLMARK_INTERFERON_ALPHA_RESPONSE
−0.41 0.11 0.25 0.23 0.04 GOBP_PROTEIN_AUTOPHOSPHORYLATION
−0.37 0.2 0.21 0.13 0.01 GOBP_REGULATION_OF_SMALL_GTPASE_MEDIATED_SIGNAL_TRANSDUCTION
Program-4 inflammation, hypoxia, tissue remodeling
−0.02 −0.35 −0.08 0.68 −0.43 HALLMARK_EPITHELIAL_MESENCHYMAL_TRANSITION
−0.12 −0.38 0 0.62 −0.22 GOBP_COLLAGEN_FIBRIL_ORGANIZATION
−0.08 −0.33 0.05 0.6 −0.41 HALLMARK_TNFA_SIGNALING_VIA_NFKB
−0.03 −0.19 0.09 0.56 −0.47 HALLMARK_IL6_JAK_STAT3_SIGNALING
−0.01 −0.38 0.03 0.55 −0.14 GOBP_COLLAGEN_METABOLIC_PROCESS
0.1 −0.09 −0.03 0.53 −0.46 HALLMARK_INFLAMMATORY_RESPONSE
−0.17 −0.22 −0.05 0.52 −0.14 GOBP_EXTERNAL_ENCAPSULATING_STRUCTURE_ORGANIZATION
0.06 −0.38 −0.12 0.51 −0.22 HALLMARK_HYPOXIA
−0.16 0.13 −0.17 0.46 −0.3 GOBP_T_CELL_ACTIVATION_INVOLVED_IN_IMMUNE_RESPONSE
0.05 −0.03 −0.14 0.45 −0.38 GOBP_PLATELET_DEGRANULATION
−0.04 0 −0.1 0.45 −0.32 GOBP_ADAPTIVE_IMMUNE_RESPONSE_BASED_ON_SOMATIC_RECOMBINATION_OF_IMMUNE_RECEPTORS_BUILT_FROM_IMMUNOGLOBULIN_SUPERFAMILY_DOMAINS
−0.3 −0.02 0.27 0.45 −0.29 GOBP_INTEGRIN_MEDIATED_SIGNALING_PATHWAY
0.16 −0.09 −0.1 0.45 −0.29 HALLMARK_COAGULATION
0.14 −0.12 −0.01 0.43 −0.33 HALLMARK_IL2_STAT5_SIGNALING
0.03 0.03 −0.1 0.41 −0.3 GOBP_ADAPTIVE_IMMUNE_RESPONSE
0.22 −0.42 −0.09 0.41 −0.15 HALLMARK_GLYCOLYSIS
0.27 −0.02 −0.11 0.4 −0.44 HALLMARK_ALLOGRAFT_REJECTION
0.13 −0.06 −0.06 0.39 −0.36 GOBP_LEUKOCYTE_MEDIATED_IMMUNITY
0.05 0.05 −0.07 0.39 −0.31 GOBP_INFLAMMATORY_RESPONSE
0.24 0.13 0.24 0.32 −0.6 HALLMARK_INTERFERON_GAMMA_RESPONSE
Program-5 cell proliferation
0.05 −0.58 0.43 −0.27 0.59 GOBP_METAPHASE_ANAPHASE_TRANSITION_OF_CELL_CYCLE
−0.07 −0.5 0.38 −0.3 0.57 GOBP_CHROMOSOME_SEPARATION
−0.16 −0.52 0.41 −0.22 0.56 GOBP_REGULATION_OF_CHROMOSOME_SEGREGATION
−0.33 −0.53 0.46 −0.07 0.54 HALLMARK_G2M_CHECKPOINT
−0.05 −0.56 0.44 −0.25 0.51 HALLMARK_E2F_TARGETS
−0.36 −0.4 0.38 −0.13 0.5 GOBP_DNA_PACKAGING
−0.16 −0.47 0.38 −0.23 0.49 GOBP_SISTER_CHROMATID_SEGREGATION
−0.13 −0.48 0.39 −0.22 0.49 GOBP_MITOTIC_SISTER_CHROMATID_SEGREGATION
−0.14 −0.42 0.33 −0.24 0.47 GOBP_NUCLEAR_CHROMOSOME_SEGREGATION
−0.16 −0.43 0.3 −0.14 0.46 GOBP_MITOTIC_NUCLEAR_DIVISION
−0.14 −0.42 0.35 −0.25 0.46 GOBP_CHROMOSOME_SEGREGATION
0.13 −0.58 0.25 0 0.46 HALLMARK_MYC_TARGETS_V2
−0.34 −0.37 0.39 −0.11 0.45 GOBP_DNA_CONFORMATION_CHANGE
0.31 −0.62 0.29 −0.16 0.37 HALLMARK_MYC_TARGETS_V1
0.07 −0.01 −0.1 −0.23 0.25 HALLMARK_SPERMATOGENESIS
GBM
Program−1
Program−2
Program−3
Program−4
Program−5

Group	Gene set	Program-1	Program-2	Program-3	Program-4
subtypes defined by bulk RNA-seq	Basal	0.51	−0.66	−0.38	0.56
	Mesenchymal	0.06	−0.35	0.26	0.28
	Atypical	−0.53	0.43	−0.21	0.45
	Classical	0.18	0.58	−0.21	−0.46
gene programs defined by scRNA-seq	Cell Cycle	0.22	−0.03	0.42	−0.69
	partial EMT	0.67	−0.68	0.12	0.04
	Epithelial_differentiation_1	−0.41	−0.54	−0.32	0.8
	Epithelial_differentiation_2	−0.25	−0.59	0.48	0.44
	Stress	−0.1	−0.49	−0.11	0.51
	Hypoxia	0.23	−0.38	−0.19	0.2
Program-1 tissue remodeling, partial EMT	GOBP_HEMIDESMOSOME_ASSEMBLY	0.89	−0.62	−0.27	−0.07
	GOBP_REGULATION_OF_VITAMIN_D_BIOSYNTHETIC_PROCESS	0.82	−0.75	0.06	−0.14
	GOBP_ENTRY_OF_BACTERIUM_INTO_HOST_CELL	0.66	−0.2	−0.6	0.08
	GOBP_WOUND_HEALING_SPREADING_OF_EPIDERMAL_CELLS	0.61	−0.42	−0.63	0.32
	GOBP_POSITIVE_REGULATION_OF_EXTRINSIC_APOPTOTIC_SIGNALING_PATHWAY_VIA_DEATH_DOMAIN_REC	0.59	−0.64	−0.15	0.22
	GOBP_BASEMENT_MEMBRANE_ORGANIZATION	0.53	−0.42	−0.19	−0.01
	GOBP_CELL_SUBSTRATE_JUNCTION_ORGANIZATION	0.52	−0.36	−0.43	0.31
	GOBP_CELL_ADHESION_MEDIATED_BY_INTEGRIN	0.5	−0.31	−0.27	0
	HALLMARK_MITOTIC_SPINDLE	0.5	−0.1	−0.43	−0.12
	HALLMARK_EPITHELIAL_MESENCHYMAL_TRANSITION	0.48	−0.43	0.03	0.14
	GOBP_FOCAL_ADHESION_ASSEMBLY	0.48	−0.35	−0.44	0.34
	HALLMARK_PROTEIN_SECRETION	0.48	−0.28	−0.41	0.05
	GOBP_EPIBOLY	0.47	−0.36	−0.43	0.13
	HALLMARK_ANGIOGENESIS	0.44	−0.4	−0.03	0.1
	HALLMARK_G2M_CHECKPOINT	0.4	0.14	−0.24	−0.49
	HALLMARK_UV_RESPONSE_DN	0.4	−0.11	−0.44	0.31
	HALLMARK_TGF_BETA_SIGNALING	0.39	−0.44	−0.32	0.24
	HALLMARK_E2F_TARGETS	0.37	0.15	0.21	−0.62
	HALLMARK_IL6_JAK_STAT3_SIGNALING	0.35	−0.52	0.04	0.23
	HALLMARK_APICAL_JUNCTION	0.31	−0.37	−0.18	0.15
	HALLMARK_TNFA_SIGNALING_VIA_NFKB	0.28	−0.5	−0.03	0.27
	HALLMARK_MYC_TARGETS_V1	0.27	−0.23	0.27	−0.53
Program-2 metabolic process, Classical-like	GOBP_FLAVONOID_METABOLIC_PROCESS	−0.36	0.75	−0.02	0.14
	GOBP_SKELETAL_MUSCLE_SATELLITE_CELL_DIFFERENTIATION	−0.05	0.71	−0.37	0.09
	GOBP_ETHANOL_OXIDATION	−0.62	0.7	0.01	0.05
	GOBP_CELLULAR_GLUCURONIDATION	−0.26	0.69	0	−0.02
	GOBP_LATERAL_SPROUTING_FROM_AN_EPITHELIUM	0.07	0.68	−0.34	0.2
	GOBP_URONIC_ACID_METABOLIC_PROCESS	−0.39	0.61	0.05	0.09
	GOBP_DETECTION_OF_CHEMICAL_STIMULUS_INVOLVED_IN_SENSORY_PERCEPTION_OF_TASTE	−0.53	0.51	0.18	0.04
	GOBP_CILIUM_MOVEMENT	−0.33	0.36	−0.04	−0.1
	GOBP_HISTONE_H4_ACETYLATION	−0.03	0.3	−0.08	−0.33
	HALLMARK_SPERMATOGENESIS	−0.14	0.28	−0.06	−0.24
	GOBP_INTERSTRAND_CROSS_LINK_REPAIR	0.18	0.22	−0.03	−0.54
Program-3 respiration, Myc activation, cell cycle, interferon signaling	GOBP_MITOCHONDRIAL_ELECTRON_TRANSPORT_NADH_TO_UBIQUINONE	−0.59	−0.44	0.68	0.14
	GOBP_NADH_DEHYDROGENASE_COMPLEX_ASSEMBLY	−0.61	−0.32	0.65	−0.14
	GOBP_ATP_SYNTHESIS_COUPLED_ELECTRON_TRANSPORT	−0.55	−0.28	0.65	−0.06
	GOBP_MITOCHONDRIAL_RESPIRATORY_CHAIN_COMPLEX_ASSEMBLY	−0.55	−0.27	0.63	−0.27
	GOBP_RESPIRATORY_ELECTRON_TRANSPORT_CHAIN	−0.51	−0.28	0.6	−0.17
	GOBP_OXIDATIVE_PHOSPHORYLATION	−0.49	−0.32	0.6	−0.07
	GOBP_CYTOCHROME_COMPLEX_ASSEMBLY	−0.44	−0.12	0.56	−0.53
	GOBP_ELECTRON_TRANSPORT_CHAIN	−0.45	−0.3	0.54	−0.06
	GOBP_REGULATION_OF_CELLULAR_AMINO_ACID_METABOLIC_PROCESS	0.08	−0.31	0.5	−0.29
	HALLMARK_INTERFERON_ALPHA_RESPONSE	0.24	−0.71	0.49	0.35
	HALLMARK_OXIDATIVE_PHOSPHORYLATION	−0.43	−0.16	0.46	−0.11
	GOBP_CELLULAR_RESPIRATION	−0.42	−0.19	0.44	−0.1
	HALLMARK_MYC_TARGETS_V2	0.27	−0.21	0.44	−0.62
	HALLMARK_DNA_REPAIR	−0.03	−0.24	0.41	−0.41
	HALLMARK_REACTIVE_OXYGEN_SPECIES_PATHWAY	−0.4	−0.06	0.23	0.17
	HALLMARK_FATTY_ACID_METABOLISM	−0.29	0.07	0.12	0.12
Program-4 immune/stress-response, epithelial differentiation	GOBP_PEPTIDE_CROSS_LINKING	0.01	−0.53	−0.27	0.77
	GOBP_CORNIFICATION	−0.2	−0.51	−0.29	0.76
	GOBP_KERATINIZATION	−0.25	−0.48	−0.24	0.73
	GOBP_REGULATION_OF_WATER_LOSS_VIA_SKIN	0.03	−0.44	−0.4	0.64
	GOBP_KERATINOCYTE_DIFFERENTIATION	0.08	−0.42	−0.35	0.62
	GOBP_EPIDERMAL_CELL_DIFFERENTIATION	0.03	−0.32	−0.31	0.55
	GOBP_SKIN_DEVELOPMENT	0.24	−0.27	−0.35	0.51
	GOBP_EPIDERMIS_DEVELOPMENT	0.18	−0.25	−0.31	0.48
	GOBP_POSITIVE_REGULATION_OF_MACROAUTOPHAGY	0.29	−0.34	−0.47	0.46
	GOBP_WATER_HOMEOSTASIS	0.02	−0.11	−0.27	0.44
	HALLMARK_INFLAMMATORY_RESPONSE	0.35	−0.55	−0.02	0.41
	HALLMARK_INTERFERON_GAMMA_RESPONSE	0.26	−0.58	0.31	0.35
	HALLMARK_ALLOGRAFT_REJECTION	0.11	−0.49	0.25	0.29
	HALLMARK_COMPLEMENT	0.24	−0.39	0.06	0.28
	HALLMARK_KRAS_SIGNALING_UP	0.23	−0.26	−0.11	0.26
	HALLMARK_ADIPOGENESIS	−0.24	−0.05	0.07	0.11

gene set enrichement score (color scale: −0.5, 0, 0.5)

HNSCC

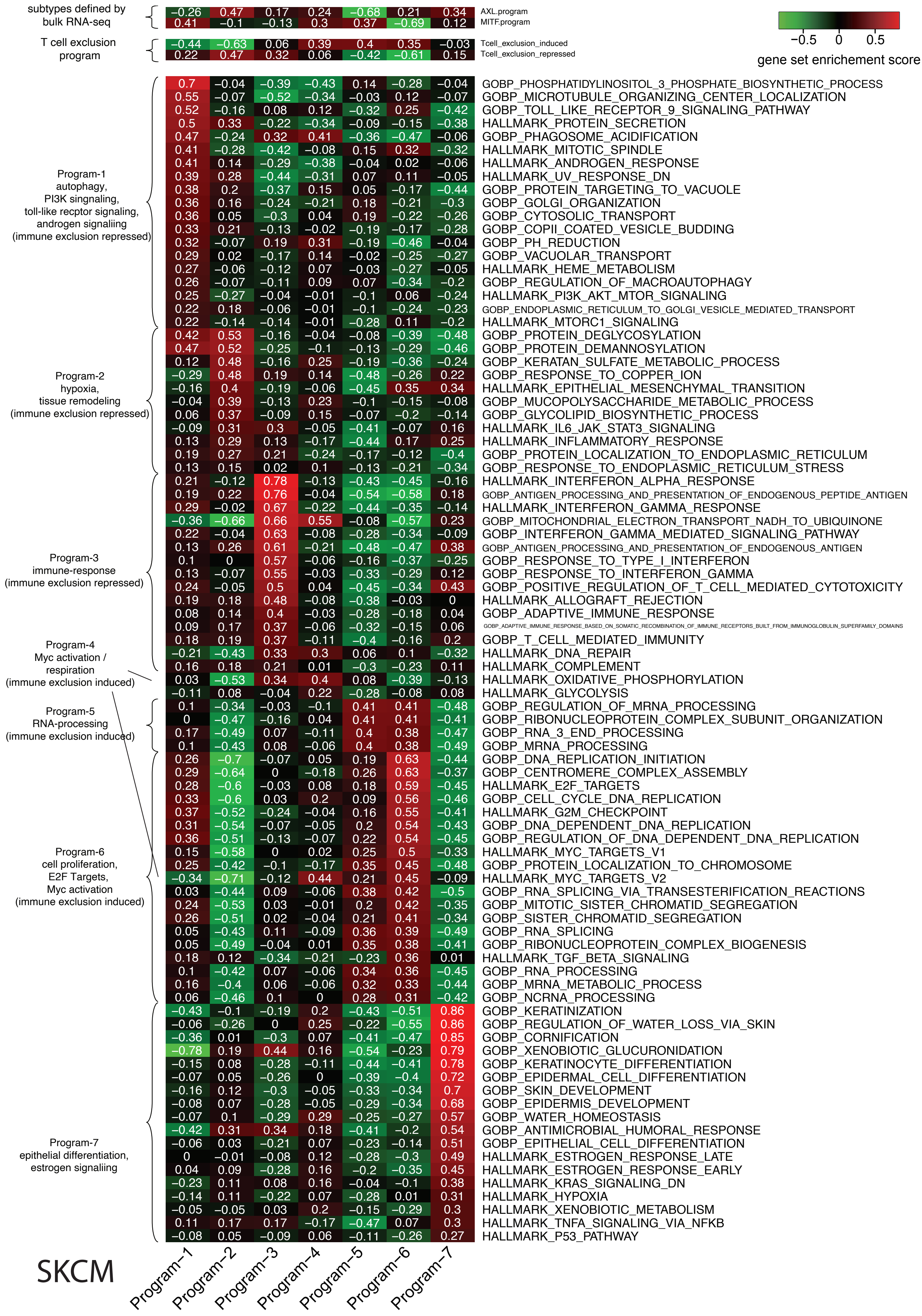

Fig. SN11 | Heatmap shows the gene set enrichment score by GSVA for subtype marker genes and MgSigDB biological process for each gene program of TCGA-GBM (a), TCGA-HNSCC (b), and TCGA-SKCM (c) inferred by BayesPrism.

a

TCGA-GBM

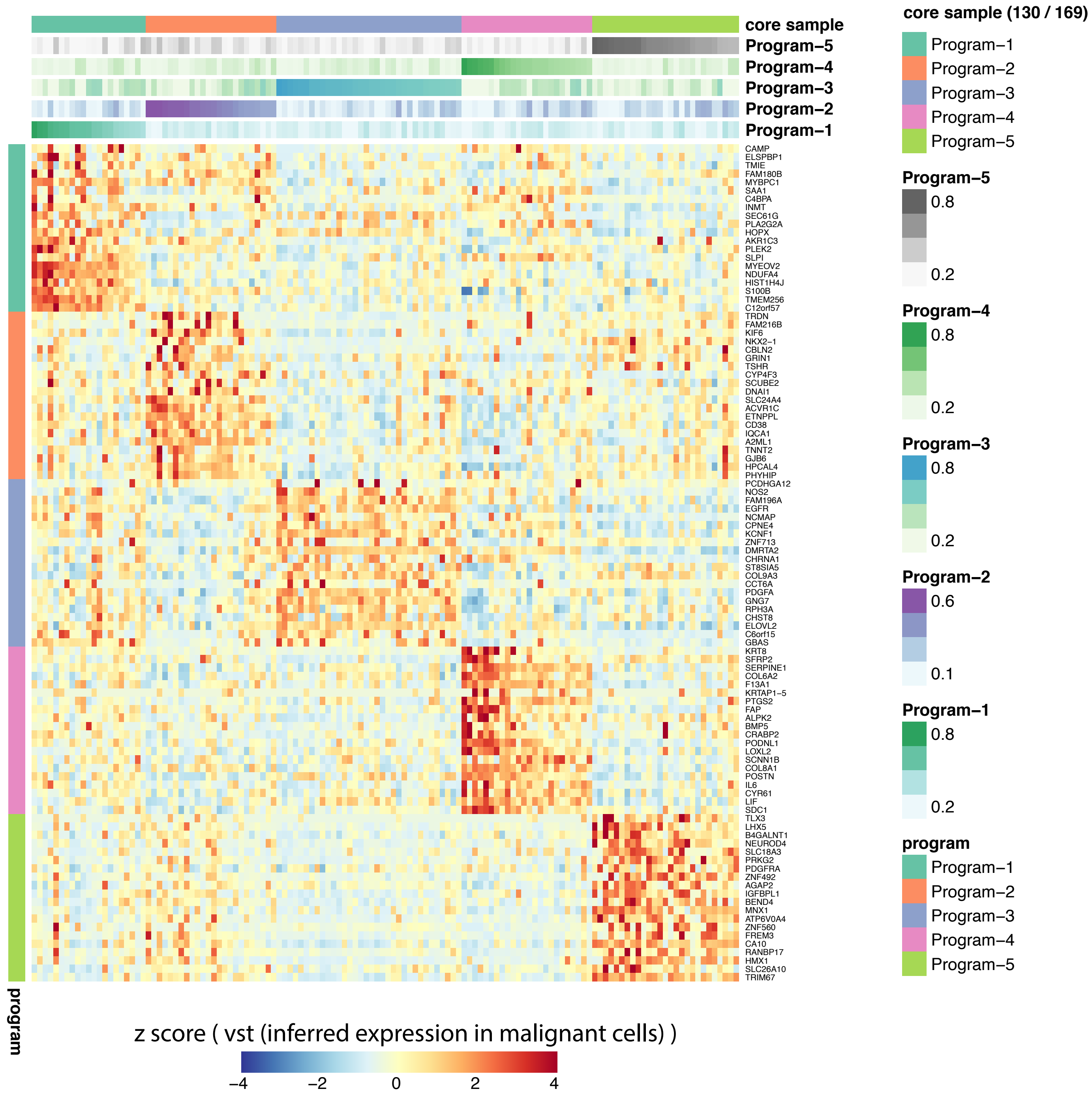
core sample
Program−5
Program−4
Program−3
Program−2
Program−1
CAMP
ELSPBP1
TMIE
FAM180B
MYBPC1
SAA1
C4BPA
INMT
SEC61G
PLA2G2A
HOPX
AKR1C3
PLEK2
SLPI
MYEOV2
NDUFA4
HIST1H4J
S100B
TMEM256
C12orf57
TRDN
FAM216B
KIF6
NKX2−1
CBLN2
GRIN1
TSHR
CYP4F3
SCUBE2
DNAI1
SLC24A4
ACVR1C
ETNPPL
CD38
IQCA1
A2ML1
TNNT2
GJB6
HPCAL4
PHYHIP
PCDHGA12
NOS2
FAM196A
EGFR
NCMAP
CPNE4
KCNF1
ZNF713
DMRTA2
CHRNA1
ST8SIA5
COL9A3
CCT6A
PDGFA
GNG7
RPH3A
CHST8
ELOVL2
C6orf15
GBAS
KRT8
SFRP2
SERPINE1
COL6A2
F13A1
KRTAP1−5
PTGS2
FAP
ALPK2
BMP5
CRABP2
PODNL1
LOXL2
SCNN1B
COL8A1
POSTN
IL6
CYR61
LIF
SDC1
TLX3
LHX5
B4GALNT1
NEUROD4
SLC18A3
PRKG2
PDGFRA
ZNF492
AGAP2
IGFBPL1
BEND4
MNX1
ATP6V0A4
ZNF560
FREM3
CA10
RANBP17
HMX1
SLC26A10
TRIM67
program
z score (vst (inferred expression in malignant cells))
−4
−2
0
2
4
core sample (130 / 169)
Program−1
Program−2
Program−3
Program−4
Program−5
Program−5
0.8
0.2
Program−4
0.8
0.2
Program−3
0.8
0.2
Program−2
0.6
0.1
Program−1
0.8
0.2
program
Program−1
Program−2
Program−3
Program−4
Program−5

b

TCGA-HNSCC

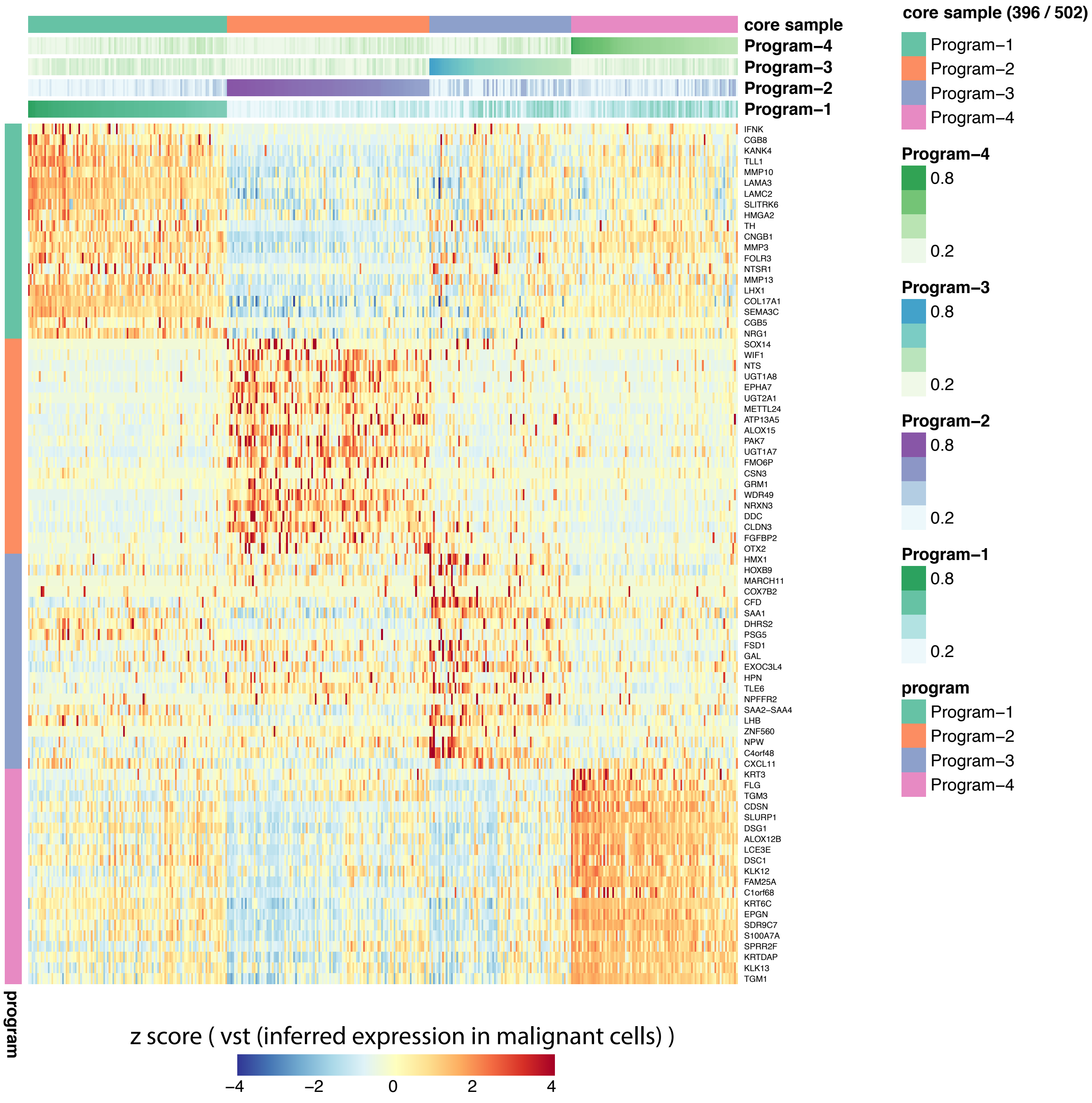
core sample
Program–4
Program–3
Program–2
Program–1
IFNK
CGB8
KANK4
TLL1
MMP10
LAMA3
LAMC2
SLITRK6
HMGA2
TH
CNGB1
MMP3
FOLR3
NTSR1
MMP13
LHX1
COL17A1
SEMA3C
CGB5
NRG1
SOX14
WIF1
NTS
UGT1A8
EPHA7
UGT2A1
METTL24
ATP13A5
ALOX15
PAK7
UGT1A7
FMO6P
CSN3
GRM1
WDR49
NRXN3
DDC
CLDN3
FGFBP2
OTX2
HMX1
HOXB9
MARCH11
COX7B2
CFD
SAA1
DHRS2
PSG5
FSD1
GAL
EXOC3L4
HPN
TLE6
NPFFR2
SAA2–SAA4
LHB
ZNF560
NPW
C4orf48
CXCL11
KRT3
FLG
TGM3
CDSN
SLURP1
DSG1
ALOX12B
LCE3E
DSC1
KLK12
FAM25A
C1orf68
KRT6C
EPGN
SDR9C7
S100A7A
SPRR2F
KRTDAP
KLK13
TGM1
program
z score (vst (inferred expression in malignant cells))
–4
–2
0
2
4
core sample (396 / 502)
Program–1
Program–2
Program–3
Program–4
Program–4
0.8
0.2
Program–3
0.8
0.2
Program–2
0.8
0.2
Program–1
0.8
0.2
program
Program–1
Program–2
Program–3
Program–4

c

TCGA-SKCM

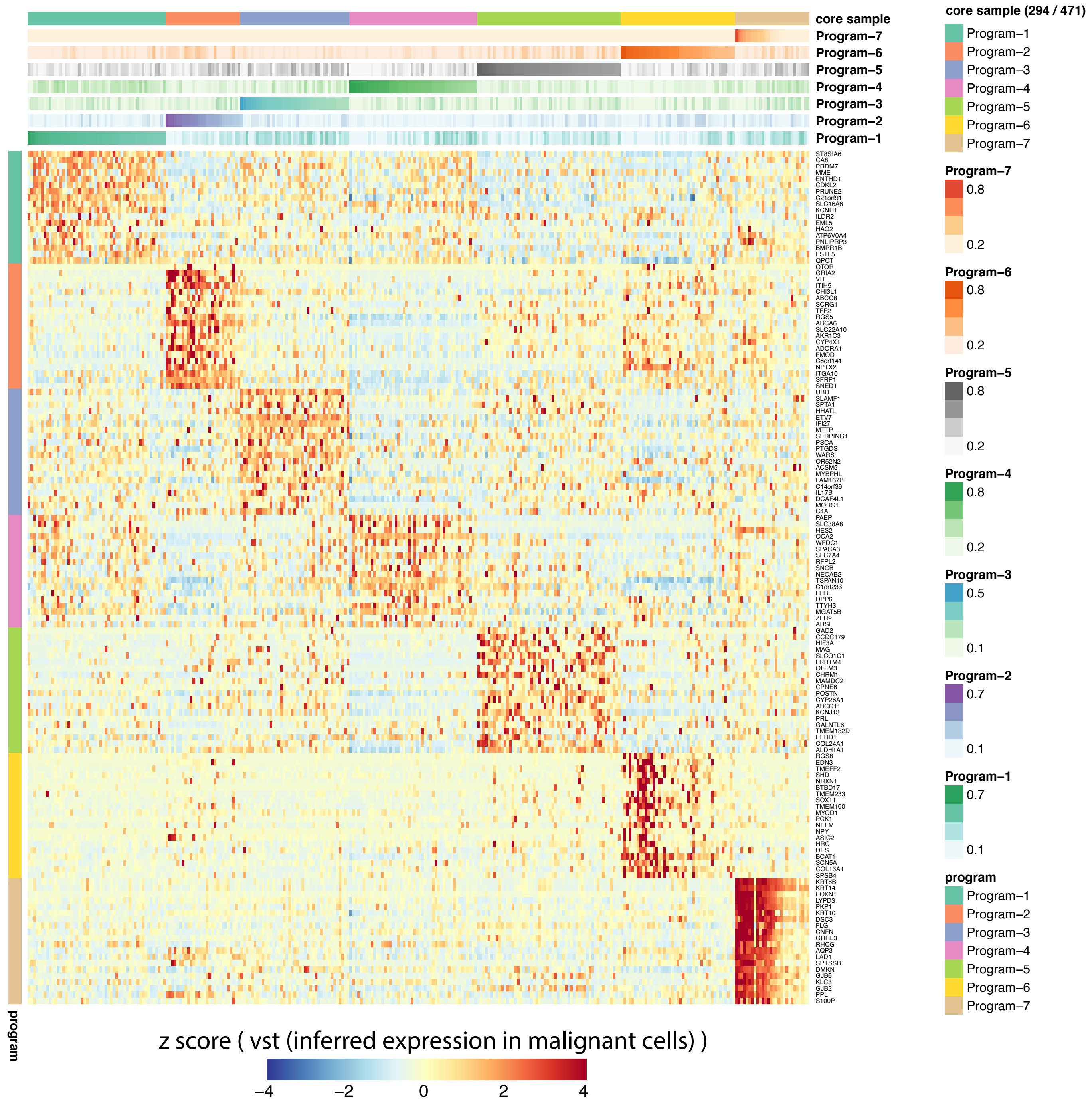

Fig. SN12 | Heatmap shows the relative expression of top 20 differentially expressed genes of each gene program across a set of TCGA bulk samples representing each gene program. Each row represents a gene, while each column represents a TCGA bulk sample. The expression level was colored by the z score of variance-stabilizing transformed expression in malignant cells deconvolved by BayesPrism. Bulk samples were grouped by their affiliation to their gene program and ordered by normalized program weights (top annotation heatmaps). Genes were selected and ordered from high to low by the $\log_2$ fold change compared to samples affiliated to other gene programs.

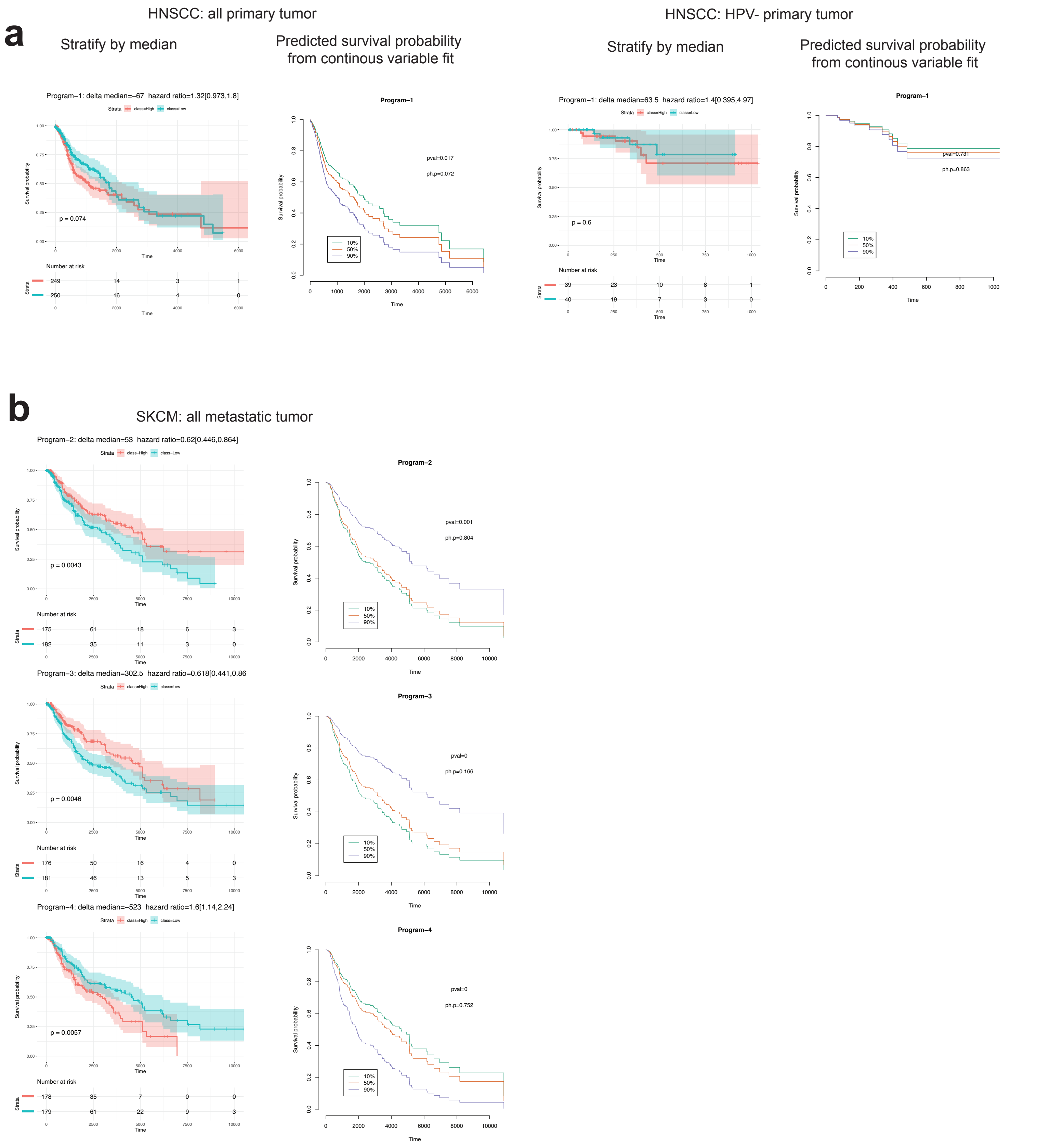

Fig. SN13 | KM plots of gene programs in malignant cells with significant association with survival by at least one model. Two methods of regression were shown: stratifying patients using the median cutoff, or treating the normalized program weights as continuous variables in the Cox proportional-hazards model. For median-cutoff models, p values were derived from the log-rank test. Hazard ratio was defined by high / low, and the 95 percentile confidence interval was shown in the square brackets. Transparent colors mark 95% confidence bands. The log-rank test was used for the median-cutoff model. The survival curves in the right column were plotted by conditioning on the program weights at its 10%, 50% and 90% percentile, and then predicting survival probabilities based on the continuous variable Cox regression model, which was only used for the purpose of visualization. Two p values were computed for the continuous variable Cox regression model: “pval” was generated by the Wald test indicating the statistical significance of survival association, while “ph.p” was generated by the chi-squared test for scaled Schoenfeld residuals to check the proportional hazards assumption.

We also observed concordance between the tumor-microenvironment correlation identified using gene programs and using individual genes. Two approaches have been used to summarize the correlation between the gene expression in malignant cells and cell type fractions of non-malignant cells. In the first approach, we computed the genewise correlation between its inferred expression in malignant cells and the fraction of a non-malignant cell type, and then performed gene set enrichment analysis over the rank order of the correlation coefficient for each non-malignant cell type (**Fig. 3d**).

In the second approach, we used the embedding learning module of BayesPrism to factorize the expression of malignant cells into the linear combination of multiple malignant bases. Each tumor sample was assigned with a group of weights over these bases. We then perform differential gene expression over a set of representative core samples for each tumor basis (**Supplementary Table 5**, **Fig. SN12**), followed by gene set enrichment analysis over the Wald statistics of the differential expression (**Fig. 4e-g**, **Supplementary Table 4**). Two approaches tackle the problem from different angles. The genewise approach studies the correlation with a particular non-malignant cell type of interest, while the gene program level approach focuses on the selection of a group of co-expressed genes, regardless of the correlation with non-malignant cell types. Although the embedding learning approach does not directly look for correlations with the fraction of non-malignant cells, we found that it yielded many observations that were concordant with the results obtained from the genewise approach, which are summarized below.

	Direction of correlation with cell type fractions	Consistently enriched Biological processes
GBM		
program-1	endothelial(-)	respiration / oxidative phosphorylation pathways
program-2	oligodendrocyte(+)	neuronal processes
program-3	oligodendrocyte(-)	DNA conformational change and RNA processing
program-4	macrophage(+); endothelial(+); pericyte(+)	hypoxia, immune response, EMT and remodeling of extracellular matrix
program-5	macrophage(-)	Proliferation, DNA replication and cell cycle
HNSCC		
program-1	B cell(-); T cell(-)	EMT
program-2	endothelial(+); T cell(+)	Hallmark E2F targets (DNA replication)
program-3	fibroblast(-); macrophage(+); mast cell(-)	Respiration [fibroblast(-)]; Interferon [fibroblast(-) & macrophage(+)]; DNA repair [mast cell(-)]

program-4	endothelial(-); fibroblast(-); T cell(-); dendritic cell(-); macrophage(-); mast cell(+)	keratinization
SKCM		
program-2	fibroblast(+); endothelial(+)	EMT, NFkB, hypoxia, cell adhesion, extracellular matrix organization, vesicle transport/budding, and ER stress
program-3	CD4+ T cell(+); CD8+ T cell(+); NK cell(+); macrophage(+); B cell(+)	Immune response
program-5	macrophage(-); fibroblast(-)	RNA processing [macrophage(-)]; Respiration [fibroblast(-)]
program-6	endothelial(+)	EMT

Supplementary Note 11: Spatial heterogeneity in cell type composition in GBM

To understand the spatial distribution of cells within a tumor, we applied BayesPrism to 122 bulk RNA-seq samples from the Ivy Glioblastoma Atlas Project (IVY GAP) that interrogate laser microdissected tissue from GBM[20]. Data was available for ten tumors microdissected into five structures: leading edge (LE), infiltrating tumor (IT), cellular tumor (CT), microvascular proliferation (MVP) and pseudopalisading cells around necrosis (PAN) (**Fig. 5a and Supplementary Table 3b**).

As we expected normal brain cells, including neurons, at high abundance in the leading edge and infiltrating tumor structures[20], we deconvolved all samples using a reference scRNA-seq dataset by combining refGBM8 with adult human neurons collected using Fluidigm[21]. As batch effects exist between refGBM8 and Fluidigm, the absolute fraction of cell types can be affected. In particular, as the neuron cell reference collected by Fluidigm was sequenced to a much higher total sequencing depth and detected more genes than refGBM8, it may absorb more reads than other cell types in refGBM8, and hence its absolute fraction is likely to be overestimated. This is similar to what we observe for T cells in the pseudo-bulk deconvolution of GBM28 using refGBM8 (see the scatter plot associated with the **Source Data Fig.1**). We observed that due to the significantly lower number of total reads caused by the scarcity of T cells in GBM, the inferred absolute fraction was off by a linear factor, while the relative fraction across samples remained accurate. BySN the same token, the relative cell type fractions of the deconvolved IVY-GAP data shall also remain accurate, and hence the results from statistical tests remain valid.

BayesPrism revealed several striking features of GBM regional cellular heterogeneity (**Fig. 5b**). First, pericytes and endothelial cells were significantly enriched in regions of microvascular proliferation (p < 1e-4, linear mixed model, see Methods), and comprised nearly 60% of cells in these regions. Second, oligodendrocytes and adult neurons were enriched in the leading edge and infiltrating tumor (p < 1e-4, linear mixed model), with a relative magnitude that matches H&E stained sections from these same patients[20]. Third, pseudopalisading cells around necrosis showed a depletion of endothelial cells (p = 0.0499, linear mixed model) and enrichment for T cells (p = 0.0048, linear mixed model) and macrophages (p = 0.0140, linear mixed model). Fourth, leading edges were enriched for macrophages (p = 0.0138, linear mixed model) and depleted of pericytes (p = 0.0423, linear mixed model). Taken together, BayesPrism revealed a rich and highly heterogeneous picture of cell type composition.

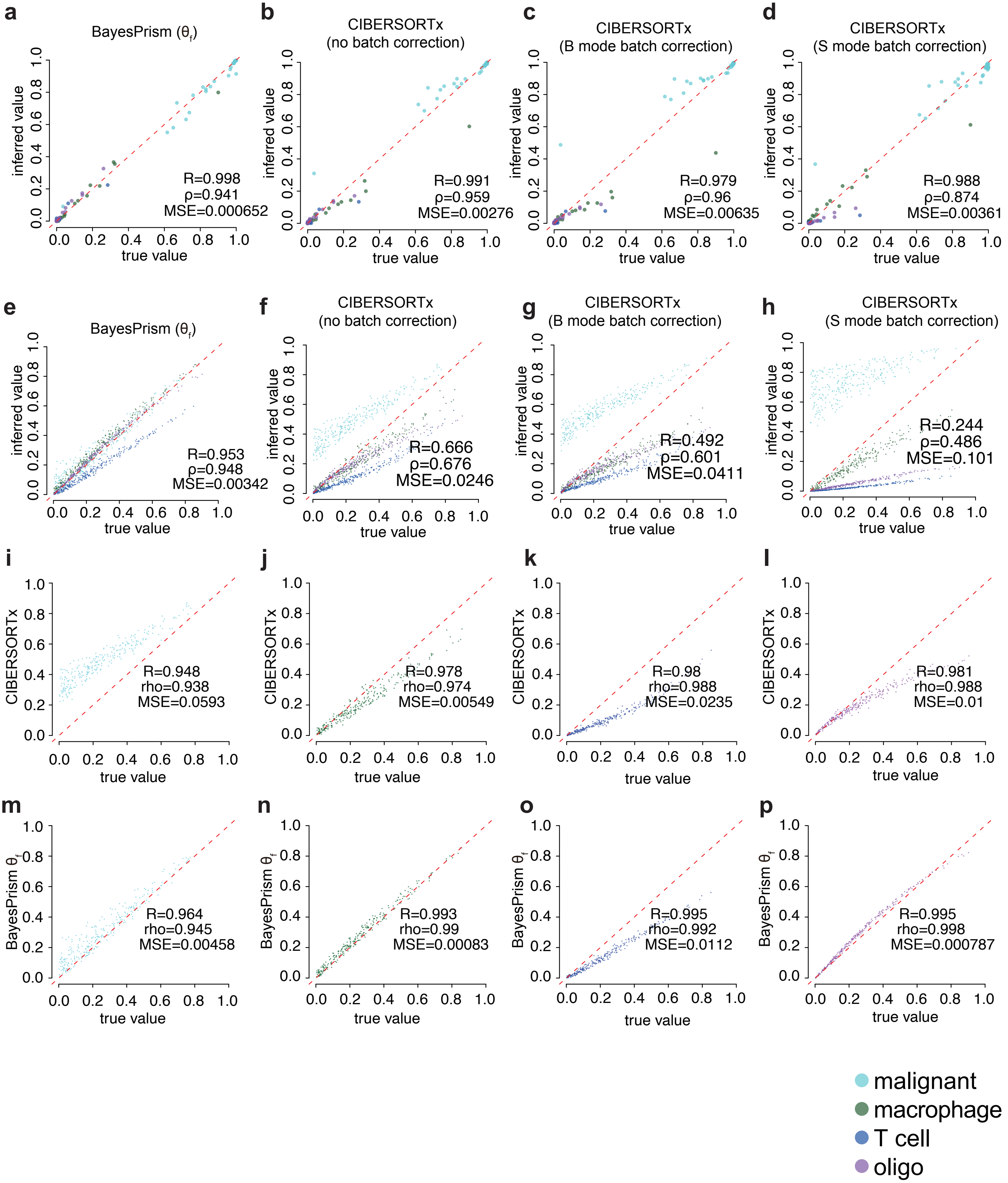

Supplementary Fig. 1 | Comparison between BayesPrism and various modes of CIBERSORTx. Scatter plots show the inferred cell type fraction in the pseudo-bulk GBM28 (**a**-**d**), and a pseudo-bulk dataset containing 270 simulated samples (**e**-**h**). (**i**-**p**) Scatter plots show the performance of individual cell types in **e** and **f**.

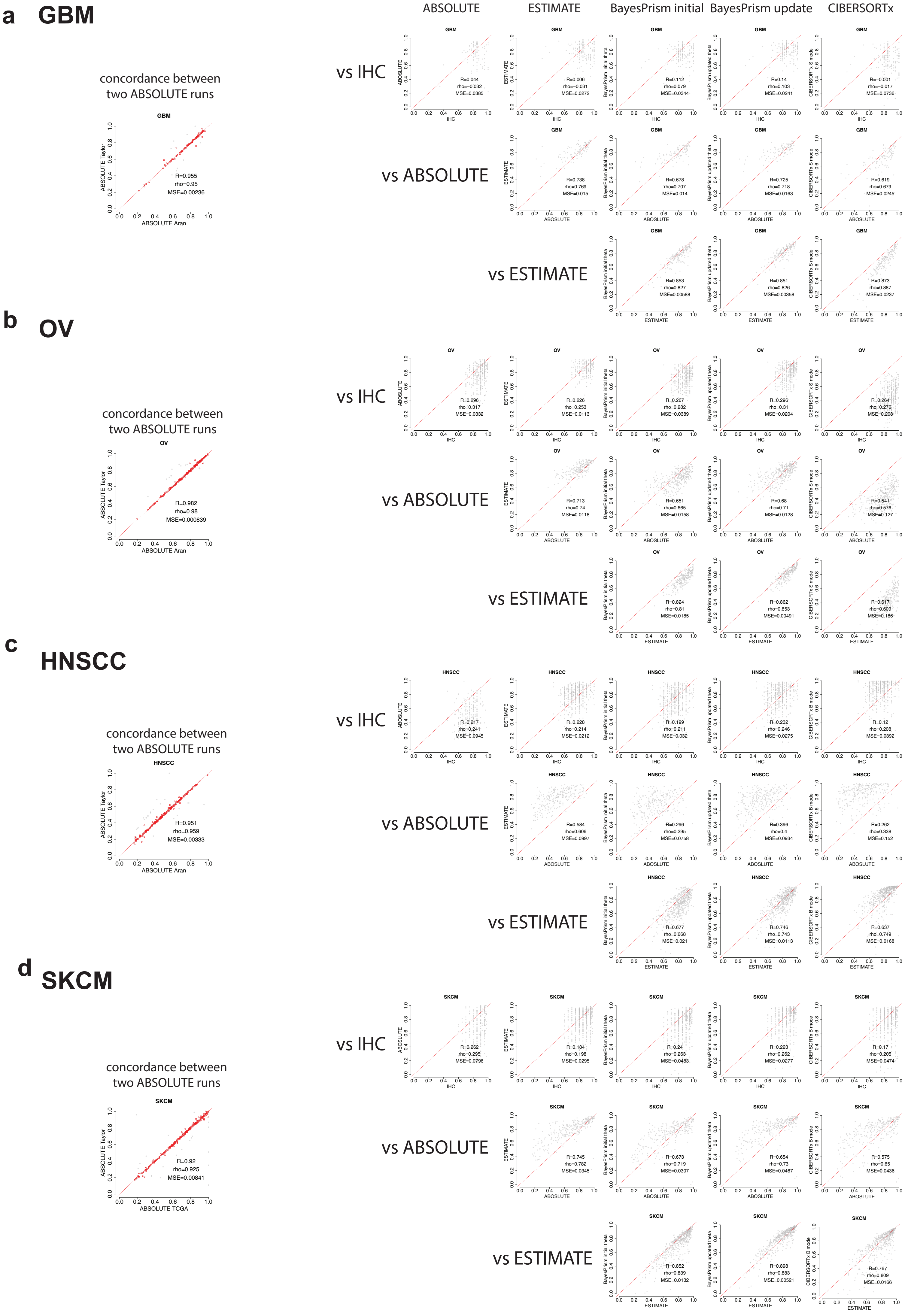

a GBM
ABSOLUTE
ESTIMATE
BayesPrism initial
BayesPrism update
CIBERSORTx
concordance between two ABSOLUTE runs
GBM
ABSOLUTE Taylor
ABSOLUTE Aran
R=0.955
rho=0.95
MSE=0.00236
vs IHC
R=0.044
rho=-0.032
MSE=0.0385
R=0.006
rho=-0.031
MSE=0.0272
R=0.112
rho=0.079
MSE=0.0344
R=0.14
rho=0.103
MSE=0.0241
R=-0.001
rho=-0.017
MSE=0.0736
vs ABSOLUTE
R=0.738
rho=0.769
MSE=0.015
R=0.678
rho=0.707
MSE=0.014
R=0.725
rho=0.718
MSE=0.0163
R=0.619
rho=0.679
MSE=0.0245
vs ESTIMATE
R=0.853
rho=0.827
MSE=0.00588
R=0.851
rho=0.826
MSE=0.00358
R=0.873
rho=0.887
MSE=0.0237
b OV
concordance between two ABSOLUTE runs
OV
ABSOLUTE Taylor
ABSOLUTE Aran
R=0.982
rho=0.98
MSE=0.000839
vs IHC
R=0.296
rho=0.317
MSE=0.0332
R=0.226
rho=0.253
MSE=0.0113
R=0.267
rho=0.282
MSE=0.0389
R=0.296
rho=0.31
MSE=0.0204
R=0.264
rho=0.276
MSE=0.208
vs ABSOLUTE
R=0.713
rho=0.74
MSE=0.0118
R=0.651
rho=0.665
MSE=0.0158
R=0.68
rho=0.71
MSE=0.0128
R=0.541
rho=0.576
MSE=0.127
vs ESTIMATE
R=0.824
rho=0.81
MSE=0.0185
R=0.862
rho=0.853
MSE=0.00491
R=0.617
rho=0.609
MSE=0.186
c HNSCC
concordance between two ABSOLUTE runs
HNSCC
ABSOLUTE Taylor
ABSOLUTE Aran
R=0.951
rho=0.959
MSE=0.00333
vs IHC
R=0.217
rho=0.241
MSE=0.0945
R=0.228
rho=0.214
MSE=0.0212
R=0.199
rho=0.211
MSE=0.032
R=0.232
rho=0.246
MSE=0.0275
R=0.12
rho=0.208
MSE=0.0392
vs ABSOLUTE
R=0.584
rho=0.606
MSE=0.0997
R=0.296
rho=0.295
MSE=0.0758
R=0.396
rho=0.4
MSE=0.0934
R=0.262
rho=0.338
MSE=0.152
vs ESTIMATE
R=0.677
rho=0.668
MSE=0.021
R=0.746
rho=0.743
MSE=0.0113
R=0.637
rho=0.749
MSE=0.0168
d SKCM
concordance between two ABSOLUTE runs
SKCM
ABSOLUTE Taylor
ABSOLUTE TCGA
R=0.92
rho=0.925
MSE=0.00841
vs IHC
R=0.262
rho=0.295
MSE=0.0796
R=0.184
rho=0.198
MSE=0.0295
R=0.24
rho=0.263
MSE=0.0483
R=0.223
rho=0.262
MSE=0.0277
R=0.17
rho=0.205
MSE=0.0474
vs ABSOLUTE
R=0.745
rho=0.782
MSE=0.0345
R=0.673
rho=0.719
MSE=0.0307
R=0.654
rho=0.73
MSE=0.0467
R=0.575
rho=0.65
MSE=0.0436
vs ESTIMATE
R=0.852
rho=0.839
MSE=0.0132
R=0.898
rho=0.883
MSE=0.00521
R=0.767
rho=0.809
MSE=0.0166

Supplementary Fig. 2 | Comparison between tumor purity inferred by BayesPrism, CIBERSORTx, ABSOLUTE, ESTIMATE and IHC. Scatter plots show the correlation between malignant fractions inferred by each method for TCGA-GBM **(a)**, TCGA-OV **(b)**, TCGA-HNSCC **(c)**, and TCGA-SKCM **(d)**. Dashed lines mark the y=x. As the tumor purity estimated by ABSOLUTE varies between multiple sources (Aran et. al. (2015) Nature Communications; Taylor et. al. (2018) Cancer Cell; TCGAN (2015) Cell) (leftmost columns), we took the mean of ABSOLUTE scores between two sources over a subset of samples whose difference between two sources is smaller than 0.1 (marked by the red dots), and used the averaged score for comparison with other methods (right panels). ESTIMATE and IHC scores were both obtained from Aran et al. CIERSORTx was run using the same input reference matrix as BayesPrism, using the batch correction mode recommended by the authors (S mode for GBM and OV; B mode for SKCM and HNSCC). In HNSCC we observed that ABSOLUTE score was lower than IHC, while tumor purity estimated using transcription signal (ESTIMATE, BayesPrism and CIBERSORTx) were higher than ABSOLUTE, suggesting an underestimation of tumor purity by the DNA copy-number based method, and/or the presence of non-immune non-stromal cells (such as normal epithelial cells, which was not captured by scRNA-seq reference) in the tumor samples.

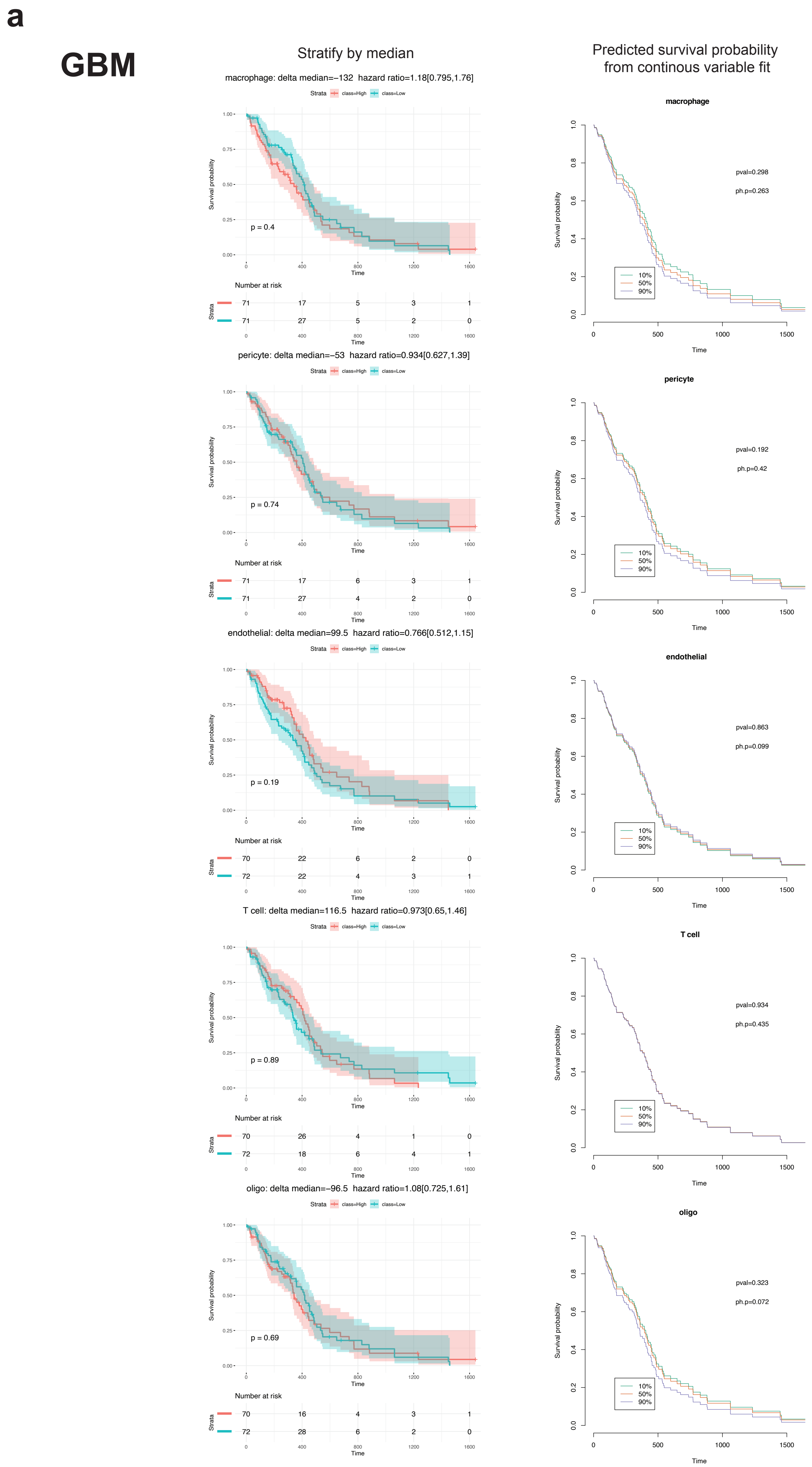

a
GBM
Stratify by median
Predicted survival probability from continous variable fit
macrophage: delta median=−132 hazard ratio=1.18[0.795,1.76]
Strata class=High class=Low
Survival probability
p = 0.4
Time
Number at risk
Strata
71 17 5 3 1
71 27 5 2 0
macrophage
pval=0.298
ph.p=0.263
10%
50%
90%
pericyte: delta median=−53 hazard ratio=0.934[0.627,1.39]
p = 0.74
71 17 6 3 1
71 27 4 2 0
pericyte
pval=0.192
ph.p=0.42
endothelial: delta median=99.5 hazard ratio=0.766[0.512,1.15]
p = 0.19
70 22 6 2 0
72 22 4 3 1
endothelial
pval=0.863
ph.p=0.099
T cell: delta median=116.5 hazard ratio=0.973[0.65,1.46]
p = 0.89
70 26 4 1 0
72 18 6 4 1
T cell
pval=0.934
ph.p=0.435
oligo: delta median=−96.5 hazard ratio=1.08[0.725,1.61]
p = 0.69
70 16 4 3 1
72 28 6 2 0
oligo
pval=0.323
ph.p=0.072

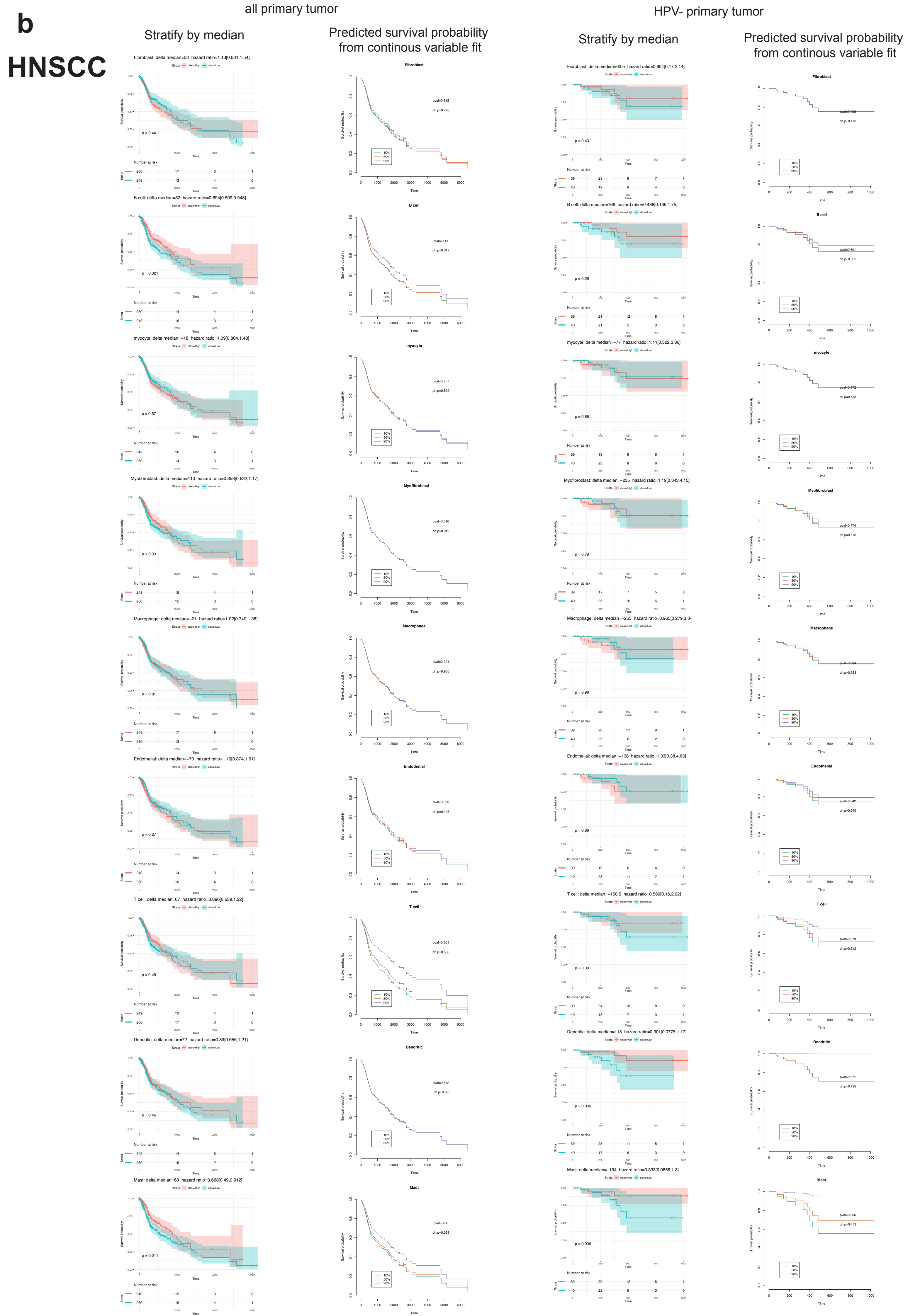
b
HNSCC
all primary tumor
HPV- primary tumor
Stratify by median
Predicted survival probability from continous variable fit
Stratify by median
Predicted survival probability from continous variable fit
Fibroblast: delta median=53 hazard ratio=1.13[0.831,1.54]
B cell: delta median=82 hazard ratio=0.694[0.509,0.948]
myocyte: delta median=-18 hazard ratio=1.09[0.804,1.48]
Myofibroblast: delta median=110 hazard ratio=0.858[0.632,1.17]
Macrophage: delta median=-21 hazard ratio=1.02[0.749,1.38]
Endothelial: delta median=-70 hazard ratio=1.19[0.874,1.61]
T cell: delta median=67 hazard ratio=0.896[0.659,1.22]
Dendritic: delta median=72 hazard ratio=0.89[0.656,1.21]
Mast: delta median=68 hazard ratio=0.668[0.49,0.912]
Fibroblast: delta median=63.5 hazard ratio=0.604[0.17,2.14]
B cell: delta median=166 hazard ratio=0.488[0.136,1.75]
myocyte: delta median=-77 hazard ratio=1.11[0.322,3.86]
Myofibroblast: delta median=-235 hazard ratio=1.19[0.343,4.15]
Macrophage: delta median=-255 hazard ratio=0.965[0.279,3.3
Endothelial: delta median=-138 hazard ratio=1.33[0.38,4.63]
T cell: delta median=-150.5 hazard ratio=0.569[0.16,2.02]
Dendritic: delta median=118 hazard ratio=0.301[0.0775,1.17]
Mast: delta median=-194 hazard ratio=0.333[0.0856,1.3]
Fibroblast
B cell
myocyte
Myofibroblast
Macrophage
Endothelial
T cell
Dendritic
Mast
Survival probability
Time
Number at risk
Strata
10%
50%
90%

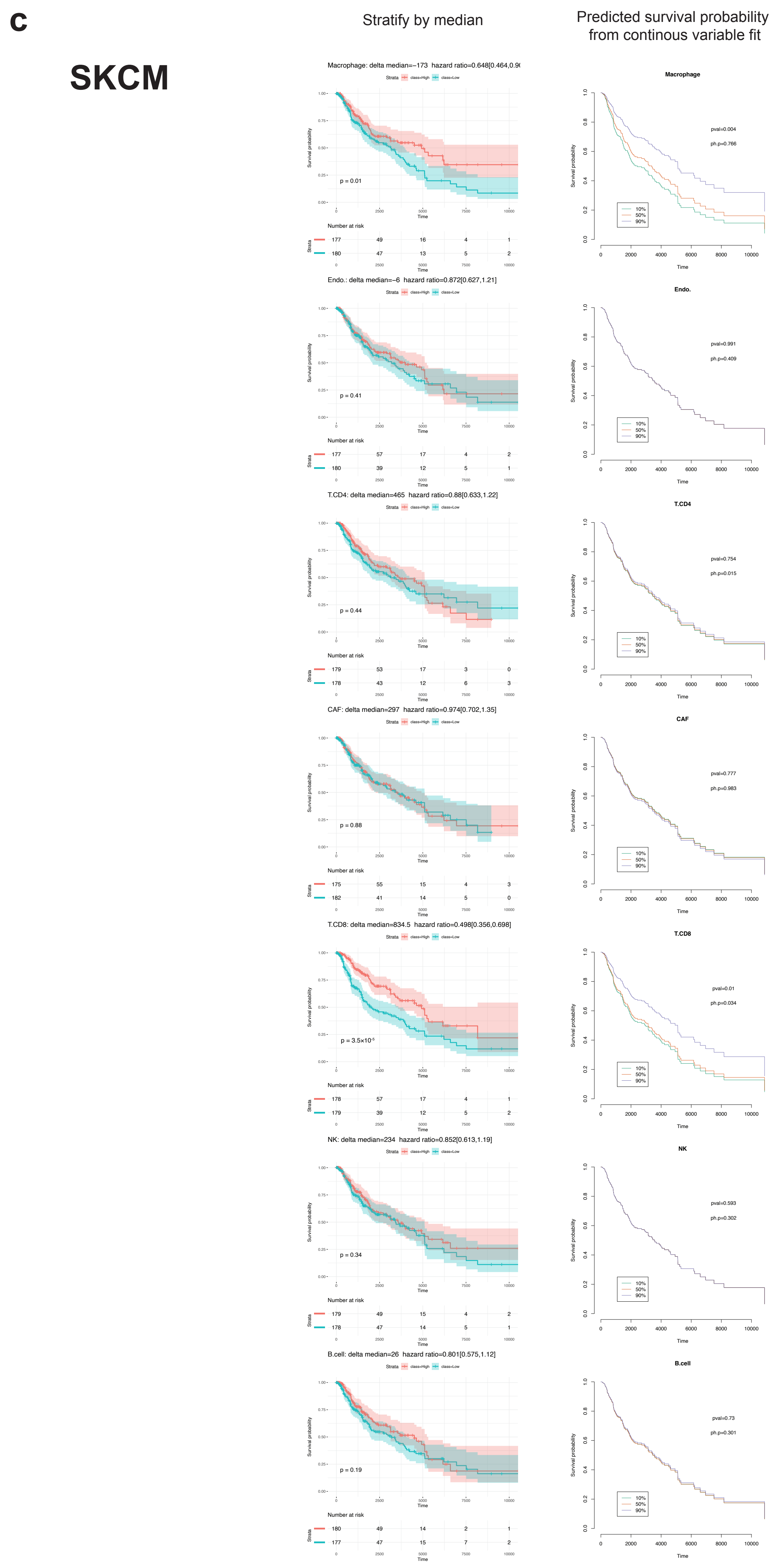
c
SKCM
Stratify by median
Predicted survival probability from continous variable fit
Macrophage: delta median=-173 hazard ratio=0.648[0.464,0.9
p = 0.01
Macrophage
pval=0.004
ph.p=0.766
Endo.: delta median=-6 hazard ratio=0.872[0.627,1.21]
p = 0.41
Endo.
pval=0.991
ph.p=0.409
T.CD4: delta median=465 hazard ratio=0.88[0.633,1.22]
p = 0.44
T.CD4
pval=0.754
ph.p=0.015
CAF: delta median=297 hazard ratio=0.974[0.702,1.35]
p = 0.88
CAF
pval=0.777
ph.p=0.983
T.CD8: delta median=834.5 hazard ratio=0.498[0.356,0.698]
p = 3.5x10⁻⁵
T.CD8
pval=0.01
ph.p=0.034
NK: delta median=234 hazard ratio=0.852[0.613,1.19]
p = 0.34
NK
pval=0.593
ph.p=0.302
B.cell: delta median=26 hazard ratio=0.801[0.575,1.12]
p = 0.19
B.cell
pval=0.73
ph.p=0.301
Number at risk
Survival probability
Time
10%
50%
90%

d

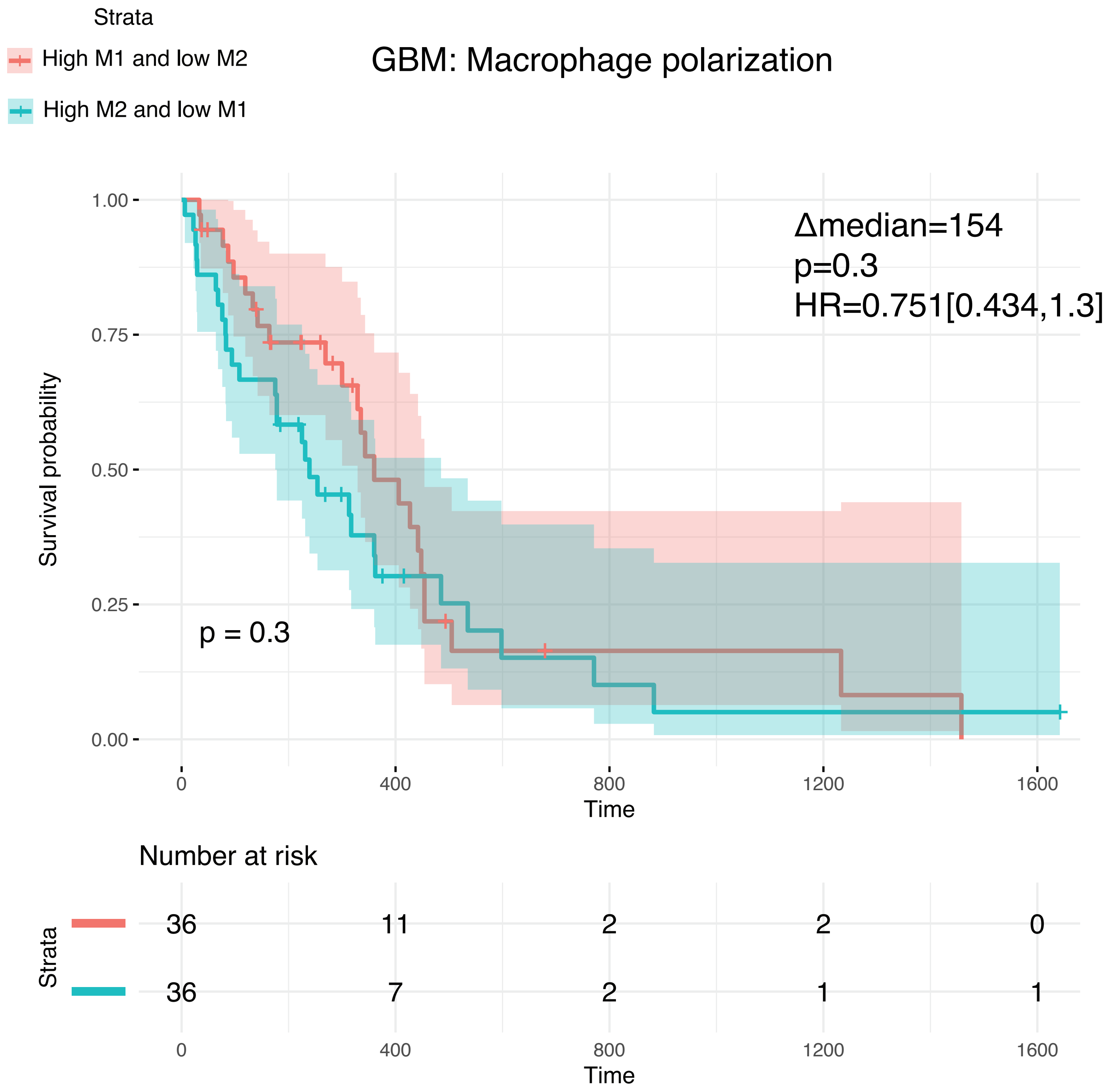

Supplementary Fig. 3 | KM plots of the indicated cell types. (a-c) KM plots inferred from three tumor types across TCGA samples using median cutoff (the left column), and Cox proportional-hazards models in which the cell type fractions were modeled as a continuous variable (the right column). For the KM plots on the left column, p values were derived from the log-rank test. Hazard ratio was defined by high / low, and the 95 percentile confidence interval was shown in the square brackets. Transparent colors mark 95% confidence bands. The survival curves in the right column were plotted by conditioning on the cell type fraction at its 10%, 50% and 90% percentile, and then predicting survival probabilities based on the continuous variable Cox regression model, which was only used for the purpose of visualization. Two p values were computed for the continuous variable Cox regression model: “pval” was generated by the Wald test indicating the statistical significance of survival association, while “ph.p” was generated by the chi-squared test for scaled Schoenfeld residuals to check the proportional hazards assumption. **(d)** KM plot shows the survival association with the M1/M2 state polarization of macrophages in GBM. P values were derived from the log-rank test. Hazard ratio was defined by high / low, and the 95 percentile confidence interval was shown in the square brackets. Transparent colors mark 95% confidence bands.

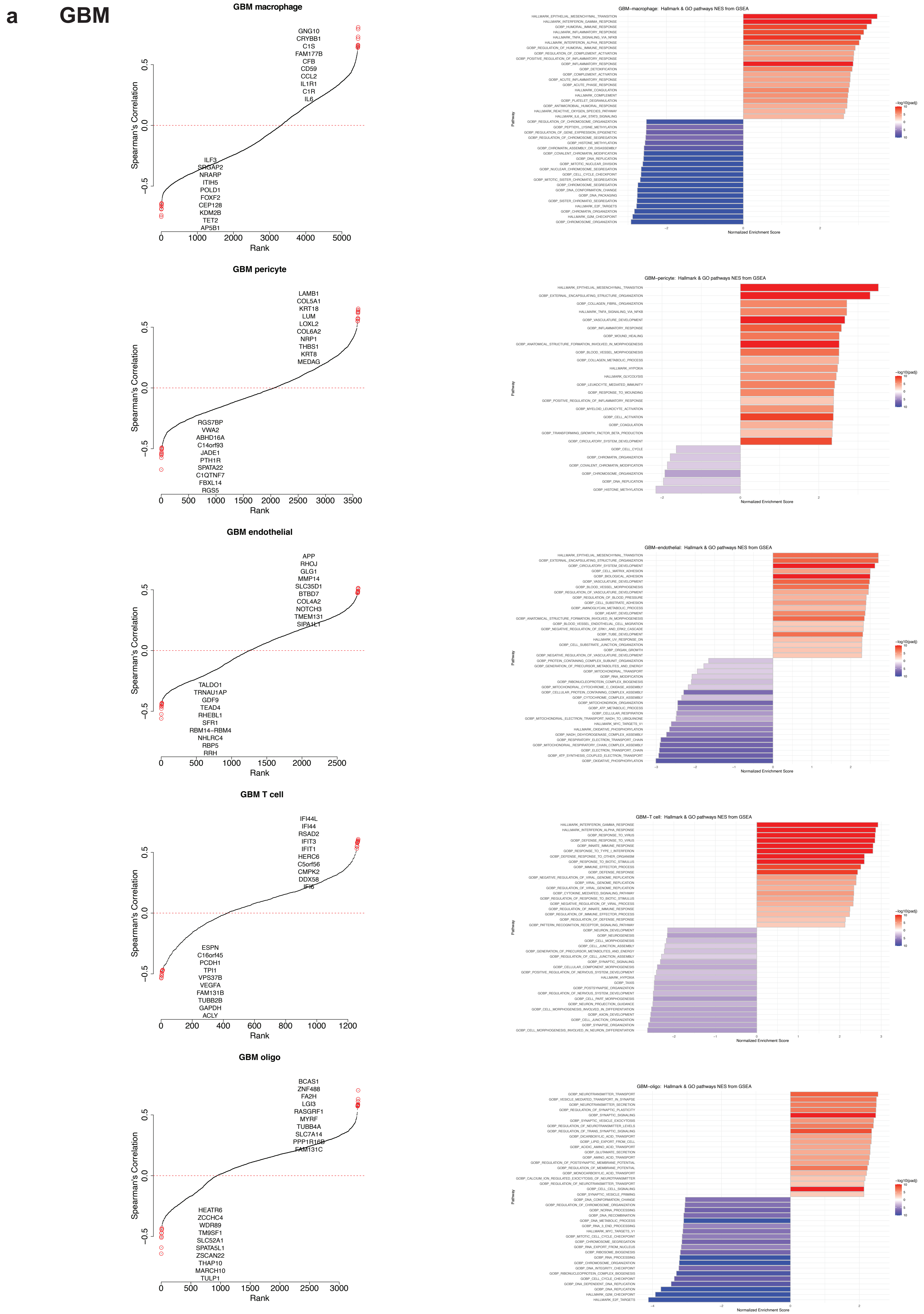

a
GBM
GBM macrophage
GNG10
CRYBB1
C1S
FAM177B
CFB
CD59
CCL2
IL1R1
C1R
IL6
ILF3
SRGAP2
NRARP
ITIH5
POLD1
FOXF2
CEP128
KDM2B
TET2
AP5B1
Spearman's Correlation
Rank
GBM−macrophage: Hallmark & GO pathways NES from GSEA
Pathway
Normalized Enrichment Score
−log10(padj)
GBM pericyte
LAMB1
COL5A1
KRT18
LUM
LOXL2
COL6A2
NRP1
THBS1
KRT8
MEDAG
RGS7BP
VWA2
ABHD16A
C14orf93
JADE1
PTH1R
SPATA22
C1QTNF7
FBXL14
RGS5
GBM−pericyte: Hallmark & GO pathways NES from GSEA
GBM endothelial
APP
RHOJ
GLG1
MMP14
SLC35D1
BTBD7
COL4A2
NOTCH3
TMEM131
SIPA1L1
TALDO1
TRNAU1AP
GDF9
TEAD4
RHEBL1
SFR1
RBM14−RBM4
NHLRC4
RBP5
RRH
GBM−endothelial: Hallmark & GO pathways NES from GSEA
GBM T cell
IFI44L
IFI44
RSAD2
IFIT3
IFIT1
HERC6
C5orf56
CMPK2
DDX58
IFI6
ESPN
C16orf45
PCDH1
TPI1
VPS37B
VEGFA
FAM131B
TUBB2B
GAPDH
ACLY
GBM−T cell: Hallmark & GO pathways NES from GSEA
GBM oligo
BCAS1
ZNF488
FA2H
LGI3
RASGRF1
MYRF
TUBB4A
SLC7A14
PPP1R16B
FAM131C
HEATR6
ZCCHC4
WDR89
TM9SF1
SLC52A1
SPATA5L1
ZSCAN22
THAP10
MARCH10
TULP1
GBM−oligo: Hallmark & GO pathways NES from GSEA

b **HNSCC**

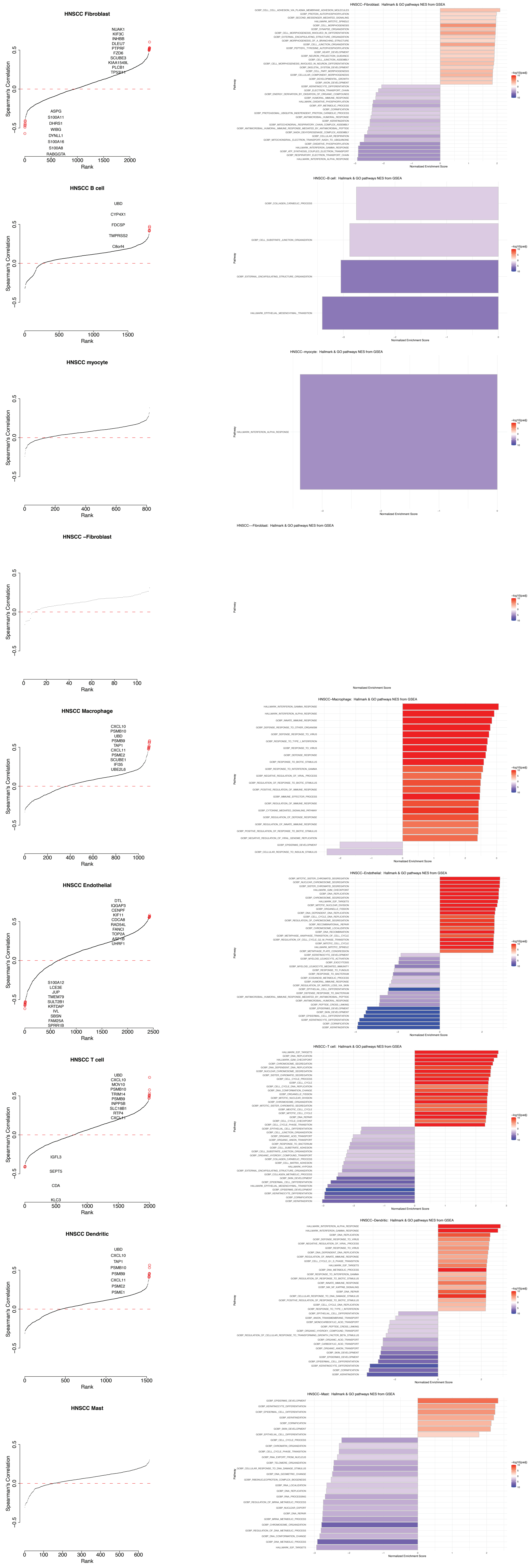
HNSCC Fibroblast
NUAK1
KIF3C
INHBB
DLEU7
PTPRF
FZD6
SCUBE3
KIAA1549L
PLCB1
TP53I11
ASPG
S100A11
DHRS1
WIBG
DYNLL1
S100A16
S100A8
RABGGTA
Spearman's Correlation
Rank
HNSCC−Fibroblast: Hallmark & GO pathways NES from GSEA
Normalized Enrichment Score
Pathway
−log10(padj)
HNSCC B cell
UBD
CYP4X1
FDCSP
TMPRSS2
C8orf4
HNSCC−B cell: Hallmark & GO pathways NES from GSEA
GOBP_COLLAGEN_CATABOLIC_PROCESS
GOBP_CELL_SUBSTRATE_JUNCTION_ORGANIZATION
GOBP_EXTERNAL_ENCAPSULATING_STRUCTURE_ORGANIZATION
HALLMARK_EPITHELIAL_MESENCHYMAL_TRANSITION
HNSCC myocyte
HNSCC−myocyte: Hallmark & GO pathways NES from GSEA
HALLMARK_INTERFERON_ALPHA_RESPONSE
HNSCC ~Fibroblast
HNSCC−−Fibroblast: Hallmark & GO pathways NES from GSEA
HNSCC Macrophage
CXCL10
PSMB10
UBD
PSMB9
TAP1
CXCL11
PSME2
SCUBE1
IFI35
UBE2L6
HNSCC−Macrophage: Hallmark & GO pathways NES from GSEA
HNSCC Endothelial
DTL
IQGAP3
CENPF
KIF11
CDCA8
RAD54L
FANCI
TOP2A
ASF1B
UHRF1
S100A12
LCE3E
JUP
TMEM79
SULT2B1
KRTDAP
IVL
SBSN
FAM25A
SPRR1B
HNSCC−Endothelial: Hallmark & GO pathways NES from GSEA
HNSCC T cell
UBD
CXCL10
MOV10
PSMB10
TRIM14
PSMB9
INPP5B
SLC18B1
RTP4
CXCL11
IGFL3
SEPT5
CDA
KLC3
HNSCC−T cell: Hallmark & GO pathways NES from GSEA
HNSCC Dendritic
UBD
CXCL10
TAP1
PSMB10
PSMB9
CXCL11
PSME2
PSME1
HNSCC−Dendritic: Hallmark & GO pathways NES from GSEA
HNSCC Mast
HNSCC−Mast: Hallmark & GO pathways NES from GSEA

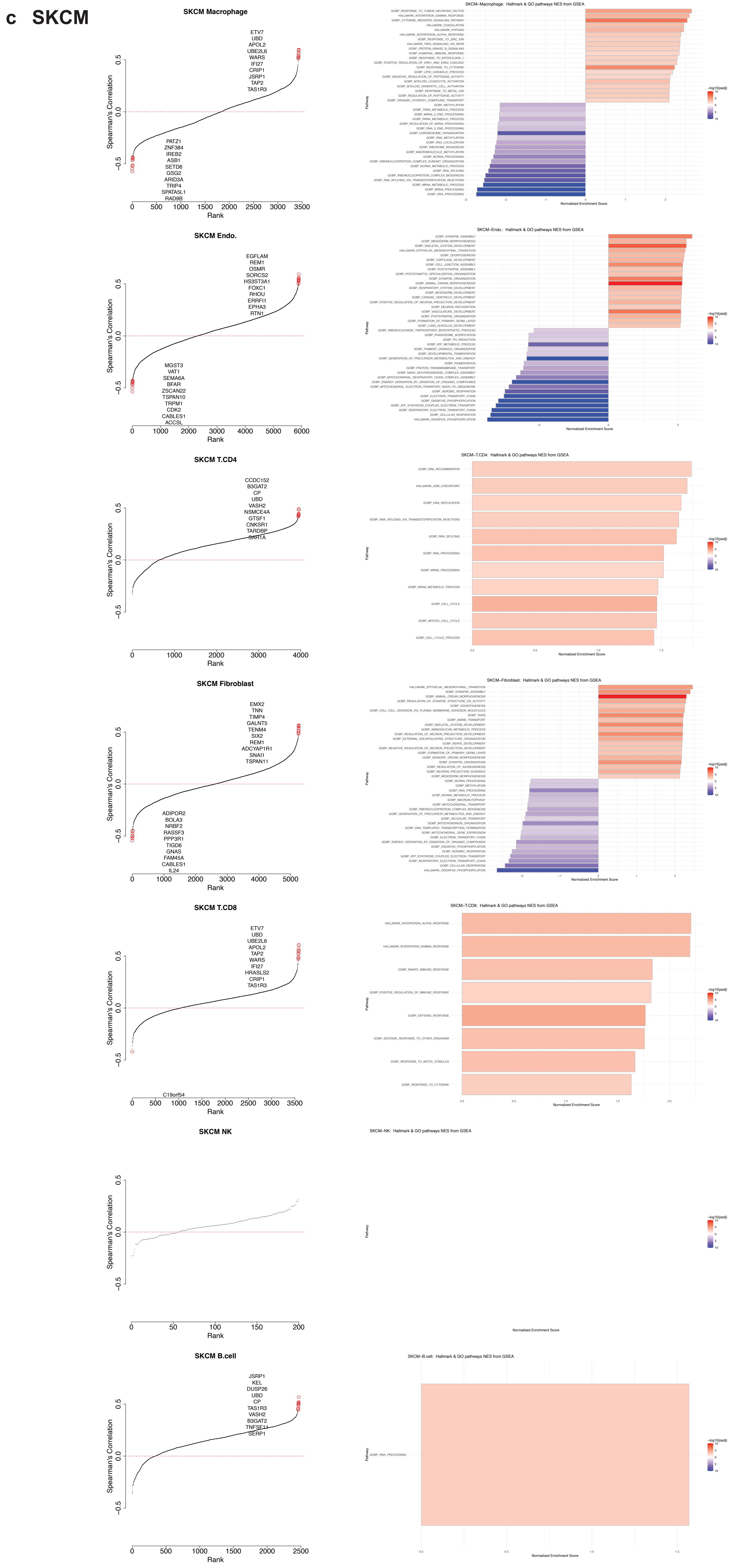

Supplementary Fig. 4 | Correlation between malignant cell gene expression and non-malignant cell fraction in three tumor types: TCGA-GBM, TCGA-HNSCC, and TCGA-SKCM. Rank-ordered plots show Spearman's rank correlation between gene expression in malignant cells inferred by BayesPrism and fractions of each non-malignant cell type in each tumor type. The top 10 positive and negative outlier genes with correlations > 0.4 are marked in red. Barplot shows the normalized gene set enrich-ment score (NES) for gene sets with the top 20 highest absolute NES, using correlations shown in the corresponding rank-ordered plot as the input.

References

1. Ding, J. *et al.* Systematic comparison of single-cell and single-nucleus RNA-sequencing methods. *Nat. Biotechnol.* **38**, 737–746 (2020).
2. Yuan, J. *et al.* Single-cell transcriptome analysis of lineage diversity in high-grade glioma. *Genome Medicine* vol. 10 (2018).
3. Neftel, C. *et al.* An Integrative Model of Cellular States, Plasticity, and Genetics for Glioblastoma. *Cell* **178**, 835–849.e21 (2019).
4. Erichson, N. B., Voronin, S., Brunton, S. L. & Kutz, J. N. Randomized Matrix Decompositions Using R. *Journal of Statistical Software, Articles* **89**, 1–48 (2019).
5. Newman, A. M. *et al.* Determining cell type abundance and expression from bulk tissues with digital cytometry. *Nat. Biotechnol.* **37**, 773–782 (2019).
6. Schafflick, D. *et al.* Integrated single cell analysis of blood and cerebrospinal fluid leukocytes in multiple sclerosis. *Nat. Commun.* **11**, 247 (2020).
7. Fisher, R. A. Frequency Distribution of the Values of the Correlation Coefficient in Samples from an Indefinitely Large Population. *Biometrika* **10**, 507–521 (1915).
8. Fisher, R. A. On the'probable error'of a coefficient of correlation deduced from a small sample. *Metron* **1**, 1–32 (1921).
9. Atul Sethi*, M. S. & Papasaikas***, P. BC2 Single Cell Tutorial. https://ppapasaikas.github.io/BC2_SingleCell/ (2017).
10. Hah, N. *et al.* A rapid, extensive, and transient transcriptional response to estrogen signaling in breast cancer cells. *Cell* **145**, 622–634 (2011).
11. Bergers, G. & Song, S. The role of pericytes in blood-vessel formation and maintenance.

Neuro. Oncol. **7**, 452–464 (2005).

12. Goldstein, L. J., Chen, H., Bauer, R. J., Bauer, S. M. & Velazquez, O. C. Normal human fibroblasts enable melanoma cells to induce angiogenesis in type I collagen. *Surgery* **138**, 439–449 (2005).
13. Linsley, P. S., Speake, C., Whalen, E. & Chaussabel, D. Copy number loss of the interferon gene cluster in melanomas is linked to reduced T cell infiltrate and poor patient prognosis. *PLoS One* **9**, e109760 (2014).
14. Lake, B. B. *et al.* Integrative single-cell analysis of transcriptional and epigenetic states in the human adult brain. *Nat. Biotechnol.* **36**, 70–80 (2018).
15. Byrd, R. H., Lu, P., Nocedal, J. & Zhu, C. A Limited Memory Algorithm for Bound Constrained Optimization. *SIAM J. Sci. Comput.* **16**, 1190–1208 (1995).
16. Verhaak, R. G. W. *et al.* Integrated genomic analysis identifies clinically relevant subtypes of glioblastoma characterized by abnormalities in PDGFRA, IDH1, EGFR, and NF1. *Cancer Cell* **17**, 98–110 (2010).
17. Guilhamon, P. *et al.* Single-cell chromatin accessibility profiling of glioblastoma identifies an invasive cancer stem cell population associated with lower survival. *Elife* **10**, (2021).
18. Puram, S. V. *et al.* Single-Cell Transcriptomic Analysis of Primary and Metastatic Tumor Ecosystems in Head and Neck Cancer. *Cell* **171**, 1611–1624.e24 (2017).
19. Tirosh, I. *et al.* Dissecting the multicellular ecosystem of metastatic melanoma by single-cell RNA-seq. *Science* **352**, 189–196 (2016).
20. Puchalski, R. B. *et al.* An anatomic transcriptional atlas of human glioblastoma. *Science* **360**, 660–663 (2018).
21. Darmanis, S. *et al.* A survey of human brain transcriptome diversity at the single cell level. *Proc. Natl. Acad. Sci. U. S. A.* **112**, 7285–7290 (2015).
