# Supplementary material for: Cell type and gene expression deconvolution with BayesPrism enables Bayesian integrative analysis across bulk and single-cell RNA sequencing in oncology
Source: Nat Cancer. 2022 Apr 25;3(4):505–17. doi: 10.1038/s43018-022-00356-3 (PMC9046084; doi:10.1038/s43018-022-00356-3)
Supplement: Supplementary file 2 — Reporting Summary [file 43018_2022_356_MOESM2_ESM.pdf]

nature research

Corresponding author(s): Tinyi Chu and Charles G. Danko

Last updated by author(s): Feb 2, 2022

Reporting Summary

Nature Research wishes to improve the reproducibility of the work that we publish. This form provides structure for consistency and transparency in reporting. For further information on Nature Research policies, see Authors & Referees and the Editorial Policy Checklist.

Statistics

For all statistical analyses, confirm that the following items are present in the figure legend, table legend, main text, or Methods section.

n/a	Confirmed	
☐	☒	The exact sample size (*n*) for each experimental group/condition, given as a discrete number and unit of measurement
☐	☒	A statement on whether measurements were taken from distinct samples or whether the same sample was measured repeatedly
☐	☒	The statistical test(s) used AND whether they are one- or two-sided<br>*Only common tests should be described solely by name; describe more complex techniques in the Methods section.*
☐	☒	A description of all covariates tested
☐	☒	A description of any assumptions or corrections, such as tests of normality and adjustment for multiple comparisons
☐	☒	A full description of the statistical parameters including central tendency (e.g. means) or other basic estimates (e.g. regression coefficient) AND variation (e.g. standard deviation) or associated estimates of uncertainty (e.g. confidence intervals)
☐	☒	For null hypothesis testing, the test statistic (e.g. *F*, *t*, *r*) with confidence intervals, effect sizes, degrees of freedom and *P* value noted<br>*Give P values as exact values whenever suitable.*
☐	☒	For Bayesian analysis, information on the choice of priors and Markov chain Monte Carlo settings
☒	☐	For hierarchical and complex designs, identification of the appropriate level for tests and full reporting of outcomes
☐	☒	Estimates of effect sizes (e.g. Cohen's *d*, Pearson's *r*), indicating how they were calculated

Our web collection on statistics for biologists contains articles on many of the points above.

Software and code

Policy information about availability of computer code

Data collection	R/Bioconductor package TCGAbiolinks (v 2.10.5)
Data analysis	DESeq2 (v 1.22.2), MCMCpack (v 1.4-4), nmle (v 3.1-141), GSVA(v 1.30.0), rms (v 5.1-3.1), survminer (v 0.4.6), survival (v 2.44-1.1), scran(v 1.20.1), fgsea(v 1.18.0), NMF(v 0.30.1), rsvd(v 1.0.5), PhenoGraph(v 1.5.2), SCDC(v 0.0.0.9000), MuSiC(v 0.2.0), BisqueRNA(v 1.0.5), bseqsc(v 1.0), TED(v 1.3)

For manuscripts utilizing custom algorithms or software that are central to the research but not yet described in published literature, software must be made available to editors/reviewers. We strongly encourage code deposition in a community repository (e.g. GitHub). See the Nature Research guidelines for submitting code & software for further information.

Data

Policy information about availability of data

All manuscripts must include a data availability statement. This statement should provide the following information, where applicable:

- Accession codes, unique identifiers, or web links for publicly available datasets
- A list of figures that have associated raw data
- A description of any restrictions on data availability

The authors declare that the data supporting the findings of this study are all publicly available, with accession codes included.

October 2018

Field-specific reporting

Please select the one below that is the best fit for your research. If you are not sure, read the appropriate sections before making your selection.

☒ Life sciences ☐ Behavioural & social sciences ☐ Ecological, evolutionary & environmental sciences

For a reference copy of the document with all sections, see nature.com/documents/nr-reporting-summary-flat.pdf

Life sciences study design

All studies must disclose on these points even when the disclosure is negative.

Sample size	No sample-size estimates were performed to detect a pre-specified effect size. Statistical analyses were performed on a TCGA and IVY-GAP datasets, where sample sizes are large, and all of which were analyzed and interpreted using statistically appropriate techniques, as described in Online Methods.
Data exclusions	No
Replication	All attempts at replication were successful. Our software are robust to randomness of MCMC sampling. Two random seeds were tested over the benchmark dataset, and yielded near-identical results.
Randomization	Sample groups were determined according known or predetermined biological or clinical phenotypes (e.g., TCGA-GBM subtypes, and IVY-GAP anatomical structures). No randomization was applied.
Blinding	Not applicable. All data were collected by published literature based on clinical phenotypes.

Reporting for specific materials, systems and methods

We require information from authors about some types of materials, experimental systems and methods used in many studies. Here, indicate whether each material, system or method listed is relevant to your study. If you are not sure if a list item applies to your research, read the appropriate section before selecting a response.

Materials & experimental systems

n/a	Involved in the study	
☒	☐	Antibodies
☒	☐	Eukaryotic cell lines
☒	☐	Palaeontology
☒	☐	Animals and other organisms
☒	☐	Human research participants
☒	☐	Clinical data

Methods

n/a	Involved in the study	
☒	☐	ChIP-seq
☒	☐	Flow cytometry
☒	☐	MRI-based neuroimaging
